# Paralemnanoids A–K, new norsesquiterpenoids and sesquiterpenoids with hepatoprotective activity from the soft coral *Paralemnalia* sp.

**DOI:** 10.1007/s42995-025-00339-0

**Published:** 2026-03-30

**Authors:** Yanfang Pei, Yuan Zong, Jixiang Xu, Huiyue Hou, Hengyi Xu, Yun Chen, Tianyun Jin, Pinglin Li

**Affiliations:** 1 Key Laboratory of Marine Drugs, Chinese Ministry of Education, School of Medicine and Pharmacy, Ocean University of China, Qingdao, 266003 China; 2 Laboratory for Marine Drugs and Bioproducts, Qingdao Marine Science and Technology Center, Qingdao, 266237 China; 3 Shandong Academy of Chinese Medicine, Jinan, 250014 China; 4 State Key Laboratory of Bioactive Substances and Functions of Natural Medicines, Institute of Medicinal Biotechnology, Chinese Academy of Medical Sciences & Peking Union Medical College, Beijing, 100050 China; 5 Center for Marine Biotechnology and Biomedicine, Scripps Institution of Oceanography, University of California San Diego, La Jolla, CA 92093-0204 USA

**Keywords:** Soft coral, *Paralemnalia* sp., Nardosinanes, Configuration determination, Hepatoprotective activity, X-ray diffraction

## Abstract

Eleven nardosinane-type compounds, including a norsesquiterpenoid featuring a novel carbon skeleton designated as paralemnanoid A (**1**), and ten additional norsesquiterpenoids identified as paralemnanoids B − K (**2** − **11**), were isolated from the South China Sea soft coral *Paralemnalia* sp.. Comprehensive structural elucidation of compounds **1** − **11** was achieved through an integrated analytical approach, combining including NMR spectroscopy, HRESIMS, single-crystal X-ray diffraction analysis, DP4 + probability assessments, ECD calculations, and literature comparisons. Furthermore, we proposed that the novel poly-isoprenoid scaffold paralemnanoid A (**1**) originates from paralemnanoid B (**2**) through sequential oxidative modifications followed by non-enzymatic skeletal rearrangements. The hepatoprotective effects of compounds **1** − **11** were assessed through bioassays, revealing that paralemnanoids A (**1**) and E (**5**) exhibited moderate hepatoprotective activity at a concentration of 20 μmol/L in the zebrafish model. This study expands the chemical diversity of marine-derived nardosinane-type terpenoids and provides new insights into their potential therapeutic applications.

**Supplementary Information:**

The online version contains supplementary material available at https://doi.org/10.1007/s42995-025-00339-0.

## Introduction

The liver functions as the central organ for drug metabolism and biotransformation, serving a pivotal defensive role in safeguarding the body against toxic xenobiotics (Pandey et al. 2023). Alcohol abuse is one of the most significant causes of liver diseases globally (Kang et al. 2013). Therefore, research into novel liver-protective agents against alcohol-induced liver damage holds substantial significance.

Soft corals belonging to the genus *Paralemnalia* (Nephtheidae) have been established as a rich source of a variety of sesquiterpenoids (Bishara et al. 2008; Huang et al. 2006; Izac et al. 1982) and norsesquiterpenoids (Su et al. 2004). These components predominantly include nardosinane-type (Wang et al. 2010), neolemnane-type (Huang et al. 2007; Zong et al. 2024), africanane-type (Izac et al. 1982) and ylangene-type (Cheng et al. 2010). These metabolites demonstrate diverse biological activities, encompassing antitumor (Lee et al. 2017; Wang et al. 2010), antibacterial (Liu et al. 2022), neuroprotective (Tseng et al. 2013), antiviral (Liu et al. 2020; Nagahawatta et al 2024), antiangiogenic (Liu et al. 2020; Wang et al. 2019), angiogenesis-promoting (Han et al. 2021) and anti-Alzheimer’s disease activity (Wu et al. 2018; Zong et al. 2024). Furthermore, nardosinane-type sesquiterpenes are characterized by a 6-isopropyl-dimethyldecahydronaphthalene core, with structural diversity predominantly arising from oxidation or cyclization (Wang et al. 2019) at various sites along the backbone. Consequently, nardosinane-type sesquiterpenes have garnered significant research interest owing to their remarkable structural diversity and multifaceted bioactivities.

**Fig. 1:**
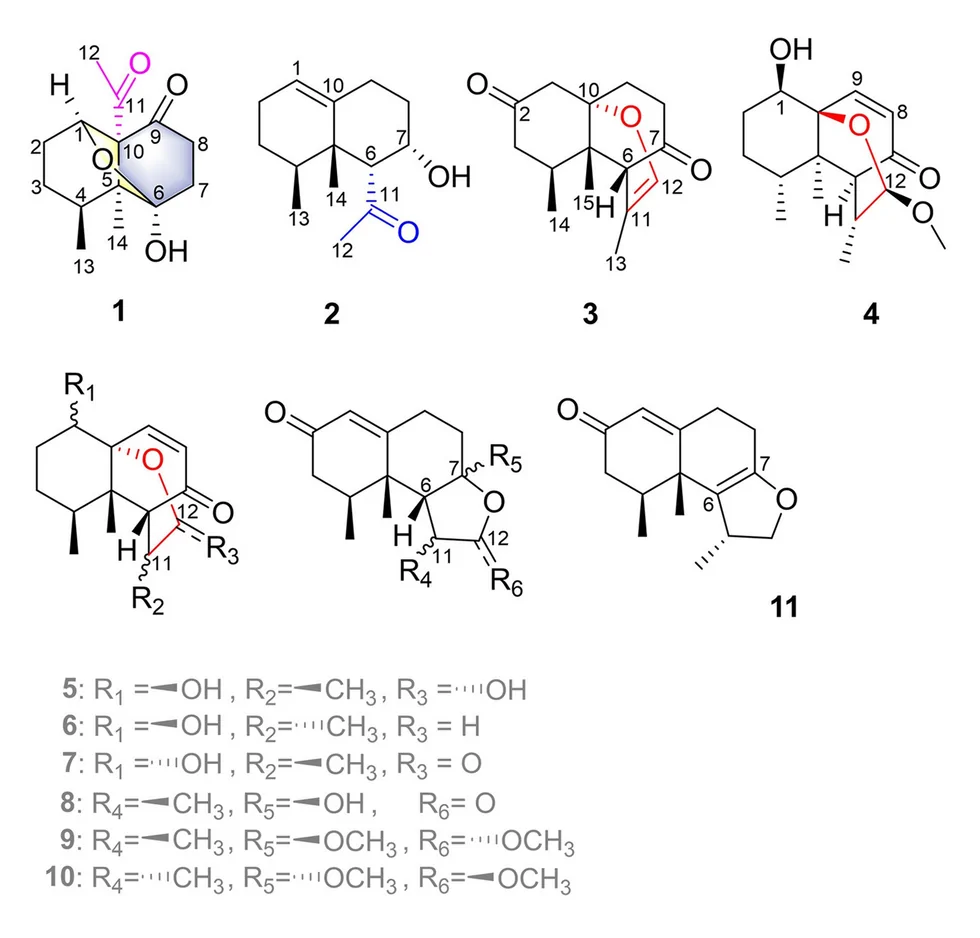
Structures of paralemnanoids A − K (**1** − **11**)

In our ongoing exploration of bioactive constituents from South China Sea *Paralemnalia* sp., we isolated eleven novel sesquiterpenoids, designated as paralemnanoids A − K (**1** − **11**) (Fig. 1). Among these compounds, paralemnanoid A (**1**) emerges as a novel nardosinane-type terpenoid characterized by a unique skeleton rearrangement. We hypothesize that the unique structural features of **1** arise from the cleavage of a C–C bond and the subsequent formation of a new C–C bond within the isopentenyl skeleton, resembling an acetyl migration from C-6 to C-10. Paralemnanoid B (**2**) represents a novel nardosinane-type norsesquiterpene and is postulated to serve as the precursor of compound **1**. Paralemnanoids C − G (**3** − **7**) feature a 6/6/6 tricyclic framework incorporating either a cyclic 10,12-olide or a cyclic 10,12-ether moiety. Notably, the absolute configuration of compound **4** (1*S*, 4*R*, 5*R*, 6*S*, 10*S*, 11*R*, 12*R*) is exceptionally rare within nardosinane-type structures. Paralemnanoids H − K (**8** − **11**) exhibit a distinctive 6/6/5 tricyclic scaffold featuring a cyclic 7,12-olide or 7,12-ether moiety (Yang et al. 2022). This study comprehensively details the isolation, structural characterization (supported by spectroscopic and crystallographic analyses), putative biosynthetic pathways, and hepatoprotective potential of these compounds.

## Materials and methods

### General experimental procedures

Optical rotations were measured on a Jasco P-1020 digital polarimeter (Jasco, Tokyo, Japan). The UV spectra were acquired on a Beckman DU640 spectrophotometer (Beckman Ltd., Shanghai, China). ECD spectra were obtained on a Jasco J-810 spectropolarimeter (Jasco, Tokyo, Japan). NMR spectra were using tetramethyl silane in the solvent of CDCl_3_ (*δ*_H_ 7.26 and *δ*_C_ 77.16) as an internal standard obtained on Agilent 500 MHz (Agilent, California, USA), (^1^H, 500 MHz; ^13^C, 126 MHz) or the Bruker AVANCE NEO 400 (^1^H, 400 MHz; ^13^C, 101 MHz), (Bruker, Faellanden, Switzerland). HRESIMS data were measured on Micromass Q-TOF Ultima GLOBAL GAA076LC mass spectrometers (Autospec-Ultima-TOF, Waters, Shanghai, China). Crystallographic data were acquired on a Bruker APEX-II CCD diffractometer (Bruker, Faellanden, Switzerland) equipped with graphite-monochromatized Cu Kα radiation. Semi-preparative high-pres-sure liquid chromatograph (HPLC) was performed using a Waters 1525 pump (Waters, Singapore) equipped with a 2998 photodiode array detector and a SilGreen C18 column (10 mm × 250 mm, 5 μm). Chiral HPLC analysis and resolution were conducted on a chiral analytical column (Daicel, Shanghai, China). Column chromatography was used with silica gel (200–300 mesh, 300–400 mesh, and silica gel H, Qingdao Marine Chemical Factory, Qingdao, China), ODS silica gel (50 μmol/L, Merck, Germany). Thin layer chromatography (TLC) analyses were used with precoated silica gel plates (GF254, Qingdao, China) and chromogenic reagents were 20% H_2_SO_4_ in EtOH.

### Soft coral material

The marine soft coral *Paralemnalia* sp. was collected from Yongle Islands of Xisha Island of the South China Sea in 2013, and after collection the specimen was frozen immediately. The soft coral was identified by Ping-Jyun Sung, Institute of Marine Biotechnology, Museum of Marine Biology & Aquarium, Pingtung 944, Taiwan. The voucher specimen (No. xs-2013–11) was deposited at the State Key Laboratory of Marine Drugs, Ocean University of China, People’s Republic of China.

### Extraction and purification

A frozen specimen *Paralemnalia* sp. (6.0 kg, wet weight) was cut into pieces and then extracted with MeOH five times (3 days each time) at room temperature to yield crude extract after evaporation in vacuo. Then the crude extract was suspended in distilled water (1.5 L) and partitioned with same volume of EtOAc (five times) to yield 60.0 g of EtOAc solvent extract. This extract was subjected to silica gel vacuum column chromatography (VLC) and eluted with a gradient of petroleum ether/acetone (50:1 to 1:1, v/v) and subsequently eluted with a gradient of CH_2_Cl_2_/MeOH (10:1 to 1:1, v/v) to obtain eleven fractions (Frs.1–11). Each fraction was detected by TLC.

Fr. 7 (5.3 g) was subjected to silica gel CC (petroleum/ethyl acetate from 70:1 to 1:1, v/v) to yield six subfractions (Fr.71-Fr.76). Fr.74 (440 mg) was then purified by semi-preparative HPLC (ODS, 5 µm, 250 mm × 10 mm; methanol/water, 70:30, v/v; 2.0 mL/min) to afford compound **4** (1.0 mg). Fr. 75 (400 mg) was then purified by semi-preparative HPLC (ODS, 5 µm, 250 mm × 10 mm; methanol/water, 70:30, v/v; 2.0 mL/min) to afford compound **2** (7.0 mg). Fr.76 (500 mg) was purified by semi-preparative HPLC (ODS, 5 µm, 250 mm × 10 mm; methanol/water, 50:50, v/v; 2.0 mL/min) to afford compound **6** (1.5 mg) and compound **11** (2.4 mg).

Fr. 8 (2.7 g) was subjected to silica gel CC (petroleum/ethyl acetate from 40:1 to 1:1, v/v) to yield seven subfractions (Fr.81-Fr.87). Fr.84 (1.6 g) was also subjected to silica gel CC (petroleum/acetone, from 60:1 to 1:1, v/v) to give four subfractions (Fr.841-Fr.844), Fr.843 (750 mg) was then purified by semi-preparative HPLC (ODS, 5 µm, 250 mm × 10 mm; methanol/water, 65:35, v/v; 2.0 mL/min) to afford compound **5** (1.5 mg) and compound **10** (1.3 mg). Fr.87 (460 mg) was purified by semi-preparative HPLC (ODS, 5 µm, 250  mm × 10 mm; methanol/water, 40:60, v/v; 2.0 mL/min) to afford compound **3** (3 mg).

Fr. 9 (840 mg) was subjected to silica gel CC (petroleum/acetone, from 40:1 to 1:1, v/v) to yield two subfractions (Fr.91-Fr.92). Fr. 92 (480 mg) was purified by semi-preparative HPLC (ODS, 5 µm, 250 mm × 10 mm; methanol/water, 60:40, v/v; 2.0 mL/min) to afford compound **9** (1.2 mg).

Fr. 10 (6.3 g) was subjected to silica gel CC (petroleum/acetone, from 20:1 to 1:1, v/v) to yield nine subfractions (Fr.101-Fr.109). Fr. 102 (210 mg) was purified by semi-preparative HPLC (ODS, 5 µm, 250 mm × 10 mm; methanol/water, 70:30, v/v; 2.0 mL/min) to afford compound **7** (1.5 mg), and compound **8** (1.3 mg). Fr. 109 (530 mg) was purified by semi-preparative HPLC (ODS, 5 µm, 250 mm × 10 mm; methanol/water, 70:30, v/v; 2.0 mL/min) to obtain compound **1** (3 mg).

Paralemnanoid A (**1**), colorless crystal; [*α*]^25^_D_ =  + 24.05 (*c* 0.1 MeOH); UV (MeOH): *λ*_max_ (log*ε*) 197(1.24), 205 (1.83); ^1^H and ^13^C NMR data, Table 1. HRESIMS *m/z* 253.1438 [M + H]^+^: calcd for C_14_H_21_O_4_, 253.1434.

**Table 1:** ^1^H and ^13^C NMR Data in CDCl_3_ for Compounds **1**–**3**

No	1		2		3	
	*δ*_C_^*a*^	*δ*_H_^*c*^, mult.(*J* in Hz)	*δ*_C_^*b*^	*δ*_H_^*d*^, mult.(*J* in Hz)	*δ*_C_^*b*^	*δ*_H_^*d*^, mult.(*J* in Hz)
1	77.5, CH	4.62 d (5.0)	126.7, CH	5.50 m	49.0, CH_2_	2.56 dd (15.1, 1.6); 2.47, m
2	28.5, CH_2_	1.98 m; 1.57 m	25.6, CH_2_	2.04 m; 1.90 m	207.2, C	
3	27.7, CH_2_	2.06 m	26.8, CH_2_	1.38 m	44.9, CH_2_	2.29 m; 2.19 m
4	35.0, CH	2.08 m	36.0, CH	1.52 m	32.2, CH	2.43 m
5	48.6, C		41.8, C		37.9, C	
6	109.6, C		61.5, CH	3.37 d (5.6)	57.7, CH	2.73 s
7	34.3, CH2	2.17 ddd	69.5, CH	4.23 dt (11.8, 5.3)	210.1, C	
		(13.1, 10.1, 7.1)				
		1.90 dd (13.1, 10.1)				
8	36.7, CH_2_	2.78 dt (17.0, 10.1)	30.6, CH_2_	1.95 m; 1.75 m	32.7, CH_2_	2.89 dt (16.2, 10.0); 2.23 m
		2.42 dd (17.0, 7.1)				
9	209.7, C		30.4, CH_2_	2.37 m; 2.25 m	35.8, CH_2_	2.15 m; 2.12 m
10	77.2, C		138.1, C		80.6, C	
11	204.8, C		212.2, C		104.7, C	
12	30.6, CH_3_	2.11 s	36.0, CH_3_	2.22 s	140.0, CH	6.27 s
13	16.9, CH_3_	1.13 d (6.7)	16.0, CH_3_	1.01 s	16.5, CH_3_	1.51 d (1.5)
14	18.0, CH_3_	1.21 s	20.1, CH_3_	0.94 d (6.8)	15.0, CH_3_	0.86 d (6.8)
15					14.8, CH_3_	1.0 s

^a^Recorded at 500 MHz in CDCl_3_

^b^Recorded at 400 MHz in CDCl_3_

^c^Recorded at 126 MHz in CDCl_3_

^d^Recorded at 101 MHz in CDCl_3_

Paralemnanoid B (**2**), colorless crystal; [*α*]^25^_D_ =  + 22.70 (*c* 0.05 MeOH); UV (MeOH): *λ*_max_ (log*ε*) 204 (1.52), 225 (2.24); ^1^H and ^13^C NMR data, Table 1. HRESIMS *m/z* 223.1698 [M + H]^+^: calcd for C_14_H_23_O_2_, 223.1693.

Paralemnanoid C (**3**), colorless crystal; [*α*]^25^_D_ =  + 65.38 (*c* 0.1 MeOH); UV (MeOH): *λ*_max_ (log*ε*) 196 (1.02), 201 (2.17); ^1^H and ^13^C NMR data, Table 1. HRESIMS *m/z* 251.1646 [M + H]^+^: calcd for C_15_H_23_O_3_, 251.1642.

Paralemnanoid D (**4**), colorless crystal; [*α*]^25^_D_ = -23.05 (*c* 0.1 MeOH); UV (MeOH): *λ*_max_ (log*ε*) 197(1.27), 203 (1.88); ^1^H and ^13^C NMR data, Table 2. HRESIMS *m/z* 281.1751 [M + H]^+^: calcd for C_16_H_25_O_4_, 281.1747.

**Table 2:** ^1^H and ^13^C NMR Data in CDCl_3_ for Compounds **4**–**7**

No		**4**		**5**		**6**		**7**
	*δ*_C_^*a*^	*δ*_H_^*d*^, mult.(*J* in Hz)	*δ*_C_^*b*^	*δ*_H_^*e*^, mult.(*J* in Hz)	*δ*_C_^*a*^	*δ*_H_^*d*^, mult.(*J* in Hz)	*δ*_C_^*c*^	*δ*_H_^*f*^, mult.(*J* in Hz)
1	73.2, CH	3.87 d (2.7)	73.0, CH	3.86 s	73.5, CH	3.82 s	68.7, CH	3.68 dd (11.6, 5.2)
2	29.6, CH_2_	2.24 m, 1.66 m	29.5, CH_2_	2.22 m, 1.65 m	29.6, CH_2_	2.18 m, 1.64 m	30.3, CH_2_	2.05 m, 1.76 m
3	24.1, CH_2_	1.67 m, 1.38 m	24.1, CH_2_	1.65 m, 1.68 m	24.2, CH_2_	1.64 m, 1.59 m	27.8, CH_2_	1.66 m, 1.57 m
4	30.2, CH	2.58 m	30.4, CH	2.58 m	32.1, CH	2.68 m	30.4, CH	2.09 m
5	41.4, C		41.4, C		40.9, C		40.3, C	
6	57.0, CH	2.37 d (4.7)	58.8, CH	2.39 d (4.9)	60.0, CH	2.37 m	56.1, CH	2.67 d (7.2)
7	201.8, C		201.7, C		203.2, C		199.8, C	
8	133.9, CH	6.30 d (10.0)	132.9, CH	6.29 d (10.0)	133.4, CH	6.38 d (9.9)	128.8, CH	7.38 d (10.0)
9	143.6, CH	6.56 d (10.0)	145.1, CH	6.57 d (10.0)	144.5, CH	6.42 d (9.9)	145.8, CH	6.13 d (10.0)
10	76.1, C		76.4, C		75.3, C		83.8, C	
11	32.5, CH	2.18 m	34.7, CH	2.12 m	40.0, CH	1.66 m	35.4, CH	2.96 p (7.3)
12	103.1, CH	4.23 d (9.6)	96.3, CH	4.60 d (9.5)	64.7, CH_2_	3.94 dd (12.3, 6.0)	172.9, C	
						3.55 d (12.4)		
13	14.8, CH_3_	0.84 d (6.9)	14.9, CH_3_	0.90 d (6.9)	22.4, CH_3_	1.35 d (7.7)	14.6, CH_3_	1.15 d (7.3)
14	13.9, CH_3_	0.75 d (7.0)	13.9, CH_3_	0.76 d (7.0)	15.0, CH_3_	0.84 d (6.9)	13.6, CH_3_	0.85 d (6.9)
15	15.4, CH_3_	1.04 s	16.7, CH_3_	1.05 s	18.2, CH_3_	1.03 s	15.4, CH_3_	1.03 s
16-OMe	56.8, CH_3_	3.42 s						

^a^Recorded at 600 MHz in CDCl_3_

^b^Recorded at 400 MHz in CDCl_3_

^b^Recorded at 500 MHz in CDCl_3_

^c^Recorded at 151 MHz in CDCl_3_

^d^Recorded at 101 MHz in CDCl_3_

^c^Recorded at 126 MHz in CDCl_3_

Paralemnanoid E (**5**), colorless crystal; [*α*]^25^_D_ =  + 45.81 (*c* 0.1 MeOH); UV (MeOH): *λ*_max_ (log*ε*) 201 (1.47), 242 (2.46); ^1^H and ^13^C NMR data, Table 2. HRESIMS *m/z* 267.1597 [M + H]^+^: calcd for C_15_H_23_O_4_, 267.1593.

Paralemnanoid F (**6**), colorless crystal; [*α*]^25^_D_ =  + 5.73 (*c* 0.1 MeOH); UV (MeOH): *λ*_max_ (log*ε*) 195 (1.95), 218 (2.09); ^1^H and ^13^C NMR data, Table 2. HRESIMS *m/z* 251.1646 [M + H]^+^: calcd for C_15_H_23_O_3_, 251.1642.

Paralemnanoid G (**7**), colorless crystal; [*α*]^25^_D_ =  + 28.83 (*c* 0.1 MeOH); UV (MeOH): *λ*_max_ (log*ε*) 201(1.05), 245 (1.97); ^1^H and ^13^C NMR data, Table 2. HRESIMS *m/z* 265.1433 [M + H]^+^: calcd for C_15_H_21_O_4_, 265.1434.

Paralemnanoid H (**8**), colorless crystal; [*α*]^25^_D_ = -32.52 (*c* 0.1 MeOH); UV (MeOH): *λ*_max_ (log*ε*) 196(1.07), 229 (1.58); ^1^H and ^13^C NMR data, Table 3. HRESIMS *m/z* 265.1439 [M + H]^+^: calcd for C_15_H_21_O_4_, 265.1434.

**Table 3:** ^1^H and ^13^C NMR Data in CDCl_3_ for Compounds **8**–**11**

No		**8**		**9**		**10**		**11**
	*δ*_C_^*a*^	*δ*_H_^*c*^, mult.(*J* in Hz)	*δ*_C_^*a*^	*δ*_H_^*c*^, mult.(*J* in Hz)	*δ*_C_^*a*^	*δ*_H_^*c*^, mult.(*J* in Hz)	*δ*_C_^*b*^	*δ*_H_^*d*^, mult.(*J* in Hz)
1	128.2 CH	5.99 s	126.8 CH	5.97 s	126.9 CH	5.91 s	124.4 CH	5.81 s
2	197.9 C		196.7 C		198.5 C		199.4 C	
3	41.5 CH_2_	2.35 m	42.0 CH_2_	2.34 m; 2.27 m	41.8 CH_2_	2.29 m, 2.27 m	43.3 CH_2_	2.31 m
4	36.0 CH	2.23 m	36.2 CH	2.35 m	35.9 CH	2.26 m	38.7 CH	2.28 m
5	41.8 C		42.0 C		42.0 C		41.3 C	
6	56.3 CH	2.34 m	54.8 CH	2.28 m	54.9 CH	2.09 m	117.5 C	
7	105.9 C		111.4 C		108.9 C		151.7 C	
8	33.5 CH_2_	2.25 m; 2.16 m	29.7 CH_2_	2.10 m	30.8 CH_2_	2.07 m; 2.01 m	24.7 CH_2_	2.26 m
				1.97 dt (13.9, 4.5)				
9	27.9 CH_2_	2.70 m, 2.40 m	28.0 CH_2_	2.57 m, 2.48 m	28.1 CH_2_	2.64 m, 2.58 m	30.7 CH_2_	2.74 m, 2.38 m
10	165.2 C		169.8 C		169.1 C		169.9 C	
11	40.5 CH	2.37 m	43.0 CH	1.86 m	43.4 CH	1.70 m	39.3 CH	3.11 m
12	176.0 C		106.3 CH	4.75 d (5.1)	110.2 CH	4.59 d (5.7)	77.7 CH_2_	4.31 t (8.9)
								3.94 dd (8.9, 3.7)
13	18.2 CH_3_	1.42 d (6.5)	15.3 CH_3_	1.13 d (6.7)	18.8 CH_3_	1.18 d (6.7)	22.0 CH_3_	1.24 d (6.7)
14	15.8 CH_3_	1.01 d (6.6)	15.8 CH_3_	0.96 d (6.0)	16.0 CH_3_	0.96 d (6.3)	18.6 CH_3_	1.17 d (6.2)
15	19.1 CH_3_	1.33 s	19.2 CH_3_	1.33 s	18.7 CH_3_	1.20 s	20.0 CH_3_	1.28 s
16 -OMe			55.2 CH_3_	3.42 s	49.2 CH_3_	3.36 s		
17 -OMe			49.6 CH_3_	3.43 s	57.0 CH_3_	3.43 s		

^a^Recorded at 500 MHz in CDCl_3_

^b^Recorded at 600 MHz in CDCl_3_

^c^Recorded at 126 MHz in CDCl_3_

^d^Recorded at 151 MHz in CDCl_3_

Paralemnanoid I (**9**), colorless crystal; [*α*]^25^_D_ = -18.40 (*c* 0.05 MeOH); UV (MeOH): *λ*_max_ (log*ε*) 206, ^1^H and ^13^C NMR data, Table 3. HRESIMS *m/z* 295.1909 [M + H]^+^: calcd for C_17_H_27_O_4_, 295.1904.

Paralemnanoid J (**10**), colorless crystal; [*α*]^25^_D_ =  + 14.81 (*c* 0.1 MeOH); UV (MeOH): *λ*_max_ (log*ε*) 195 (1.93); ^1^H and ^13^C NMR data, Table 3. HRESIMS *m/z* 295.1907 [M + H]^+^: calcd for C_14_H_21_O_4_, 295.1904.

Paralemnanoid K (**11**), colorless crystal; [*α*]^25^_D_ =  + 12.80 (*c* 0.05 MeOH); UV (MeOH): *λ*_max_ (log*ε*) 197 (1,21), 208 (1.59); ^1^H and ^13^C NMR data, Table 3. HRESIMS *m/z* 253.1438 [M + H]^+^: calcd for C_14_H_21_O_4_, 253.1434.

### Zebrafish maintenance

Adult zebrafish were cultivated by Qilu University of Technology (Jinan, China). Transgenic zebrafish [Tg: zlyz-EGFP and T3 (L-FABP: EGFP)] expressing enhanced green fluorescent protein (EGFP) were used in this study. The conditions of the maintenance were complied with guidelines of the Organization for Economic Co-operation and Development (OECD). The zebrafish were maintained under a 14/10 h light/dark cycle at the temperature (28 ± 0.5 °C) in a closed flow-through system with charcoal-filtered tap water to ensure normal spawning.

### Alcohol induced liver injury model of zebrafish

At 3 days post fertilization (dpf), juvenile zebrafish with normal liver development were selected into 24-well plates (*n* = 10/well) in a 2 mL final volume of embryo medium, randomly divided into four groups: a control group, fresh fish water (5.0 mmol/L NaCl, 0.17 mmol/L KCl, 0.4 mmol/L CaCl_2_, 0.16 mmol/L MgSO_4_), a model group: ethanol at 1.0% concentrations, a positive drug group: silybinin (120 μg/mL), a sample group (20 μmol/L). All the groups incubated at constant temperature (28 ℃) in a light incubator and continuously for 72 h, changed the fluid daily. All treatments were performed in duplicate. Each zebrafish larva was photographed by a fluorescence microscope (OLYMPUS SZX16), and the liver fluorescence area was calculated through Image-Pro Plus software.

### X-ray crystallographic

Dissolve compound in 2 mL of anhydrous ethanol in a 5 mL beaker, and volatilized for 7–8 days to form single crystal. X-ray diffraction was performed on a Bruker APEX-II CCD diffractometer with Cu Kα radiation. Crystals were kept at 150.00 K during data collection.

### Quantum chemical calculations

The conformational analyses ofcompounds were completed with the Spartan 10 or MOE software package with the MMFF minimization force field. The resulting conformers were optimized by density functional theory (DFT) calculations at the B3LYP/6–31 + G(d,p) level in the gas phase with Gaussian 09 software. The TD-DFT method with the basis set RB3LYP/DGDZVP was used for ECD calculations for the optimized conformations, whose Boltzmann distributions of Gibbs free energies were more than 1.0%.

## Results

The frozen bodies of *Paralemnalia* sp. (6.0 kg, wet weight) were collected from Xisha Island of the South China Sea in 2013. The samples were extracted with MeOH at room temperature. Eleven new sesquiterpenoids (**1**–**11**) were isolated from the EtOAc-soluble portion.

**Fig. 2:**
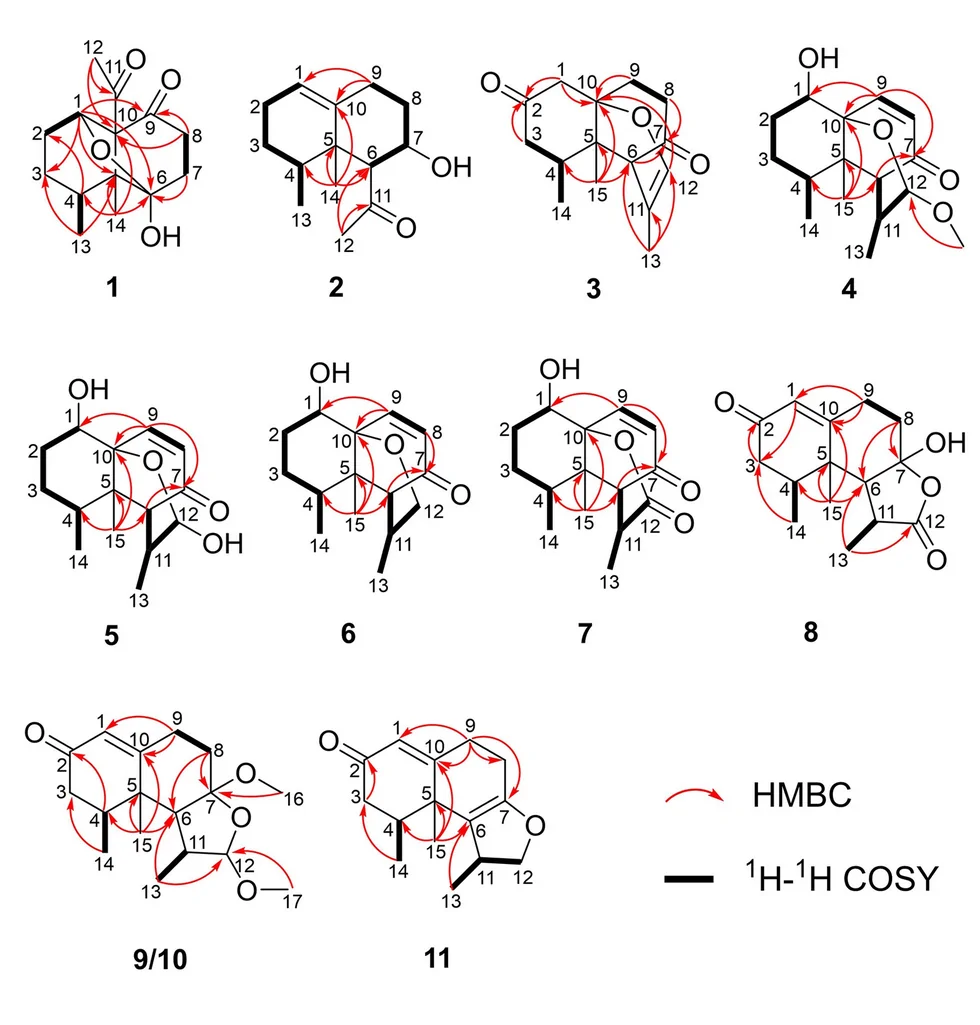
^1^H − ^1^H COSY and key HMBC correlations of paralemnanoids A–K (**1**–**11**)

Paralemnanoid A (**1**) was isolated as colorless crystals. Its molecular formula was determined to be C_14_H_20_O_4_ by HRESIMS at *m/z* 253.1438 [M + H]^+^ (calcd. for 253.1434), requiring five degrees of unsaturation. The ^1^H NMR and HSQC spectra of **1** showed the presence of two methine protons, including one oxygenated at *δ*_H_ 4.62 (H-1, 1H, d,* J* = 5.0 Hz), another one at *δ*_H_ 2.08 (H-4, 1H, m); four methylenes at *δ*_H_ 1.98 (H-2a, m), 1.57 (H-2b, m), *δ*_H_ 2.06 (H-3, 2H, m), *δ*_H_ 2.17 (H-7a, ddd, *J* = 13.1, 10.4, 7.1 Hz), 1.90 (H-7b, dd,* J* = 13.1, 10.1 Hz), *δ*_H_ 2.78 (H-8a, dt, *J* = 17.0, 10.1 Hz), 2.42 (H-8b, dd,* J* = 17.0, 7.1 Hz), and three methyl groups at *δ*_H_ 2.11 (H-12, 3H, s), *δ*_H_ 1.13 (H-13, 3H, d, *J* = 6.7 Hz) and *δ*_H_ 1.21 (H-14, 3H, s),. The ^13^C NMR (Table 1) and HSQC (Supplementary Fig. S1c) spectra revealed 14 carbon resonances containing five nonprotoned carbons (*δ*_C_ 209.7, 204.8, 109.6, 77.2, 48.6), two methine carbons (*δ*_C_ 77.5, 35.0), four methylene carbons (*δ*_C_ 36.7, 34.3, 28.5, 27.7), and three methyl carbons (*δ*_C_ 30.6, 18.0, 16.9). ^1^H − ^1^H COSY correlations of H-1/H_2_-2, H-4/H_3_-13, and H_2_-7/H_2_-8 revealed the presence of three spin systems, as well as the key HMBC correlation (Fig. 2) from H-1 to C-3/C-5/C-6/C-9, from H_2_-7 to C-6, from H_2_-8 to C-9/C-10, from H_3_-12 to C-10/C-11, from H_3_-13 to C-3/C-4/C-5, and from H_3_-14 to C-4/C-5/C-6/C-10, constructed a 4,5-dimethyl-10-acetyl-substituted bycyclic 6/6 new carbon skeleton, whereas in the typical nardosinane-type norsesquiterpenoid acetyl group substitution usually occurs at C-6. Furthermore, the key HMBC correlation from H-1 to C-6 revealed that C-1 and C-6 are close in the plane structures. Combined with the deshielded shifts (C-1, *δ*_C_ 77.5; C-6, *δ*_C_ 109.6) and HRESIMS data, we assigned an oxygen bridge between C-1 and C-6, along with a hydroxyl group in C-6. Thus, the planar structure of **1** was thus established as shown (Fig. 2).

**Fig. 3:**
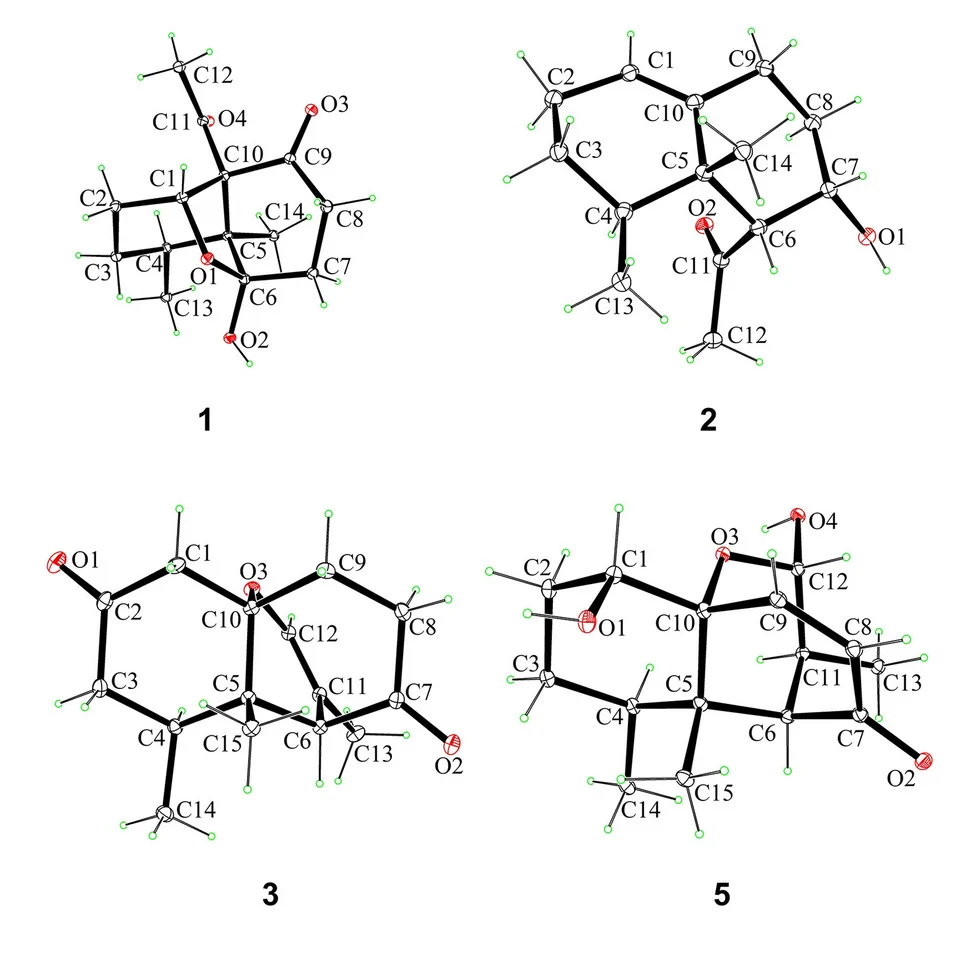
X-ray crystal structures of **1**–**3** and **5**

Fortunately, single crystals suitable for X-ray crystallographic analysis were obtained from MeOH solution. The absolute stereochemical structure of **1** was determined to be 1*S*, 4*S*, 5*S*, 6*S*, 10*S* (Fig. 3), with a Flack parameter of − 0.13 (6) (Table S1, CCDC: 2,410,571). Finally, these data suggested that **1** was a novel nardosinane-derivate featuring a 6/6/5 tricyclic framework.

Paralemnanoid B (**2**) was isolated as colorless oil, with molecular formula C_14_H_22_O_2_, and determined by HRESIMS at *m/z* 223.1698 [M + H]^+^ (calcd. for 223.1693), requiring four degrees of unsaturation. The ^1^H − ^1^H COSY correlations (Fig. 2) for H-1/H_2_-2/H_2_-3/H-4/H3-13, and H-6/H-7/H_2_-8/H_2_-9, together with the HMBC correlations (Fig. 2) from H_2_-9 to C-1/C-10, H_3_-12 to C-6/C-11, H_3_-14 to C-4/C-5/C-6/C-10, established the nornardosinane-type sesquiterpenoid. In the NOESY spectra, the correlations between H-7 and H_3_-14, together with those between H_3_-13 and H-6/H_3_-14, revealed these groups were coplanar. The ECD spectrum of **2** showed a positive cotton effect at 216 nm (Δ*ε* =  + 38.1) and a negative cotton effect at 295 nm (Δ*ε* =—11.8). The absolute configuration of **2** was defined as 4*S*, 5*S*, 6*R*, 7*S* based on the ECD calculations (Supplementary Figs. S12a). Single crystals of **2** were successfully obtained from the methanol solution. The result of single-crystal X-ray diffraction analysis (Fig. 3, CDCC: 2,339,116) were consistent with that of ECD calculation, thereby validating the accuracy and reliability of the ECD calculations.

**Fig. 4:**
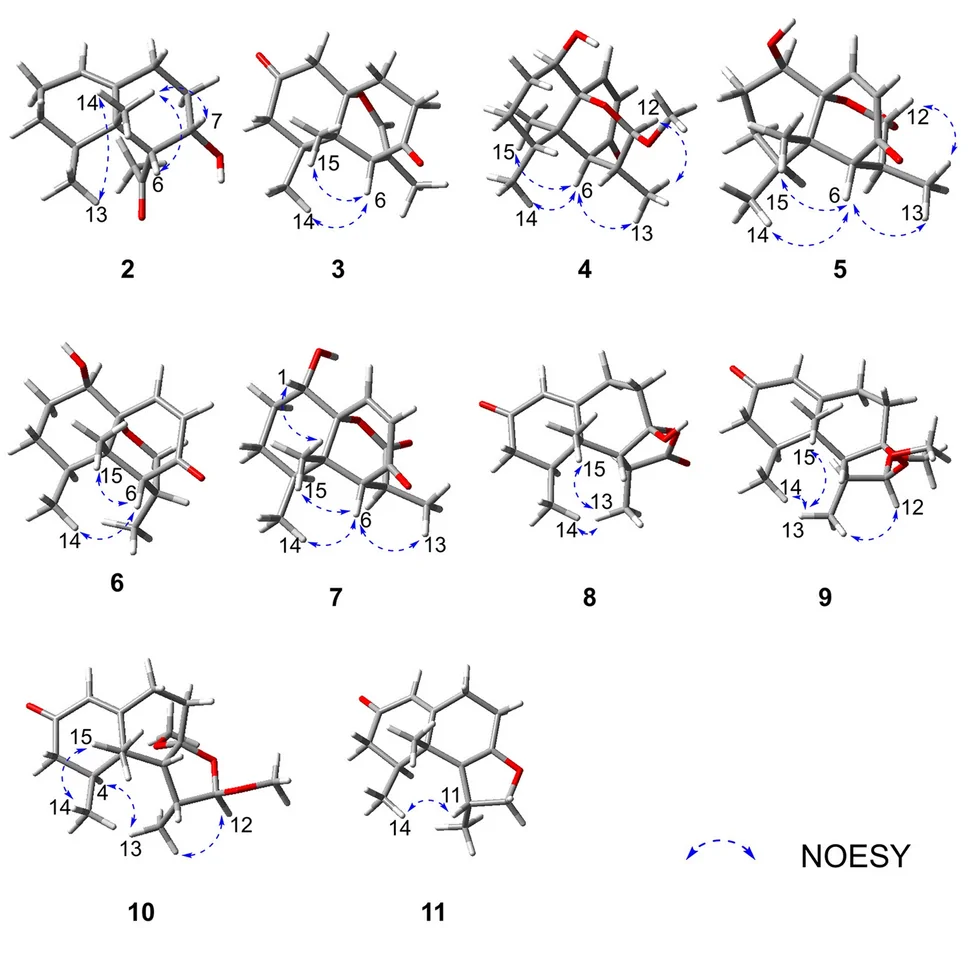
Key NOESY correlations of paralemnanoids B–K (**2**–**11**)

Paralemnanoid C (**3**) was isolated as colorless crystals. Its molecular formula was determined to be C_15_H_20_O_3_ by HRESIMS at *m/z* 251.1646 [M + H]^+^ (calcd. for 251.1642), requiring six degrees of unsaturation. The ^1^H and ^13^C NMR data (Table 1), in association with the HMBC correlations established a nornardosinane-type sesquiterpenoid as shown (Fig. 2), which is similar to the known compound tricyclic diketone 4 (Jurek and Scheuer 2004), except for **3** possessed the Δ^11,12^ double bond. In the NOESY spectrum of **3**, H-6 displayed correlations with H_3_-14 and H_3_-15, suggesting that these groups were coplanar (Fig. 4). The absolute configuration of **3** was confirmed to be 4*S*, 5*S*, 6*R*, 10*S* by data collection of single-crystal X-ray diffraction with Cu Kα radiation (Fig. 3, CCDC: 2,339,115).

Paralemnanoids D and E (**4** and **5**) were obtained as colorless oils, with molecular formula of C_16_H_24_O_4_ and C_15_H_22_O_4_, respectively. The 1D and 2D NMR spectra of **4** and **5** showed highly similar characteristic signals as nardosinane-type structures (Table 1, Fig. 2). They shared a 6/6/6 tricyclic framework with a hydroxy positioned at C-1, an α, β-unsaturated keto group spanning from C-7 to C-9, and a bridge connecting C-10 and C-12 through an oxygen atom. According to the HRESIMS, the molecular ion peak of compound **4** at *m/z* 281.1751 [M + H]^+^, 14 mass more than that of compound **5**. Based on the ^1^H and ^13^C NMR, ^1^H − ^1^H COSY, HSQC, and HMBC data (Fig. 2), **5** was determined to be a nardosinane-type sesquiterpenoid with an α,β-unsaturated keto group spanning C-7 to C-9 (C-7: *δ*_C_ 201.1; C-8: *δ*_C_ 132.9; C-9: *δ*_C_ 145.1), an oxo bridge connecting C-10 (*δ*_C_ 76.4) and C-12 (*δ*_C_ 96.3), one hydroxy group at C-12, and another one hydroxy group at the C-1 position (*δ*_C_ 69.4). Compared to **5**, the planar structure of **4** was determined as shown that a methoxyl group replaced a hydroxyl group at C-12.

**Fig. 5:**
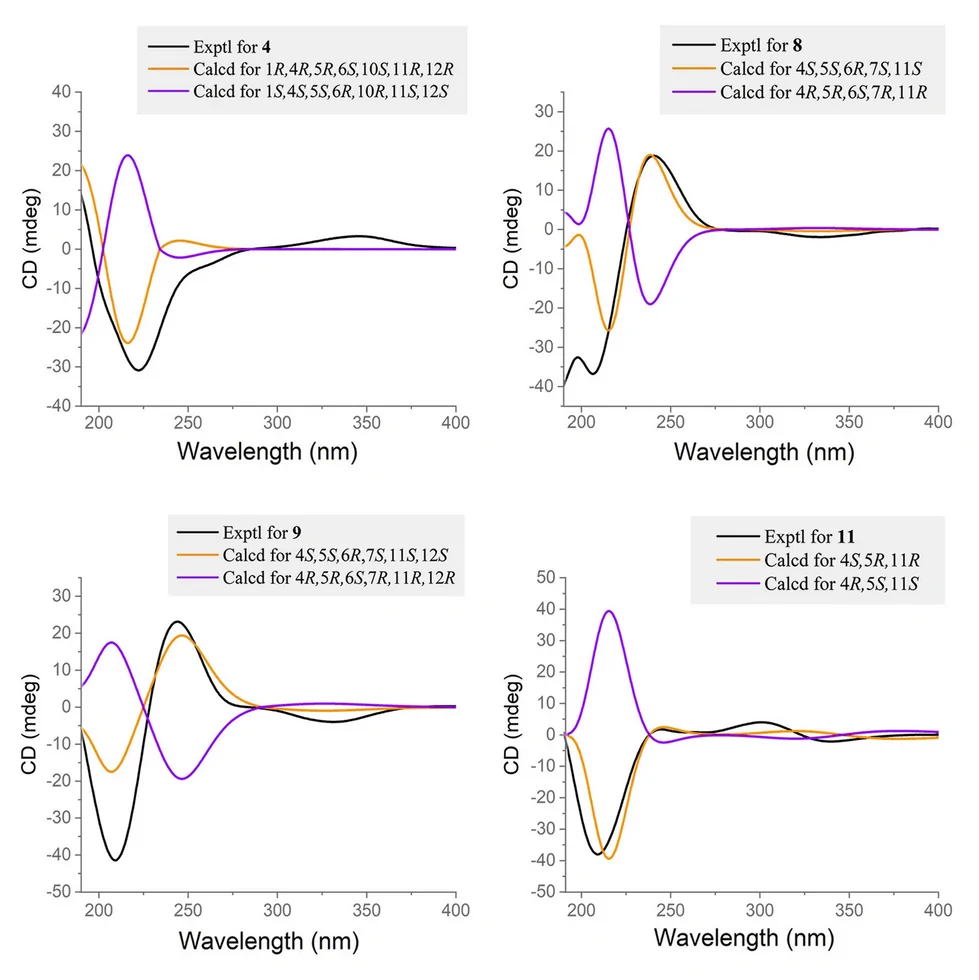
Experimental and calculated ECD spectra of **4**, **8**, **9**, and **11**

In the NOESY spectrum of **4**, the correlations between H-6 and H_3_-13/H_3_-14/H_3_-15, between H-12 and H_3_-13, revealed that these groups were coplanar (Fig. 4). Thus, the relative configuration of **4** was determined as 4R*, 5R*, 6S*, 10S*, 11R*, 12R*. To further assign the relative configuration of **4**, DP4 + analysis and ECD calculation were used. The simplified structures (1*S*, 4*R*, 5*R*, 6*S*, 10*S*, 11*R*, 12*R*)-**4a** and (1*R*, 4*R*, 5*R*, 6*S*, 10*S*, 11*R*, 12*R*)-**4b** were used for the DP4 + probability analysis. As a result, the candidate structure **4a** gave a best match between experimental and calculated data with 100% probability (Supplementary Figs. S13a and S13b). ECD calculations performed on **4a** showed that the calculated ECD curve of the enantiomer 1*S*, 4*R*, 5*R*, 6*S*, 10*S*, 11*R*, 12*R* matched well to the experimental ECD data (Fig. 5). Notably, compound **4** possessed several chiral centers, however, its absolute configuration is nearly opposite of the classical nardosinane-type sesquiterpene (excluding C-1 position), which has never been reported before, enriched the diversity of structure in this type of sesquiterpene. In the NOESY spectra of **5**, H-6 showed correlation with H_3_-13, H_3_-14 and H_3_-15, together with H-12 correlated with H_3_-13, indicated that H-6/H-12/H_3_-13/H_3_-14/H_3_-15 had the same orientation (Fig. 4). Lacking of the correlation from H-1 to neither H-4 nor H_3_-14, H_3_-15 in the NOESY spectra, led to the orientation of H-1 could not be determined. Ultimately, single crystals suitable for X-ray crystallographic analysis were obtained from chloroform solution. The result revealed that the absolute configuration of **5** was 1*R*, 4*S*, 5*S*, 6*R*, 10*R*, 11*S*, 12*S* (Fig. 3, CCDC: 2,410,572).

Paralemnanoid F (**6**) was isolated as light yellow oil, and it gave the HRESIMS ion peak at *m/z* 251.1646 [M + H]^+^ (calcd. for 251.1642), which corresponded to a molecular formula of C_15_H_22_O_3_. The planar structure of **6** was determined to be very similar to that of compound **5** on the basis of 1D and 2D NMR data, in which a hydroxy group in 5 was replaced by a proton in 6 at C-12 (Fig. 2). The relative configuration of **6** was established on the basis of NOESY data. In the NOESY spectra, H-6 showed correlations with H_3_-14/H_3_-15, indicated that H-6/H_3_-14/H_3_-15 had the same orientation (Fig. 4). The relative configurations of C-1 and C-11 in** 6** were established by the DP4 + analysis. The DP4 + analysis predicted the relative configuration **6b** (1*R*, 4*S*, 5*S*, 6*R*, 10*R*, 11*R*) of **6** to match the experimental results with 100% probability (Supplementary Figs. S14a and S14b). Ultimately, the absolute configuration of compound **6** was determined by ECD calculation as 1*R*, 4*S*, 5*S*, 6*R*, 10*R*, 11*R* (Supplementary Fig. S14c).

Paralemnanoid G (**7**) was isolated as colorless oil, with molecular formula of C_15_H_20_O_4_ and determined by HRESIMS at *m/z* 265.1433 [M + H]^+^ (calcd. for 265.1344). The similarity of 1D NMR spectrum between **7** and **5** indicated that **7** possessed the same 6/6/6 tricyclic sesquiterpene skeleton as **5**, except for a ketocarbonyl group replaced the hydroxyl at C-12, supported by the ^13^C chemical shift of the 10,12-olide moiety (*δ*_C-10_: 83.8; *δ*_C-12_: 172.9). The relative configuration of **7** was established by the NOESY experiment. In the NOESY spectrum, H_3_-15 correlated with H-1/H-11, and H-6 correlated with the H_3_-13/H_3_-14/H_3_-15, suggesting that they were coplanar (Fig. 4). It was found that **7** possessed the same planar structure as that of the known compound clavukoellian E (Wang et al. 2019), despite significant differences existing in the 1D and 2D NMR spectra of them. By analyzing the NOESY experiment, we found that the orientation of the hydroxyl group at C-1 in the two compounds was opposite, which was attributed to the NMR difference between **7** and clavukoellian E. Since C-1 is remote from the chromophore in compound **7**, we compared their ECD spectra to confirm the absolute configuration of **7**. The ECD spectrum of **7** showed a negative Cotton effect at 226 nm (Δ*ε* =—33.1), while clavukoellian E also showed a negative Cotton effect at 229 nm (Δ*ε* =—15.1). Subsequently, in contrast to clavukoellian E (1*R*, 4*S*, 5*S*, 6*R*, 10*R*, 11*S*), absolute configuration of** 7** was 1*S*, 4*S*, 5*S*, 6*R*, 10*R*, 11*S*, which is verified by the result of the ECD calculation (Supplemantary Fig. S15).

Paralemnanoid H (**8**) was isolated as light yellow oil, with molecular formula of C_15_H_20_O_4_ and determined by HRESIMS at *m/z* 265.1439 [M + H]^+^ (calcd. for 265.1434), three spin systems from H-4 to H_3_-14, from H_2_-8 to H_2_-9, and from H_3_-13 to H-11 were observed in the ^1^H − ^1^H COSY spectrum, together with the HMBC correlations from H-1 to C-3, from H_2_-3 to C-2, from H_2_-8 to C-6/C-7, from H_2_-9 to C-1/C-10, from H_3_-13 to C-6/C-12, from H_3_-14 to C-3/C-5, from H_3_-15 to C-4/C-5/C-6/C-10, enabled us to establish a lactonized 6/6/5 nardosinane-type planar structure of **8** (Fig. 2).

The determination of the relative configuration of **8** was elucidated by analysis of NOESY data. In the NOESY experiment, the correlations between H_3_-15 and H_3_-13/ H_3_-14 disclosed that these groups had the same orientation. To elucidate the relative configurations of C-7 and C-8 of **8**, DP4 + probability analysis was applied. The four possible structures (4*S*, 5*S*, 6*R*, 7*S*, 11*S*)-**8a,** (4*S*, 5*S*, 6*S*, 7*R*, 11*S*)-**8b**, (4*S*, 5*S*, 6*R*, 7*R*, 11*S*)-**8c**, and (4*S*, 5*S*, 6*S*, 7*S*, 11*S*)-**8d** were used for DP4 + probability analysis, the result turned out to give the best match for the isomer **8a** (4*S*, 5*S*, 6*R*, 7*S*, 11*S*) with 100% probability (Supplementary Figs. S16a and S16b). ECD calculation finally used to determine the absolute configuration of **8** as 4*S*, 5*S*, 6*R*, 7*S*, 11*S* (Fig. 5).

Paralemnanoid I (**9**) was obtained as colorless oil, gave the molecular formula C_17_H_26_O_4_ on the basis of the HRESIMS ion peak at *m/z* 295.1909 [M + H]^+^ (calcd. for 295.1904), with four degrees of unsaturation. The planarity of 9 was established as a nardosinane-type sesquiterpenoid by analysis of NMR data (Fig. 2). In the NOESY spectra, correlations between H-12 and H_3_-13, between H_3_-13 and H_3_-14/H_3_-15, disclosed the α-orientation of H-12, H_3_-13, H_3_-14 and H_3_-15 (Fig. 4). Overlapping ^1^H signals of H-7 and H_2_-11 led to several assignment ambiguities in the NOESY spectrum, thereby hampering the deduction of the relative configuration of C-6 and C-7. Therefore, DP4 + analysis was used to assign the relative configuration of **9** further. The structure (4*S*, 5*S*, 6*S*, 7*R*, 11*S*, 12*S*)**-9a**, (4*S*, 5*S*, 6*R*, 7*S*, 11*S*, 12*S*)-**9b**, (4*S*, 5*S*, 6*S*, 7*S*, 11*S*, 12*S*)-**9c** and (4*S*, 5*S*, 6*R*, 7*R*, 11*S*, 12*S*)-**9d** were used for DP4 + probability analysis. The results showed that **9b** was identified as the correct one by DP4 + analysis with 100% probability (Supplementary Figs. S17a and S17b). Then, ECD calculation finally determined the absolute configuration of **9** as 4*S*, 5*S*, 6*R*, 7*S*, 11*S*, 12*S* (Fig. 5).

Paralemnanoid J (**10**) was also isolated as colorless oil, with the same molecular formula of C_17_H_26_O_4_ by the HRESIMS ion peak at *m/z* 295.1907 [M + H]^+^ (calcd. for 295.1904). By analyzing ^1^H − ^1^H COSY and HMBC, **10** and **9** were discovered to have the same planar structure (Fig. 2). In the NOESY experiment of **10**, The NOESY cross-peak of H_3_-13 to H-4/H-12, H_3_-14 to H_3_-15, suggested that H-12 had the opposite orientation to H-11, H_3_-14 and H_3_-15 (Fig. 4). Subsequently, DP4 + analysis was used to determine the relative configuration of **10** at C-6 and C-7. The structure (4*S*, 5*S*, 6*S*, 7*R*, 11*R*, 12*R*)-**10a**, (4*S*, 5*S*, 6*R*, 7*S*, 11*R*, 12*R*)-**10b**, (4*S*, 5*S*, 6*S*, 7*S*, 11*R*, 12*R*)-**10c** and (4*S*, 5*S*, 6*R*, 7*R*, 11*R*, 12*R*)-**10d** were used for DP4 + probability analysis. The result showed that **10d** was identified as the correct one by DP4 + analysis with 93.31% probability (Supplementary Figs. S18a and S18b). Then, ECD calculations performed on **10d** showed that the calculated ECD curve of the enantiomer 4*S*, 5*S*, 6*R*, 7*R*, 11*R*, 12*R* matched well to the experimental ECD data (Supplementary Fig. S18c).

Paralemnanoid K (**11**) was isolated as light yellow oil, with molecular formula C_15_H_20_O_2_ and determined by HRESIMS at *m/z* 223.1538 [M + H]^+^ (calcd. for 223.1536), requiring six degrees of unsaturation. In the NMR spectra of **11**, COSY consecutive correlations from H-11 to H_3_-13 and H_2_-12 built one spin system. Together with HMBC spectra showed correlations from H-1 to C-3/C-5/C-9, from H-3 to C-2, from H_2_-8 to C-7/C-6, from H_2_-9 to C-8/C-10, from H_3_-13 to C-5/C-6, from H_3_-14 to C-3/C-4/C-5, from H_3_-15 to C-4/C-5/C-6/C-10, established an etherified 6/6/5 nardosinane-type structure (Fig. 2).

The relative configuration of **11** was deduced by the NOESY configuration from H-11 to H_3_-14 indicated that H_3_-14 must have the same orientation as H-11 (Fig. 4). Lacking of the correlated signals from H_3_-15 to neither H_3_-13 nor H_3_-14, two possible structures (4*S*, 5*S*, 11*R*)-**11a** and (4*S*, 5*R*, 11*R*)-**11b** were used for DP4 + probability analysis. The DP4 + probability analysis suggested that **11** was likely the configuration of **11b** (Supplementary Figs. S19a and S19b). ECD calculation was performed to assign the absolute configuration of **11** as 4*S*, 5*R*, 11*R* (Fig. 5).

**Fig. 6:**
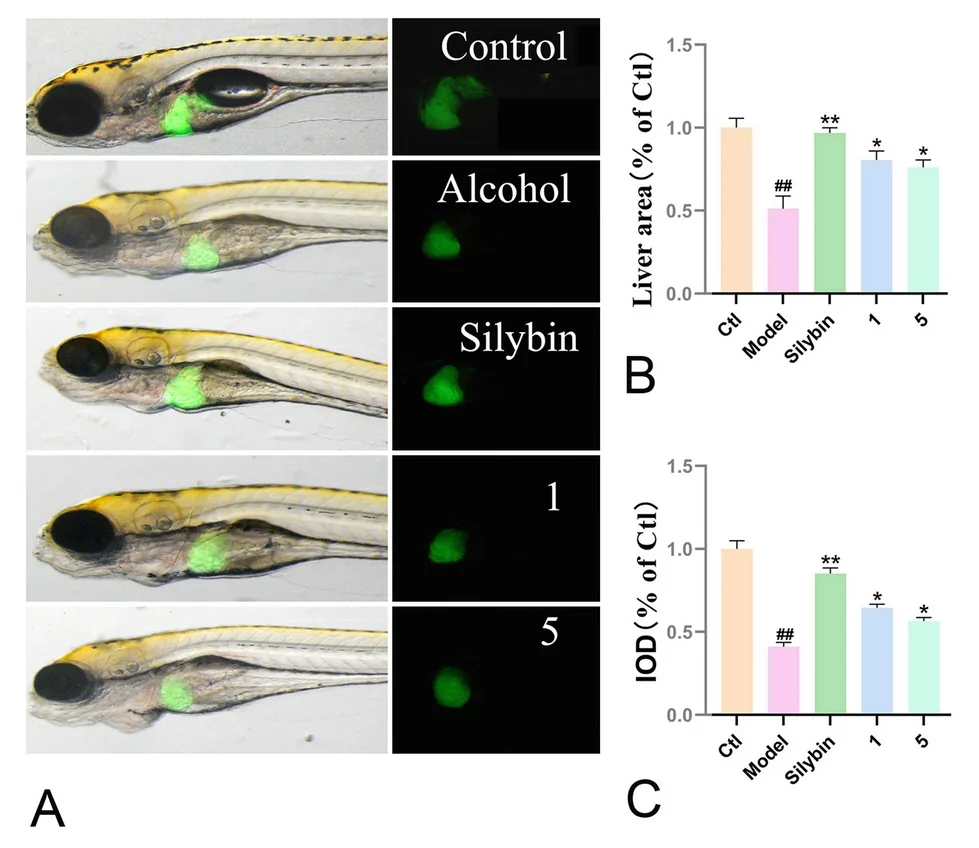
**A.** In vivo visualization of liver morphology of zebrafish larvae treated with alcohol and compounds **1** and **5**, respectively. Using silybin as the positive control. **B.** Quantitative analysis of the size of the liver in zebrafish treated with alcohol, silybin, and **1**, **5**, respectively. **C**. Fluorescence intensity of the liver (IOD, Integrated Optical Density) in zebrafish treated with alcohol, silybin, and **1**, **5**, respectively. Data represented as mean ± standard deviation (SD). ^##^*P* < 0.01 compared to the control group; *P < 0.1 and **P < 0.01 compared to the alcohol-induced group

Liver diseases represent a significant global health challenge (Liu et al. 2021), underscoring the need to identify lead compounds with hepatoprotective properties. Hepatoprotective activity refers to the ability of a compound to protect liver structure and function in injury models induced by either alcohol or non-alcoholic agents. We investigated the hepatoprotective effects of compounds **1** and **5** in transgenic fluorescent zebrafish models, where liver injury was induced by alcohol (Chen et al. 2023; Liu et al. 2021; Pandey et al. 2023). Quantitative analysis of liver fluorescence intensity and area provided a means to assess the protective effect of the compounds on both liver size and function. The results indicated that compounds **1** and **5** were able to partially restore liver function following injury, demonstrating moderate hepatoprotective activity comparable to the positive control at approximately 20 μmol/L (Fig. 6). Collectively, these experiments demonstrated that compounds **1** and **5** exhibited promising hepatoprotective activity, suggesting their potential as lead compounds for drug development.

## Discussion

### The biosynthetic pathway of the new skeleton 1

**Fig. 7:**
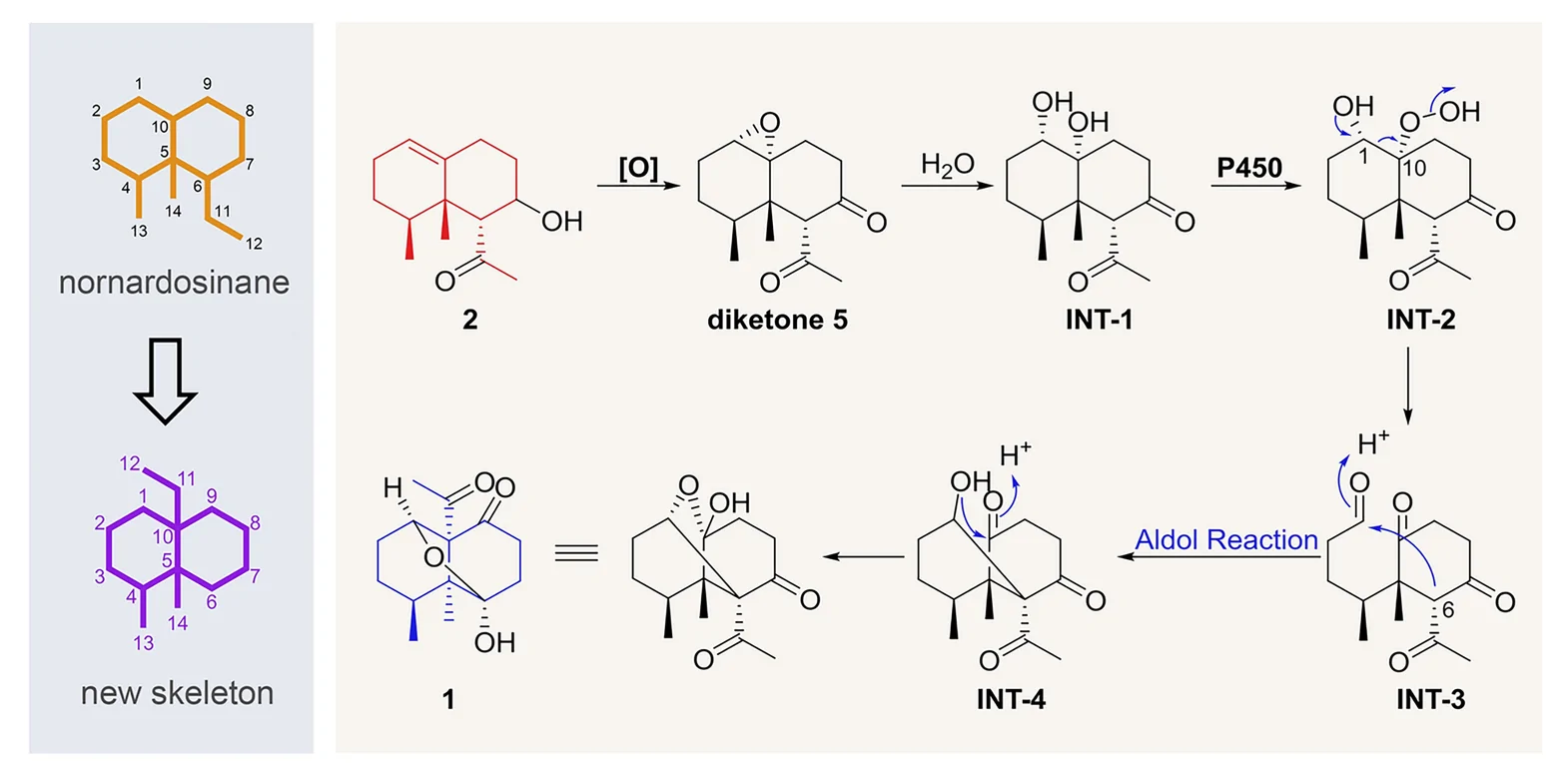
Proposed biosynthesis pathway of paralemnanoid A (**1**)

As a nardosinane-type norsesquiterpenoid featuring a novel carbon skeleton, compound** 1** has garnered significant attention due to its distinctive biosynthetic pathway. Compared to typical nardosinane-type norsesquiterpenoids, the structural novelty of paralemnanoid A (**1**) lies predominantly in the acetyl group position, the configuration inversion at C-5, and the rearrangement of the oxygen bridge attachment site. Given that compound **1** contains multiple oxidized functional groups (e.g. a hydroxyl group at C-6), its carbon scaffold formation during the terpene cyclase stage would impose stringent demands on the promiscuity of enzymes responsible for its structural modifications. Thus, the acetyl group migration likely occurs subsequent to the oxidative modification stage. This observation implies the involvement of a novel oxidative modification enzyme facilitating the reorganization of C–C bonds. Accordingly, we propose that paralemnanoid B (**2**) serves as the biosynthetic precursor of paralemnanoid A (**1**). Initially, a ring-oxidizing enzyme modifies the C1-C2 position of compound **2**, while an oxidizing enzyme catalyzes dehydrogenation at C-7, yielding the known intermediate diketone 5 (Izac et al. 1982). Subsequently, the epoxide group in diketone 5 undergoes spontaneous ring-opening, leading to the formation of intermediate **INT-1**. A novel P450 enzyme then mediates the cleavage of the C1-C10 bond in **INT-1**, resulting in the formation of **INT-3** through a C-10 hydroperoxide intermediate. This is followed by the formation of the C1-C6 bond via an intramolecular aldol condensation. Finally, the C-1 hydroxyl group attacks the C-10 carbonyl group, culminating in the formation of a novel oxygen bridge structure and ultimately yielding compound **1** (Fig. 7). This biosynthetic pathway elucidates the inversion of the C-14 orientation and the formation of the uncommon oxygen bridge, providing fresh insights into the biosynthetic logic underlying this type of norsesquiterpenoid skeleton.

### Expectation

With the ongoing exploration of cytochrome P450 enzymes from microbial, human, and other sources, novel P450 enzymes have been increasingly characterized, contributing significantly to enzymatic synthesis and drug development. However, in recent years, the rising costs of discovering new functional enzymes have constrained the pace of advancement in this field. Natural products derived from soft corals represent a valuable component of marine biological resources. Historically, research into their biosynthetic pathways, and the discovery of novel enzymes, has been hindered by the high cost of genome sequencing technologies, resulting in slow progress. Nevertheless, with the substantial decline in sequencing costs and advances in synthetic and combinatorial biology techniques, has propelled this area into a rapidly growing and increasingly prominent research field. This motivates us to conduct genomic mining in soft corals and perform bioinformatics analyses of genes associated with their secondary metabolites, aiming to discover novel enzymes and elucidate the biosynthetic pathways of coral-derived natural products exhibiting potent biological activity and unique structural frameworks through molecular biology approaches.

## Conclusions

In summary, this study on the soft coral *Paralemnalia* sp. identified a series of nardosinane-type (nor)sesquiterpenoids, including a novel carbon-skeleton nardosinane-type norsesquiterpenoid (paralemnanoid A, **1**), a new norsesquiterpenoid (paralemnanoid B, **2**), and nine sesquiterpenoids (paralemnanoids C − K, **3** − **11**) with diverse oxidation sites. The determination of the relative and absolute configurations of these compounds was achieved through NOESY experiments combined with DP4 + probability analysis, experimental and calculated ECD spectra, and single-crystal X-ray diffraction. Notably, the absolute configuration of compound **4**, reported here for the first time, deviates significantly from typical nardosinanes, highlighting the structural diversity of these sesquiterpenoids. Furthermore, we elucidated the biosynthetic pathway of the novel skeleton paralemnanoid A (**1**), which predominantly involves post-modification enzyme-mediated C − C bond cleavage, followed by aldol condensation-initiated C − C bond reformation. The intricate rearrangement of terpene skeletons typically occurs during the terpene cyclase-catalyzed ring-closing reactions, while post-modification enzyme-mediated skeleton rearrangement is rarely observed. This observation provides valuable insights into the biosynthetic mechanisms underlying the structural diversity of terpenoids. Furthermore, the alcohol-induced zebrafish liver injury model demonstrated that compounds **1** and **5** exhibited moderate hepatoprotective activity at 20 μmol/L, highlighting their potential as lead compounds. Notably, compound **1** warrants further investigation into its structure–activity relationship and the identification of active derivatives.

In conclusion, our ongoing investigation of this soft coral has expanded the diversity of sesquiterpenoid skeletons and uncovered novel compounds with hepatoprotective properties, warranting further investigation. Furthermore, the distinctive ecological and living environments of soft corals give rise to biosynthetic pathways that are markedly different from those of terrestrial organisms, potentially yielding greater chemical diversity and enhanced biological activity, with promising prospects for future applications.

## Supplementary Information

Below is the link to the electronic supplementary material.Supplementary file1 (DOCX 37869 KB)

## Data Availability

Data will be made available on request.
