# Supplementary material for: Paralemnanoids A–K, new norsesquiterpenoids and sesquiterpenoids with hepatoprotective activity from the soft coral Paralemnalia sp
Source: Mar Life Sci Technol. 2026 Mar 30;8(3):869–79. doi: 10.1007/s42995-025-00339-0 (PMC13525153; doi:10.1007/s42995-025-00339-0)
Supplement: Supplementary file 1 [file 42995_2025_339_MOESM1_ESM.docx]

*Supporting Information*

**Paralemnanoids A-K, New Norsesquiterpenoids and Sesquiterpenoids with Hepatoprotective Activity from the Soft Coral *Paralemnalia* sp.**

Yanfang Pei^1,2^ · Yuan Zong^3^ · Jixiang Xu^4^ · Huiyue Hou^1,2^ · Hengyi Xu^1,2^ · Yun Chen^1,2^ · Tianyun Jin^5^ ·Pinglin Li^*1,2^

^1^ Key Laboratory of Marine Drugs, Chinese Ministry of Education, School of Medicine and Pharmacy, Ocean University of China, Qingdao 266003, China

^2^ Laboratory for Marine Drugs and Bioproducts, Qingdao Marine Science and Technology Center, Qingdao, 266237, China

^3^ Shandong Academy of Chinese Medicine, Jinan 250014, China

^4^ State Key Laboratory of Bioactive Substances and Functions of Natural Medicines, Institute of Medicinal Biotechnology, Chinese Academy of Medical Sciences & Peking Union Medical College, Beijing 100050, China

^5^Center for Marine Biotechnology and Biomedicine, Scripps Institution of Oceanography, University of California San Diego, La Jolla, CA 92093-0204, USA

*E-mail: [lipinglin@ouc.edu.cn](mailto:17863087009@163.com；lipinglin@ouc.edu.cn)

**CONTENTS**

[**Table S1.** X-ray diffraction analysis of paralemnanoid A (**1**).	6](#_Toc186120125)

[**Table S2.** X-ray diffraction analysis of paralemnanoid B (**2**).	7](#_Toc186120126)

[**Table S3.** X-ray diffraction analysis of paralemnanoid C (**3**).	8](#_Toc186120127)

[**Table S4.** X-ray diffraction analysis of paralemnanoid E (**5**)**.**	9](#_Toc186120128)

[**Figure S1a**. ^1^H NMR spectrum (500 MHz) of paralemnanoid A (**1**) in CDCl_3_	10](#_Toc186120129)

[**Figure S1b**. ^13^C NMR spectrum (126 MHz) of paralemnanoid A (**1**) in CDCl_3_	10](#_Toc186120130)

[**Figure S1c**. HSQC spectrum of paralemnanoid A (**1**) in CDCl_3_	11](#_Toc186120131)

[**Figure S1d**. HMBC spectrum of paralemnanoid A (**1**) in CDCl_3_	11](#_Toc186120132)

[**Figure S1e**. ^1^H–^1^H COSY spectrum of paralemnanoid A (**1**) in CDCl_3_	12](#_Toc186120133)

[**Figure S1f**. NOESY spectrum of paralemnanoid A (**1**) in CDCl_3_	12](#_Toc186120134)

[**Figure S1g**. HRESIMS (positive mode) spectrum of paralemnanoid A (**1**)	13](#_Toc186120135)

[**Figure S2a**. ^1^H NMR spectrum (500 MHz) of paralemnanoid B (**2**) in CDCl_3_	13](#_Toc186120136)

[**Figure S2b**. ^13^C NMR spectrum (101 MHz) of paralemnanoid B (**2**) in CDCl_3_	14](#_Toc186120137)

[**Figure S2c**. HSQC spectrum of paralemnanoid B (**2**) in CDCl_3_	14](#_Toc186120138)

[**Figure S2d**. HMBC spectrum of paralemnanoid B (**2**) in CDCl_3_	15](#_Toc186120139)

[**Figure S2e**. ^1^H–^1^H COSY spectrum of paralemnanoid B (**2**) in CDCl_3_	15](#_Toc186120140)

[**Figure S2f**. NOESY spectrum of paralemnanoid B (**2**) in CDCl_3_	16](#_Toc186120141)

[**Figure S2g**. HRESIMS (positive mode) spectrum of paralemnanoid B (**2**)	16](#_Toc186120142)

[**Figure S2h**. ECD and UV spectra of paralemnanoid B (**2**)	17](#_Toc186120143)

[**Figure S3a**. ^1^H NMR spectrum (400 MHz) of paralemnanoid C (**3**) in CDCl_3_	17](#_Toc186120144)

[**Figure S3b**. ^13^C NMR spectrum (101MHz) of paralemnanoid C (**3**) in CDCl_3_	18](#_Toc186120145)

[**Figure S3c.** HSQC spectrum (500 MHz) of Paralemnanoid C (**3**) in CDCl_3_	18](#_Toc186120146)

[**Figure S3d**. HMBC spectrum of paralemnanoid C (**3**) in CDCl_3_	19](#_Toc186120147)

[**Figure S3e**. ^1^H–^1^H COSY spectrum of paralemnanoid C (**3**) in CDCl_3_	19](#_Toc186120148)

[**Figure S3f**. NOESY spectrum of paralemnanoid C (**3**) in CDCl_3_	20](#_Toc186120149)

[**Figure S3g**. HRESIMS (positive mode) spectrum of paralemnanoid C (**3**)	20](#_Toc186120150)

[**Figure S4a**. ^1^H NMR spectrum (500 MHz) of paralemnanoid D (**4**) in CDCl_3_	21](#_Toc186120151)

[**Figure S4b**. ^13^C NMR spectrum (101 MHz) of paralemnanoid D (**4**) in CDCl_3_	21](#_Toc186120152)

[**Figure S4c**. HSQC spectrum of paralemnanoid D (**4**) in CDCl_3_	22](#_Toc186120153)

[**Figure S4d**. HMBC spectrum of paralemnanoid D (**4**) in CDCl_3_	22](#_Toc186120154)

[**Figure S4e**. ^1^H–^1^H COSY spectrum of paralemnanoid D (**4**) in CDCl_3_	23](#_Toc186120155)

[**Figure S4f**. NOESY spectrum of paralemnanoid D (**4**) in CDCl_3_	23](#_Toc186120156)

[**Figure S4g**. HRESIMS (positive mode) spectrum of paralemnanoid D (**4**) in CDCl_3_	24](#_Toc186120157)

[**Figure S4h**. ECD and UV spectra of paralemnanoid D (**4**)	24](#_Toc186120158)

[**Figure S5a.** 1H NMR spectrum (500 MHz) of paralemnanoid E (**5**) in CDCl3	25](#_Toc186120159)

[**Figure S5b**. ^13^C NMR spectrum (151 MHz) of paralemnanoid E (**5**) in CDCl_3_	25](#_Toc186120160)

[**Figure S5c**. HSQC spectrum of paralemnanoid E (**5**) in CDCl_3_	26](#_Toc186120161)

[**Figure S5d**. HMBC spectrum of paralemnanoid E (**5**) in CDCl_3_	26](#_Toc186120162)

[**Figure S5e**. ^1^H–^1^H COSY spectrum of paralemnanoid E (**5**) in CDCl_3_	27](#_Toc186120163)

[**Figure S5f**. NOESY spectrum of paralemnanoid E (**5**) in CDCl_3_	27](#_Toc186120164)

[**Figure S5g**. HRESIMS (positive mode) spectrum of paralemnanoid E (**5**) in CDCl_3_	28](#_Toc186120165)

[**Figure S6a**. ^1^H NMR spectrum (500 MHz) of paralemnanoid F (**6**) in CDCl_3_	29](#_Toc186120166)

[**Figure S6b**. ^13^C NMR spectrum (151 MHz) of paralemnanoid F (**6**) in CDCl_3_	29](#_Toc186120167)

[**Figure S6c**. HSQC spectrum of paralemnanoid F (**6**) in CDCl_3_	30](#_Toc186120168)

[**Figure S6d**. HMBC spectrum of paralemnanoid F (**6**) in CDCl_3_	30](#_Toc186120169)

[**Figure S6e**. ^1^H–^1^H COSY spectrum of paralemnanoid F (**6**) in CDCl_3_	31](#_Toc186120170)

[**Figure S6f**. NOESY spectrum of paralemnanoid F (**6**) in CDCl_3_	31](#_Toc186120171)

[**Figure S6g**. HRESIMS (positive mode) spectrum of paralemnanoid F (**6**)	32](#_Toc186120172)

[**Figure S6h**. ECD and UV spectra of paralemnanoid F (**6**)	32](#_Toc186120173)

[**Figure S7a**. ^1^H NMR spectrum (600 MHz) of paralemnanoid G (**7**) in CDCl_3_	33](#_Toc186120174)

[**Figure S7b**. ^13^C NMR spectrum (126 MHz) of paralemnanoid G (**7**) in CDCl_3_	33](#_Toc186120175)

[**Figure S7c**. HSQC spectrum of paralemnanoid G (**7**) in CDCl_3_	34](#_Toc186120176)

[**Figure S7d**. HMBC spectrum of paralemnanoid G (**7**) in CDCl_3_	34](#_Toc186120177)

[**Figure S7e**. ^1^H–^1^H COSY spectrum of paralemnanoid G (**7**) in CDCl_3_	35](#_Toc186120178)

[**Figure S7f**. NOESY spectrum of paralemnanoid G (**7**) in CDCl_3_	35](#_Toc186120179)

[**Figure S7g**. HRESIMS (positive mode) spectrum of paralemnanoid G (**7**)	36](#_Toc186120180)

[**Figure S7h**. ECD and UV spectra of paralemnanoid G (**7**)	36](#_Toc186120181)

[**Figure S8a**. ^1^H NMR spectrum (600 MHz) of paralemnanoid H (**8**) in CDCl_3_	37](#_Toc186120182)

[**Figure S8b**. ^13^C NMR spectrum (126 MHz) of paralemnanoid H (**8**) in CDCl_3_	37](#_Toc186120183)

[**Figure S8c**. HSQC spectrum of paralemnanoid H (**8**) in CDCl_3_	38](#_Toc186120184)

[**Figure S8d**. HMBC spectrum of paralemnanoid H (**8**) in CDCl_3_	38](#_Toc186120185)

[**Figure S8e**. ^1^H–^1^H COSY spectrum of paralemnanoid H (**8**) in CDCl_3_	39](#_Toc186120186)

[**Figure S8f**. NOESY spectrum of paralemnanoid H (**8**) in CDCl_3_	39](#_Toc186120187)

[**Figure S8g**. HRESIMS (positive mode) spectrum of paralemnanoid H (**8**)	40](#_Toc186120188)

[**Figure S8h**. ECD and UV spectra of paralemnanoid H (**8**)	40](#_Toc186120189)

[**Figure S9a**. ^1^H NMR spectrum (500 MHz) of paralemnanoid I (**9**) in CDCl_3_	41](#_Toc186120190)

[**Figure S9b**. ^13^C NMR spectrum (126 MHz) of paralemnanoid I (**9**) in CDCl_3_	41](#_Toc186120191)

[**Figure S9c**. HSQC spectrum of paralemnanoid I (**9**) in CDCl_3_	42](#_Toc186120192)

[**Figure S9d**. HMBC spectrum of paralemnanoid I (**9**) in CDCl_3_	42](#_Toc186120193)

[**Figure S9e**. ^1^H–^1^H COSY spectrum of paralemnanoid I (**9**) in CDCl_3_	43](#_Toc186120194)

[**Figure S9f**. NOESY spectrum of paralemnanoid I (**9**) in CDCl_3_	43](#_Toc186120195)

[**Figure S9g**. HRESIMS (positive mode) spectrum of paralemnanoid I (**9**)	44](#_Toc186120196)

[**Figure S9h**. ECD and UV spectra of paralemnanoid I (**9**)	44](#_Toc186120197)

[**Figure S10a**. ^1^H NMR spectrum (500 MHz) of paralemnanoid J (**10**) in CDCl_3_	45](#_Toc186120198)

[**Figure S10b**. ^13^C NMR spectrum (126 MHz) of paralemnanoid J (**10**) in CDCl_3_	45](#_Toc186120199)

[**Figure S10c**. HSQC spectrum of paralemnanoid J (**10**) in CDCl_3_	46](#_Toc186120200)

[**Figure S10d**. HMBC spectrum of paralemnanoid J (**10**) in CDCl_3_	46](#_Toc186120201)

[**Figure S10e**. ^1^H–^1^H COSY spectrum of paralemnanoid J (**10**) in CDCl_3_	47](#_Toc186120202)

[**Figure S10f**. NOESY spectrum of paralemnanoid J (**10**) in CDCl_3_	47](#_Toc186120203)

[**Figure S10g**. HRESIMS (positive mode) spectrum of paralemnanoid J (**10**)	48](#_Toc186120204)

[**Figure S10h**. ECD and UV spectra of paralemnanoid J (**10**)	49](#_Toc186120205)

[**Figure S11a**. ^1^H NMR spectrum (500 MHz) of paralemnanoid K (**11**) in CDCl_3_	50](#_Toc186120206)

[**Figure S11b**. ^13^C NMR spectrum (151 MHz) of paralemnanoid K (**11**) in CDCl_3_	50](#_Toc186120207)

[**Figure S11c**. HSQC spectrum of paralemnanoid K (**11**) in CDCl_3_	51](#_Toc186120208)

[**Figure S11d**. HMBC spectrum of paralemnanoid K (**11**) in CDCl_3_	51](#_Toc186120209)

[**Figure S11e**. ^1^H–^1^H COSY spectrum of paralemnanoid K (**11**) in CDCl_3_	52](#_Toc186120210)

[**Figure S11f**. NOESY spectrum of paralemnanoid K (**11**) in CDCl_3_	52](#_Toc186120211)

[**Figure S11g**. HRESIMS (positive mode) spectrum of paralemnanoid K (**11**)	53](#_Toc186120212)

[**Figure S11h**. ECD and UV spectra of paralemnanoid K (**11**)	53](#_Toc186120213)

[**Calculation Details**	54](#_Toc186120214)

[**1. General Procedures**	54](#_Toc186120215)

[**2. The DP4+ analysis to deduce the relative configurations**	54](#_Toc186120216)

[**Table S5a**. Structures of isomers for paralemnanoid B (**2**)	55](#_Toc186120217)

[**Table S5b**. Optimized Z-Matrixes of paralemnanoid B (**2**) with simplified structures in the Gas Phase (Å) at B3LYP/6-31+G(d,p) level.	55](#_Toc186120218)

[**Figure S12a**. Experimental and calculated ECD spectra of paralemnanoid B (**2**).	56](#_Toc186120219)

[**Figure S13a**. Structures of isomers **4a** and **4b** for paralemnanoid D (**4**)	57](#_Toc186120220)

[**Figure S13b**. DP4+ results obtained using experimental data of paralemnanoid D (**4**) *versus* isomers **4a** and **4b**	57](#_Toc186120221)

[**Table S6a**. Stable conformers of paralemnanoid D (**4**).	58](#_Toc186120222)

[**Table S6b**.Optimized Z-Matrixes of paralemnanoid D (**4**) with simplified structures in the Gas Phase (Å) at B3LYP/6-31+G(d,p) level.	58](#_Toc186120223)

[**Figure S14a**. Structures of isomers **6a**, **6b**, **6c** and **6d** for paralemnanoid F (**6**)	61](#_Toc186120224)

[**Figure S14b**. DP4+ results obtained using experimental data of paralemnanoid F (**6**) *versus* isomers **6a**, **6b**, **6c** and **6d**	61](#_Toc186120225)

[**Table S7a**. Stable conformers of paralemnanoid F (**6**).	62](#_Toc186120226)

[**Table S7b**.Optimized Z-Matrixes of paralemnanoid F (**6**) with simplified structures in the Gas Phase (Å) at B3LYP/6-31+G(d,p) level.	62](#_Toc186120227)

[**Figure S14c**. Experimental ECD spectra of paralemnanoid F (**6**).	65](#_Toc186120228)

[**Figure S15**. Experimental ECD spectra of paralemnanoid G (**7**).	66](#_Toc186120229)

[**Figure S16a**. Structures of isomers **8a**, **8b, 8c** and **8d** for paralemnanoid H (**8**)	67](#_Toc186120230)

[**Figure S16b**. DP4+ results obtained using experimental data of paralemnanoid H (**8**) *versus* isomers **8a**, **8b**, **8c** and **8d**.	67](#_Toc186120231)

[**Table S8a**. Stable conformers of paralemnanoid H (**8**)	68](#_Toc186120232)

[**Table S8b**.Optimized Z-Matrixes of paralemnanoid H (**8**) with simplified structures in the Gas Phase (Å) at B3LYP/6-31+G(d,p) level.	68](#_Toc186120233)

[**Figure S17a**. Structures of isomers **9a**, **9b, 9c** and **9d** for paralemnanoid I (**9**)	73](#_Toc186120234)

[**Figure S17b**. DP4+ results obtained using experimental data of paralemnanoid I (**9**) *versus* isomers **9a**, **9b**, **9c** and **9d**.	73](#_Toc186120235)

[**Table S9a**. Stable conformers of paralemnanoid I (**9**)	74](#_Toc186120236)

[**Table S9b**.Optimized Z-Matrixes of paralemnanoid I (**9**) with simplified structures in the Gas Phase (Å) at B3LYP/6-31+G(d,p) level.	75](#_Toc186120237)

[**Figure S18a**. Structures of isomers **10a**, **10b, 10c** and **10d** for paralemnanoid J (**10**).	81](#_Toc186120238)

[**Figure S18b**. DP4+ results obtained using experimental data of paralemnanoid J (**10**) *versus* isomers **10a**, **10b**, **10c** and **10d**.	81](#_Toc186120239)

[**Table S10a**. Stable conformers of paralemnanoid J (**10**).	82](#_Toc186120240)

[**Table S10b**.Optimized Z-Matrixes of paralemnanoid J (**10**) with simplified structures in the Gas Phase (Å) at B3LYP/6-31+G(d,p) level.	82](#_Toc186120241)

[**Figure S18c**. Experimental and calculated ECD spectra of paralemnanoid J (**10**)	88](#_Toc186120242)

[**Figure S19a**. Structures of isomers **11a** and **11b** for paralemnanoid K (**11**).	89](#_Toc186120243)

[**Figure S19b**. DP4+ results obtained using experimental data of paralemnanoid K (**11**) *versus* isomers **11a** and **11b**.	89](#_Toc186120244)

[**Table S20a**. Stable conformers of paralemnanoid K (**11**).	90](#_Toc186120245)

[**Table S20b**.Optimized Z-Matrixes of paralemnanoid K (**11**) with simplified structures in the Gas Phase (Å) at B3LYP/6-31+G(d,p) level	90](#_Toc186120246)

| Empirical formula | C_14_H_20_O_4_ |
| --- | --- |
| Formula weight | 252.3 |
| Temperature/K | 150 |
| Crystal system | orthorhombic |
| Space group | P2_1_2_1_2_1_ |
| a/Å | 7.9477(2) |
| b/Å | 11.1531(2) |
| c/Å | 14.4527(3) |
| α/° | 90 |
| β/° | 90 |
| γ/° | 90 |
| Volume/Å^3^ | 1281.11(5) |
| Z | 4 |
| ρ_calc_g/cm^3^ | 1.308 |
| μ/mm^‑1^ | 0.777 |
| F(000) | 544 |
| Crystal size/mm^3^ | 0.2 × 0.15 × 0.1 |
| Radiation | CuKα (λ = 1.54178) |
| 2Θ range for data collection/° | 10.018 to 149.586 |
| Index ranges | -9 ≤ h ≤ 9, -13 ≤ k ≤ 13, -18 ≤ l ≤ 17 |
| Reflections collected | 12767 |
| Independent reflections | 2603 [R_int_ = 0.0259, R_sigma_ = 0.0212] |
| Data/restraints/parameters | 2603/0/167 |
| Goodness-of-fit on F^2^ | 1.101 |
| Final R indexes [I>=2σ (I)] | R_1_ = 0.0699, wR_2_ = 0.1500 |
| Final R indexes [all data] | R_1_ = 0.0754, wR_2_ = 0.1541 |
| Largest diff. peak/hole / e Å^-3^ | 0.55/-0.50 |
| Flack parameter | -0.02(5) |

**Table S1.** X-ray diffraction analysis of paralemnanoid A (**1**).

| Empirical formula | C_14_H_22_O_2_ |
| --- | --- |
| Formula weight | 222.31 |
| Temperature/K | 150 |
| Crystal system | monoclinic |
| Space group | P2_1_ |
| a/Å | 7.1133(12) |
| b/Å | 6.7661(9) |
| c/Å | 12.8843(16) |
| α/° | 90 |
| β/° | 90.28(2) |
| γ/° | 90 |
| Volume/Å^3^ | 620.10(15) |
| Z | 2 |
| ρ_calc_g/cm^3^ | 1.191 |
| μ/mm^‑1^ | 0.607 |
| F(000) | 244 |
| Crystal size/mm^3^ | 0.2 × 0.15 × 0.1 |
| Radiation | CuKα (λ = 1.54178) |
| 2Θ range for data collection/° | 6.86 to 149.462 |
| Index ranges | -8 ≤ h ≤ 8, -8 ≤ k ≤ 7, -16 ≤ l ≤ 16 |
| Reflections collected | 5884 |
| Independent reflections | 2206 [R_int_ = 0.1113, R_sigma_ = 0.1108] |
| Data/restraints/parameters | 2206/1/150 |
| Goodness-of-fit on F^2^ | 1.118 |
| Final R indexes [I>=2σ (I)] | R_1_ = 0.1085, wR_2_ = 0.2870 |
| Final R indexes [all data] | R_1_ = 0.1210, wR_2_ = 0.3130 |
| Largest diff. peak/hole / e Å^-3^ | 0.45/-0.29 |
| Flack parameter | 0.3(10) |

**Table S2.** X-ray diffraction analysis of paralemnanoid B (**2**).

| Empirical formula | C_15_H_20_O_3_ |
| --- | --- |
| Formula weight | 248.31 |
| Temperature/K | 150 |
| Crystal system | orthorhombic |
| Space group | P2_1_2_1_2_1_ |
| a/Å | 9.0045(2) |
| b/Å | 13.7640(3) |
| c/Å | 31.7354(6) |
| α/° | 90 |
| β/° | 90 |
| γ/° | 90 |
| Volume/Å^3^ | 3933.22(14) |
| Z | 12 |
| ρ_calc_g/cm^3^ | 1.258 |
| μ/mm^‑1^ | 0.694 |
| F(000) | 1608 |
| Crystal size/mm^3^ | 0.22 × 0.2 × 0.15 |
| Radiation | CuKα (λ = 1.54178) |
| 2Θ range for data collection/° | 5.57 to 144.41 |
| Index ranges | -11 ≤ h ≤ 8, -16 ≤ k ≤ 16, -38 ≤ l ≤ 39 |
| Reflections collected | 28004 |
| Independent reflections | 7692 [R_int_ = 0.0351, R_sigma_ = 0.0304] |
| Data/restraints/parameters | 7692/0/496 |
| Goodness-of-fit on F^2^ | 1.056 |
| Final R indexes [I>=2σ (I)] | R_1_ = 0.0656, wR_2_ = 0.1547 |
| Final R indexes [all data] | R_1_ = 0.0665, wR_2_ = 0.1563 |
| Largest diff. peak/hole / e Å^-3^ | 0.34/-0.28 |
| Flack parameter | -0.02(5) |

**Table S3.** X-ray diffraction analysis of paralemnanoid C (**3**).

| Empirical formula | C_15_H_22_O_4_ |
| --- | --- |
| Formula weight | 267.15 |
| Temperature/K | 150 |
| Crystal system | orthorhombic |
| Space group | P2_1_2_1_2_1_ |
| a/Å | 6.85530(10) |
| b/Å | 12.2767(2) |
| c/Å | 21.3380(4) |
| α/° | 90 |
| β/° | 90 |
| γ/° | 90 |
| Volume/Å^3^ | 1795.82(5) |
| Z | 4 |
| ρ_calc_g/cm^3^ | 1.427 |
| μ/mm^‑1^ | 4.764 |
| F(000) | 808 |
| Crystal size/mm^3^ | 0.2 × 0.15 × 0.1 |
| Radiation | CuKα (λ = 1.54178) |
| 2Θ range for data collection/° | 8.288 to 149.496 |
| Index ranges | -8 ≤ h ≤ 7, -15 ≤ k ≤ 15, -26 ≤ l ≤ 26 |
| Reflections collected | 16684 |
| Independent reflections | 3654 [R_int_ = 0.0373, R_sigma_ = 0.0361] |
| Data/restraints/parameters | 3654/0/213 |
| Goodness-of-fit on F^2^ | 1.127 |
| Final R indexes [I>=2σ (I)] | R_1_ = 0.0738, wR_2_ = 0.1765 |
| Final R indexes [all data] | R_1_ = 0.0745, wR_2_ = 0.1780 |
| Largest diff. peak/hole / e Å^-3^ | 0.85/-0.30 |
| Flack parameter | 0.022(5) |

**Table S4.** X-ray diffraction analysis of paralemnanoid E (**5**)**.**

**Figure S1a**. ^1^H NMR spectrum (500 MHz) of paralemnanoid A (**1**) in CDCl_3_

**Figure S1b**. ^13^C NMR spectrum (126 MHz) of paralemnanoid A (**1**) in CDCl_3_

**Figure S1c**. HSQC spectrum of paralemnanoid A (**1**) in CDCl_3_

 **Figure S1d**. HMBC spectrum of paralemnanoid A (**1**) in CDCl_3_

**Figure S1e**. ^1^H–^1^H COSY spectrum of paralemnanoid A (**1**) in CDCl_3_

 **Figure S1f**. NOESY spectrum of paralemnanoid A (**1**) in CDCl_3_

**Figure S1g**. HRESIMS (positive mode) spectrum of paralemnanoid A

**Figure S2a**. ^1^H NMR spectrum (500 MHz) of paralemnanoid B (**2**) in CDCl_3_

**Figure S2b**. ^13^C NMR spectrum (101 MHz) of paralemnanoid B (**2**) in CDCl_3_

 **Figure S2c**. HSQC spectrum of paralemnanoid B (**2**) in CDCl_3_

**Figure S2d**. HMBC spectrum of paralemnanoid B (**2**) in CDCl_3_

 **Figure S2e**. ^1^H–^1^H COSY spectrum of paralemnanoid B (**2**) in CDCl_3_

**Figure S2f**. NOESY spectrum of paralemnanoid B (**2**) in CDCl_3_

**Figure S2g**. HRESIMS (positive mode) spectrum of paralemnanoid B (**2**)

**Figure S2h**. ECD and UV spectra of paralemnanoid B (**2**)

**Figure S3a**. ^1^H NMR spectrum (400 MHz) of paralemnanoid C (**3**) in CDCl_3_

**Figure S3b**. ^13^C NMR spectrum (101MHz) of paralemnanoid C (**3**) in CDCl_3_

 **Figure S3c.** HSQC spectrum (500 MHz) of Paralemnanoid C (**3**) in CDCl_3_

**Figure S3d**. HMBC spectrum of paralemnanoid C (**3**) in CDCl_3_

 **Figure S3e**. ^1^H–^1^H COSY spectrum of paralemnanoid C (**3**) in CDCl_3_

**Figure S3f**. NOESY spectrum of paralemnanoid C (**3**) in CDCl_3_

**Figure S3g**. HRESIMS (positive mode) spectrum of paralemnanoid C (**3**)

**Figure S4a**. ^1^H NMR spectrum (500 MHz) of paralemnanoid D (**4**) in CDCl_3_

 **Figure S4b**. ^13^C NMR spectrum (101 MHz) of paralemnanoid D (**4**) in CDCl_3_

**Figure S4c**. HSQC spectrum of paralemnanoid D (**4**) in CDCl_3_

 **Figure S4d**. HMBC spectrum of paralemnanoid D (**4**) in CDCl_3_

**Figure S4e**. ^1^H–^1^H COSY spectrum of paralemnanoid D (**4**) in CDCl_3_

 **Figure S4f**. NOESY spectrum of paralemnanoid D (**4**) in CDCl_3_

**Figure S4g**. HRESIMS (positive mode) spectrum of paralemnanoid D (**4**) in CDCl_3_

**Figure S4h**. ECD and UV spectra of paralemnanoid D (**4**)

**Figure S5a.** 1H NMR spectrum (500 MHz) of paralemnanoid E (**5**) in CDCl_3_

**Figure S5b**. ^13^C NMR spectrum (151 MHz) of paralemnanoid E (**5**) in CDCl_3_

**Figure S5c**. HSQC spectrum of paralemnanoid E (**5**) in CDCl_3_

**Figure S5d**. HMBC spectrum of paralemnanoid E (**5**) in CDCl_3_

**Figure S5e**. ^1^H–^1^H COSY spectrum of paralemnanoid E (**5**) in CDCl_3_

 **Figure S5f**. NOESY spectrum of paralemnanoid E (**5**) in CDCl_3_

**Figure S5g**. HRESIMS (positive mode) spectrum of paralemnanoid E (**5**) in CDCl_3_

**Figure S5h**. ECD and UV spectra of Paralemnanoid E (**5**)

 **Figure S6a**. ^1^H NMR spectrum (500 MHz) of paralemnanoid F (**6**) in CDCl_3_

 **Figure S6b**. ^13^C NMR spectrum (151 MHz) of paralemnanoid F (**6**) in CDCl_3_

**Figure S6c**. HSQC spectrum of paralemnanoid F (**6**) in CDCl_3_

**Figure S6d**. HMBC spectrum of paralemnanoid F (**6**) in CDCl_3_

**Figure S6e**. ^1^H–^1^H COSY spectrum of paralemnanoid F (**6**) in CDCl_3_

**Figure S6f**. NOESY spectrum of paralemnanoid F (**6**) in CDCl_3_

**Figure S6g**. HRESIMS (positive mode) spectrum of paralemnanoid F (**6**)

**Figure S6h**. ECD and UV spectra of paralemnanoid F (**6**)

**Figure S7a**. ^1^H NMR spectrum (600 MHz) of paralemnanoid G (**7**) in CDCl_3_

**Figure S7b**. ^13^C NMR spectrum (126 MHz) of paralemnanoid G (**7**) in CDCl_3_

**Figure S7c**. HSQC spectrum of paralemnanoid G (**7**) in CDCl_3_

**Figure S7d**. HMBC spectrum of paralemnanoid G (**7**) in CDCl_3_

**Figure S7e**. ^1^H–^1^H COSY spectrum of paralemnanoid G (**7**) in CDCl_3_

**Figure S7f**. NOESY spectrum of paralemnanoid G (**7**) in CDCl_3_

**Figure S7g**. HRESIMS (positive mode) spectrum of paralemnanoid G (**7**)

**Figure S7h**. ECD and UV spectra of paralemnanoid G (**7**)

**Figure S8a**. ^1^H NMR spectrum (600 MHz) of paralemnanoid H (**8**) in CDCl_3_

**Figure S8b**. ^13^C NMR spectrum (126 MHz) of paralemnanoid H (**8**) in CDCl_3_

**Figure S8c**. HSQC spectrum of paralemnanoid H (**8**) in CDCl_3_

**Figure S8d**. HMBC spectrum of paralemnanoid H (**8**) in CDCl_3_

**Figure S8e**. ^1^H–^1^H COSY spectrum of paralemnanoid H (**8**) in CDCl

**Figure S8f**. NOESY spectrum of paralemnanoid H (**8**) in CDCl_3_

**Figure S8g**. HRESIMS (positive mode) spectrum of paralemnanoid H (**8**)

**Figure S8h**. ECD and UV spectra of paralemnanoid H (**8**)

**Figure S9a**. ^1^H NMR spectrum (500 MHz) of paralemnanoid I (**9**) in CDCl_3_

**Figure S9b**. ^13^C NMR spectrum (126 MHz) of paralemnanoid I (**9**) in CDCl_3_

**Figure S9c**. HSQC spectrum of paralemnanoid I (**9**) in CDCl_3_

**Figure S9d**. HMBC spectrum of paralemnanoid I (**9**) in CDCl_3_

**Figure S9e**. ^1^H–^1^H COSY spectrum of paralemnanoid I (**9**) in CDCl_3_

**Figure S9f**. NOESY spectrum of paralemnanoid I (**9**) in CDCl_3_

**Figure S9g**. HRESIMS (positive mode) spectrum of paralemnanoid I (**9**)

**Figure S9h**. ECD and UV spectra of paralemnanoid I (**9**)

**Figure S10a**. ^1^H NMR spectrum (500 MHz) of paralemnanoid J (**10**) in CDCl_3_

**Figure S10b**. ^13^C NMR spectrum (126 MHz) of paralemnanoid J (**10**) in CDCl_3_

**Figure S10c**. HSQC spectrum of paralemnanoid J (**10**) in CDCl_3_

**Figure S10d**. HMBC spectrum of paralemnanoid J (**10**) in CDCl_3_

**Figure S10e**. ^1^H–^1^H COSY spectrum of paralemnanoid J (**10**) in CDCl_3_

**Figure S10f**. NOESY spectrum of paralemnanoid J (**10**) in CDCl_3_

**Figure S10g**. HRESIMS (positive mode) spectrum of paralemnanoid J (**10**)

**Figure S10h**. ECD and UV spectra of paralemnanoid J (**10**)

**Figure S11a**. ^1^H NMR spectrum (500 MHz) of paralemnanoid K (**11**) in CDCl_3_

**Figure S11b**. ^13^C NMR spectrum (151 MHz) of paralemnanoid K (**11**) in CDCl_3_

**Figure S11c**. HSQC spectrum of paralemnanoid K (**11**) in CDCl_3_

**Figure S11d**. HMBC spectrum of paralemnanoid K (**11**) in CDCl_3_

**Figure S11e**. ^1^H–^1^H COSY spectrum of paralemnanoid K (**11**) in CDCl_3_

**Figure S11f**. NOESY spectrum of paralemnanoid K (**11**) in CDCl_3_

**Figure S11g**. HRESIMS (positive mode) spectrum of paralemnanoid K (**11**)

**Figure S11h**. ECD and UV spectra of paralemnanoid K (**11**)

**Calculation Details**

**1. General Procedures**

Conformational analyses were performed by the Spartan 14 and MOE software package using the MMFF minimization force field. The resulted conformers were further optimized with Gaussian 09 software by the density functional theory (DFT) calculations at the B3LYP/6-31+G(d,p) level in the gas phase. The optimized conformations, whose Boltzmann distributions of Gibbs free energies were more than 1.0 percent, were used for the NMR calculations using the GIAO method at the PCM/B3LYP/6-311+G(d,p) level, or the ECD using TD-DFT method with the basis set RB3LYP/DGDZVP.1-3ECD spectra of different conformers were simulated using a Gaussian function with a half-bandwidth ranging from 0.12to 0.4eV. The overall theoretical ECD spectra were then obtained according to the Boltzmann weighting and simulated experimental spectra by the SpecDis software.

**2. The DP4+ analysis to deduce the relative configurations**

In order to determine the relative configurations of paralemnanoids D, F-K, 1D NMR chemical shifts were calculated by Gaussian 09 program package. The gauge independent atomic orbital (GIAO) calculations of NMR shielding constants with spin-spin interactions were accomplished for all stable conformers using the DFT method at the B3LYP/6-311+G(d,p) level in the gas phase, using the PCM model and the optimal method for DP4+ analysis. The ^1^H and ^13^C NMR data obtained were directly statistically analyzed with experimental chemical shifts.

|  |  |
| --- | --- |
| **2a1** (96.9%) | **2a2** (3.1%) |

**Table S5a**. Structures of isomers for paralemnanoid B (**2**)

| **2a1** | | | | **2a2** | | | |
| --- | --- | --- | --- | --- | --- | --- | --- |
| C | 2.93688 | -0.65071 | -1.1379 | C | 2.87343 | -0.79942 | -1.14373 |
| H | 3.8846 | -1.20516 | -1.12326 | H | 3.7917 | -1.40084 | -1.11941 |
| H | 2.86892 | -0.21069 | -2.14472 | H | 2.82807 | -0.37974 | -2.16078 |
| C | 2.95027 | 0.45665 | -0.08143 | C | 2.94278 | 0.3291 | -0.11152 |
| H | 3.63563 | 1.25903 | -0.38019 | H | 3.65678 | 1.09549 | -0.43485 |
| H | 3.33755 | 0.05905 | 0.86562 | H | 3.32315 | -0.06363 | 0.83984 |
| C | 1.54372 | 1.04107 | 0.13024 | C | 1.56304 | 0.97296 | 0.10023 |
| H | 1.19557 | 1.35349 | -0.86139 | H | 1.21439 | 1.26934 | -0.89884 |
| C | 0.53632 | -0.05178 | 0.6312 | C | 0.52561 | -0.06823 | 0.64805 |
| C | 0.72309 | -1.35783 | -0.1552 | C | 0.64475 | -1.39648 | -0.11371 |
| C | 1.78838 | -1.59768 | -0.92984 | C | 1.68325 | -1.68979 | -0.91013 |
| H | 1.83782 | -2.54723 | -1.46222 | H | 1.68886 | -2.65444 | -1.41785 |
| C | -0.94425 | 0.46785 | 0.46289 | C | -0.93864 | 0.51279 | 0.51667 |
| H | -1.11971 | 1.23889 | 1.22122 | H | -1.059 | 1.24474 | 1.32383 |
| C | -1.98851 | -0.64298 | 0.72717 | C | -2.05144 | -0.55053 | 0.72117 |
| H | -1.89397 | -0.95339 | 1.7795 | H | -2.04243 | -0.82961 | 1.78715 |
| C | -1.76819 | -1.86193 | -0.16661 | C | -1.86023 | -1.82688 | -0.09304 |
| H | -2.50922 | -2.63479 | 0.08262 | H | -2.62998 | -2.54826 | 0.20181 |
| H | -1.92096 | -1.57225 | -1.20965 | H | -2.02243 | -1.61374 | -1.15331 |
| C | -0.34737 | -2.41625 | 0.02459 | C | -0.45666 | -2.41157 | 0.12077 |
| H | -0.16901 | -3.24056 | -0.67298 | H | -0.30627 | -3.27658 | -0.53308 |
| H | -0.27425 | -2.85054 | 1.03319 | H | -0.38872 | -2.79496 | 1.14928 |
| C | 1.61033 | 2.29467 | 1.01777 | C | 1.67176 | 2.26045 | 0.93156 |
| H | 2.20251 | 3.07073 | 0.52148 | H | 2.32849 | 2.97234 | 0.42057 |
| H | 2.08905 | 2.08772 | 1.98043 | H | 2.1037 | 2.07419 | 1.92014 |
| H | 0.62357 | 2.72091 | 1.22501 | H | 0.70336 | 2.75035 | 1.06214 |
| C | 0.77471 | -0.34328 | 2.13872 | C | 0.78157 | -0.34795 | 2.15467 |
| H | 1.82567 | -0.58242 | 2.32262 | H | 1.80787 | -0.68721 | 2.31943 |
| H | 0.18933 | -1.19465 | 2.49268 | H | 0.12019 | -1.12604 | 2.54279 |
| H | 0.50847 | 0.51663 | 2.76111 | H | 0.61767 | 0.54904 | 2.75815 |
| C | -1.24429 | 1.09388 | -0.91132 | C | -1.20719 | 1.36356 | -0.73994 |
| C | -1.91446 | 2.45268 | -0.90583 | C | -1.22131 | 0.7439 | -2.12409 |
| H | -2.089 | 2.78473 | -1.93043 | H | -1.05996 | 1.52913 | -2.86521 |
| H | -1.29694 | 3.18817 | -0.37711 | H | -2.21605 | 0.31477 | -2.2892 |
| H | -2.86405 | 2.37464 | -0.36607 | H | -0.48522 | -0.05486 | -2.23912 |
| O | -0.98426 | 0.51749 | -1.95314 | O | -1.44748 | 2.5518 | -0.59947 |
| O | -3.27407 | -0.04492 | 0.52933 | O | -3.33167 | -0.01969 | 0.37531 |
| H | -3.93473 | -0.74902 | 0.56638 | H | -3.4453 | 0.82232 | 0.83797 |

**Table S5b**. Optimized Z-Matrixes of paralemnanoid B (**2**) with simplified structures in the Gas Phase (Å) at B3LYP/6-31+G(d,p) level.

 **Figure S12a**. Experimental and calculated ECD spectra of paralemnanoid B (**2**).

**Figure S13a**. Structures of isomers **4a** and **4b** for paralemnanoid D (**4**)

**Figure S13b**. DP4+ results obtained using experimental data of paralemnanoid D (**4**) *versus* isomers **4a** and **4b**

|  |  |  |
| --- | --- | --- |
| **4a1** (66.7%) | **4a2** (33.1%) | **4b1** (99.4%) |

**Table S6a**. Stable conformers of paralemnanoid D (**4**).

| **4a1** | | | | **4b1** | | | |
| --- | --- | --- | --- | --- | --- | --- | --- |
| C | -0.54949 | -0.57175 | -0.88373 | C | -0.54748 | -0.56947 | -0.88801 |
| C | -2.05744 | -0.74703 | -1.11504 | C | -2.05755 | -0.77230 | -1.11751 |
| C | -2.86315 | 0.09955 | -0.12215 | C | -2.87158 | 0.07447 | -0.13897 |
| C | -2.41278 | -0.11371 | 1.32963 | C | -2.42073 | -0.11825 | 1.31625 |
| C | -0.90466 | 0.16742 | 1.53134 | C | -0.91443 | 0.16888 | 1.52237 |
| C | -0.05526 | -0.73594 | 0.56625 | C | -0.05615 | -0.73222 | 0.56234 |
| C | 1.42466 | -0.24836 | 0.21633 | C | 1.42462 | -0.23905 | 0.22416 |
| C | 2.26421 | -1.46673 | -0.16088 | C | 2.26634 | -1.45656 | -0.14179 |
| C | 1.49758 | -2.05719 | -1.35726 | C | 1.51122 | -2.04596 | -1.35400 |
| C | 0.28733 | -1.58008 | -1.73417 | C | 0.30659 | -1.56343 | -1.74066 |
| O | -2.32106 | -2.14505 | -0.96653 | O | -2.42568 | -2.13595 | -0.89492 |
| C | -0.51976 | 0.03845 | 3.01429 | C | -0.53411 | 0.04498 | 3.00702 |
| C | -0.08759 | -2.16941 | 1.15739 | C | -0.08901 | -2.16453 | 1.15597 |
| C | 1.60671 | 1.23964 | 0.50584 | C | 1.59806 | 1.25029 | 0.51371 |
| O | -0.30925 | 0.84799 | -1.26848 | O | -0.30502 | 0.84665 | -1.27642 |
| O | 3.34688 | -1.86463 | 0.21153 | O | 3.34576 | -1.85875 | 0.23258 |
| C | 0.59850 | 1.80939 | -0.59354 | C | 0.59292 | 1.81427 | -0.59365 |
| C | 3.01869 | 1.77692 | 0.26518 | C | 3.00842 | 1.79459 | 0.28032 |
| O | -0.12870 | 2.85967 | -0.04397 | O | -0.14215 | 2.85988 | -0.04867 |
| C | -0.80316 | 3.67986 | -0.99343 | C | -0.82114 | 3.67352 | -1.00121 |
| H | -2.28104 | -0.42402 | -2.14410 | H | -2.29356 | -0.47233 | -2.15083 |
| H | -2.74818 | 1.15257 | -0.39685 | H | -2.76463 | 1.12443 | -0.42587 |
| H | -3.92972 | -0.14057 | -0.23333 | H | -3.92740 | -0.19377 | -0.25237 |
| H | -2.66457 | -1.13258 | 1.64469 | H | -2.67492 | -1.13150 | 1.64542 |
| H | -2.97933 | 0.55711 | 1.98691 | H | -2.98912 | 0.56249 | 1.96182 |
| H | -0.77710 | 1.21355 | 1.25972 | H | -0.78885 | 1.21435 | 1.24836 |
| H | 1.38013 | -0.04874 | -0.84620 | H | 1.38852 | -0.03899 | -0.83885 |
| H | 1.99394 | -2.84930 | -1.91245 | H | 2.02833 | -2.81649 | -1.92091 |
| H | -0.20128 | -1.99182 | -2.61318 | H | -0.13839 | -1.93129 | -2.66561 |
| H | -3.26370 | -2.28446 | -1.12414 | H | -1.94252 | -2.69416 | -1.51787 |
| H | 0.55688 | 0.16211 | 3.17195 | H | 0.54189 | 0.16908 | 3.16987 |
| H | -1.02990 | 0.81118 | 3.59962 | H | -1.04689 | 0.81979 | 3.58704 |
| H | -0.80427 | -0.93076 | 3.43424 | H | -0.82176 | -0.92214 | 3.42967 |
| H | -1.08381 | -2.42008 | 1.52125 | H | -1.09344 | -2.42527 | 1.48798 |
| H | 0.18369 | -2.94592 | 0.44751 | H | 0.21857 | -2.94209 | 0.46100 |
| H | 0.60517 | -2.22955 | 2.00190 | H | 0.57852 | -2.21464 | 2.02097 |
| H | 1.27082 | 1.55855 | 1.49425 | H | 1.25408 | 1.56789 | 1.49947 |
| H | 1.19440 | 2.18151 | -1.44175 | H | 1.19267 | 2.18863 | -1.43820 |
| H | 3.02996 | 2.87119 | 0.30641 | H | 3.01305 | 2.88891 | 0.31896 |
| H | 3.70556 | 1.39672 | 1.02596 | H | 3.69302 | 1.42075 | 1.04634 |
| H | 3.40618 | 1.47008 | -0.71300 | H | 3.40359 | 1.48808 | -0.69497 |
| H | -1.24826 | 4.50501 | -0.43428 | H | -1.27346 | 4.49582 | -0.44384 |
| H | -0.09824 | 4.08402 | -1.73366 | H | -0.11746 | 4.08186 | -1.74032 |
| H | -1.58659 | 3.12414 | -1.51810 | H | -1.59915 | 3.11064 | -1.52585 |

| **4b2** | | | |
| --- | --- | --- | --- |
| C | 0.66764 | 0.26862 | 0.78596 |
| C | 2.08066 | 0.84492 | 1.03077 |
| C | 2.82698 | 1.06744 | -0.29038 |
| C | 2.82875 | -0.17835 | -1.1851 |
| C | 1.40834 | -0.72742 | -1.43383 |
| C | 0.67321 | -1.02849 | -0.08208 |
| C | -0.84035 | -1.36789 | -0.26806 |
| C | -1.49673 | -1.66033 | 1.08938 |
| C | -1.07099 | -0.81429 | 2.23038 |
| C | -0.06552 | 0.06397 | 2.10039 |
| O | 2.0019 | 2.04301 | 1.7938 |
| C | 1.44911 | -1.91661 | -2.40487 |
| C | 1.36352 | -2.21394 | 0.63545 |
| C | -1.60553 | -0.22294 | -0.99779 |
| O | -0.00305 | 1.33338 | 0.05486 |
| O | -2.35517 | -2.52129 | 1.21805 |
| C | -1.37885 | 1.12536 | -0.30272 |
| C | -3.10217 | -0.50505 | -1.18205 |
| H | -0.96328 | -2.2825 | -0.85648 |
| O | -1.7336 | 2.13346 | -1.196 |
| C | -1.83434 | 3.42913 | -0.61965 |
| H | 2.63252 | 0.13549 | 1.65818 |
| H | 3.84799 | 1.39164 | -0.0633 |
| H | 2.33708 | 1.89379 | -0.81846 |
| H | 3.28385 | 0.06987 | -2.1514 |
| H | 3.46785 | -0.9563 | -0.74787 |
| H | 0.86064 | 0.08096 | -1.92722 |
| H | -1.60259 | -0.96067 | 3.1669 |
| H | 0.24427 | 0.70247 | 2.92322 |
| H | 1.37946 | 2.61065 | 1.31443 |
| H | 0.45232 | -2.31075 | -2.62584 |
| H | 1.89181 | -1.60419 | -3.35645 |
| H | 2.05665 | -2.74199 | -2.01983 |
| H | 2.44287 | -2.07418 | 0.72611 |
| H | 0.97322 | -2.37645 | 1.6409 |
| H | 1.2 | -3.13642 | 0.07129 |
| H | -1.16246 | -0.12231 | -1.99398 |
| H | -1.99155 | 1.19985 | 0.61137 |
| H | -3.56196 | 0.29521 | -1.76685 |
| H | -3.25373 | -1.45168 | -1.70893 |
| H | -3.62787 | -0.57955 | -0.22547 |
| H | -2.23161 | 4.08692 | -1.39505 |
| H | -2.52072 | 3.43185 | 0.23926 |
| H | -0.85769 | 3.8051 | -0.29497 |

**Table S6b**.Optimized Z-Matrixes of paralemnanoid D (**4**) with simplified structures in the Gas Phase (Å) at B3LYP/6-31+G(d,p) level.

**Figure S14a**. Structures of isomers **6a**, **6b**, **6c** and **6d** for paralemnanoid F (**6**)

**Figure S14b**. DP4+ results obtained using experimental data of paralemnanoid F (**6**) *versus* isomers **6a**, **6b**, **6c** and **6d**

|  |  |  |
| --- | --- | --- |
| **6a1** (99.7%) | **6b1** (100.0%) | **6c1** (100.0%) |
|  |  |  |
| **6d1** (61.5%) | **6d2** (38.5%) |  |

**Table S7a**. Stable conformers of paralemnanoid F (**6**).

| **6a1** | | | | **6b1** | | | |
| --- | --- | --- | --- | --- | --- | --- | --- |
| C | -2.13035 | -1.08306 | -0.24946 | C | -2.03885 | -1.15414 | 0.63227 |
| H | -2.36513 | -1.09439 | -1.32018 | H | -2.08495 | -1.86045 | 1.47602 |
| C | -2.9642 | -0.0072 | 0.45671 | C | -2.84741 | 0.09843 | 0.98135 |
| H | -4.02263 | -0.19006 | 0.24366 | H | -3.91424 | -0.16245 | 1.01429 |
| H | -2.8284 | -0.13044 | 1.53747 | H | -2.56748 | 0.4063 | 1.99433 |
| C | -2.55707 | 1.41488 | 0.0508 | C | -2.5993 | 1.25134 | 0.00173 |
| H | -3.11422 | 2.13968 | 0.65657 | H | -3.13526 | 2.14342 | 0.34807 |
| H | -2.8512 | 1.61003 | -0.98855 | H | -3.01962 | 1.00577 | -0.98033 |
| C | -1.04679 | 1.67042 | 0.23842 | C | -1.09931 | 1.59189 | -0.1262 |
| H | -0.85113 | 1.51386 | 1.30351 | H | -0.76823 | 1.85105 | 0.88413 |
| C | -0.19083 | 0.61801 | -0.54768 | C | -0.2718 | 0.3404 | -0.58253 |
| C | -0.61683 | -0.81438 | -0.09174 | C | -0.54495 | -0.825 | 0.43018 |
| C | 1.33591 | 0.68954 | -0.21554 | C | 1.276476 | 0.56067 | -0.50492 |
| C | 2.11204 | -0.36783 | -1.01358 | C | 2.022022 | -0.70821 | -0.94393 |
| C | 1.4577 | -1.68621 | -1.19044 | C | 1.434962 | -2.00763 | -0.53777 |
| H | 2.04778 | -2.45784 | -1.67787 | H | 2.015008 | -2.8947 | -0.77759 |
| C | 0.19501 | -1.89041 | -0.78596 | C | 0.23868 | -2.06953 | 0.0657 |
| H | -0.28875 | -2.85738 | -0.89353 | H | -0.18651 | -3.02586 | 0.36018 |
| O | -2.45191 | -2.38507 | 0.22448 | O | -2.49728 | -1.79139 | -0.5613 |
| H | -2.07964 | -2.4296 | 1.11889 | H | -3.41105 | -2.06638 | -0.41211 |
| C | -0.697 | 3.13098 | -0.08292 | C | -0.89938 | 2.83449 | -1.00638 |
| H | -0.94887 | 3.39818 | -1.11421 | H | 0.155582 | 3.10732 | -1.10944 |
| H | 0.36501 | 3.34867 | 0.06748 | H | -1.41537 | 3.69252 | -0.56222 |
| H | -1.26221 | 3.80184 | 0.57258 | H | -1.30722 | 2.69532 | -2.0125 |
| O | -0.41017 | -0.94988 | 1.34456 | O | -0.14737 | -0.40914 | 1.76539 |
| C | 1.59774 | 0.49588 | 1.30936 | C | 1.730696 | 0.96744 | 0.92713 |
| C | 0.93425 | -0.79743 | 1.80513 | C | 1.225341 | -0.06195 | 1.94626 |
| H | 0.86432 | -0.80077 | 2.89723 | H | 1.284289 | 0.33801 | 2.96332 |
| H | 1.53856 | -1.66647 | 1.50933 | H | 1.854683 | -0.96293 | 1.90982 |
| C | 3.08271 | 0.52948 | 1.69099 | C | 3.243439 | 1.17971 | 1.06231 |
| H | 3.64407 | -0.29292 | 1.2386 | H | 3.806979 | 0.26257 | 0.86928 |
| H | 3.19968 | 0.46251 | 2.77828 | H | 3.492551 | 1.52618 | 2.07154 |
| H | 3.54872 | 1.46193 | 1.3593 | H | 3.596363 | 1.93307 | 0.35177 |
| C | -0.37685 | 0.82831 | -2.07018 | C | -0.62861 | -0.0174 | -2.04512 |
| H | 0.07875 | 1.77509 | -2.37364 | H | -0.27797 | 0.77676 | -2.71101 |
| H | -1.42918 | 0.86388 | -2.36035 | H | -1.70019 | -0.14783 | -2.18874 |
| H | 0.09319 | 0.03984 | -2.65938 | H | -0.15515 | -0.94527 | -2.36757 |
| H | 1.75401 | 1.65501 | -0.51825 | H | 1.587793 | 1.34337 | -1.20437 |
| O | 3.23497 | -0.1492 | -1.44618 | O | 3.076137 | -0.65247 | -1.56217 |
| H | 1.11151 | 1.3367 | 1.81678 | H | 1.246026 | 1.92521 | 1.1474 |
| **6c1** | | | | **6d1** | | | |
| C | -2.07785 | -1.09473 | -0.18221 | C | -1.97797 | -1.09837 | 0.73204 |
| C | -2.90025 | 0.133 | 0.22564 | C | -2.79316 | 0.19174 | 0.84915 |
| C | -2.39331 | 1.42301 | -0.4302 | C | -2.50385 | 1.17021 | -0.29459 |
| C | -0.88764 | 1.65493 | -0.18178 | C | -0.99826 | 1.48919 | -0.41763 |
| C | -0.03249 | 0.43582 | -0.67018 | C | -0.15334 | 0.18643 | -0.63075 |
| C | -0.56521 | -0.85528 | 0.02617 | C | -0.47579 | -0.80364 | 0.53914 |
| C | 1.47485 | 0.49563 | -0.25085 | C | 1.39585 | 0.39611 | -0.52573 |
| C | 2.2223 | -0.71916 | -0.82685 | C | 2.11608 | -0.93695 | -0.79139 |
| C | 1.50931 | -2.0188 | -0.76872 | C | 1.50594 | -2.14971 | -0.19253 |
| C | 0.2255 | -2.08354 | -0.38557 | C | 0.30575 | -2.09644 | 0.40323 |
| O | -2.49236 | -2.25688 | 0.52576 | O | -2.39131 | -1.911 | -0.3676 |
| C | -0.44814 | 3.00685 | -0.76321 | C | -0.76626 | 2.57201 | -1.48166 |
| O | -0.45665 | -0.68567 | 1.46929 | O | -0.12855 | -0.16227 | 1.79649 |
| C | 1.74219 | 0.48526 | 1.29315 | C | 1.92135 | 0.90656 | 0.85692 |
| C | 0.87074 | -0.58207 | 1.99198 | C | 1.25518 | 0.12 | 2.00575 |
| C | -0.10175 | 0.35104 | -2.2156 | C | -0.45092 | -0.3907 | -2.03739 |
| O | 3.36461 | -0.63144 | -1.25109 | O | 3.17149 | -0.98873 | -1.40498 |
| C | 1.68256 | 1.86606 | 1.96926 | C | 1.87405 | 2.43247 | 1.04714 |
| H | -2.24436 | -1.32006 | -1.24199 | H | -2.06168 | -1.66598 | 1.67217 |
| H | -3.9505 | -0.05117 | -0.02372 | H | -2.54848 | 0.65889 | 1.80888 |
| H | -2.84178 | 0.23092 | 1.31594 | H | -3.86172 | -0.06201 | 0.88515 |
| H | -2.60742 | 1.41017 | -1.50651 | H | -2.88811 | 0.76638 | -1.23785 |
| H | -2.94993 | 2.27845 | -0.02869 | H | -3.04837 | 2.10606 | -0.11936 |
| H | -0.77609 | 1.70386 | 0.9033 | H | -0.70634 | 1.90141 | 0.55033 |
| H | 1.96242 | 1.37604 | -0.68057 | H | 1.74465 | 1.08164 | -1.30408 |
| H | 2.07546 | -2.89737 | -1.06675 | H | 2.0738 | -3.07196 | -0.2811 |
| H | -0.30112 | -3.03173 | -0.31971 | H | -0.13431 | -2.98741 | 0.84465 |
| H | -2.17791 | -2.12555 | 1.43385 | H | -3.31561 | -2.15044 | -0.22185 |
| H | 0.6161 | 3.2048 | -0.6068 | H | 0.2929 | 2.81293 | -1.6111 |
| H | -1.00429 | 3.81815 | -0.28166 | H | -1.27709 | 3.49645 | -1.19139 |
| H | -0.64518 | 3.07396 | -1.83796 | H | -1.16251 | 2.27518 | -2.45789 |
| H | 2.78242 | 0.15357 | 1.41116 | H | 2.98809 | 0.64681 | 0.88339 |
| H | 0.73788 | -0.33907 | 3.05058 | H | 1.28494 | 0.69444 | 2.93682 |
| H | 1.3652 | -1.55946 | 1.93512 | H | 1.80117 | -0.81614 | 2.17915 |
| H | -1.12851 | 0.3839 | -2.58572 | H | -1.51912 | -0.50312 | -2.21797 |
| H | 0.43213 | 1.19703 | -2.65775 | H | -0.0359 | 0.27567 | -2.79981 |
| H | 0.35325 | -0.55891 | -2.60775 | H | -0.00701 | -1.37503 | -2.18759 |
| H | 2.30054 | 2.58991 | 1.42829 | H | 2.32653 | 2.94524 | 0.19212 |
| H | 0.67288 | 2.27373 | 2.04167 | H | 0.86391 | 2.82453 | 1.17824 |
| H | 2.0792 | 1.79277 | 2.98796 | H | 2.44937 | 2.70894 | 1.93777 |
| **6d2** | | | |  |  |  |  |
| C | -1.98084 | -1.11156 | 0.72967 |  |  |  |  |
| C | -2.8019 | 0.16917 | 0.84825 |  |  |  |  |
| C | -2.51161 | 1.15625 | -0.289 |  |  |  |  |
| C | -1.00793 | 1.48268 | -0.41502 |  |  |  |  |
| C | -0.15679 | 0.18362 | -0.62656 |  |  |  |  |
| C | -0.47354 | -0.79954 | 0.54915 |  |  |  |  |
| C | 1.39147 | 0.40166 | -0.53021 |  |  |  |  |
| C | 2.11354 | -0.92758 | -0.79939 |  |  |  |  |
| C | 1.52141 | -2.13973 | -0.17312 |  |  |  |  |
| C | 0.32731 | -2.08454 | 0.43438 |  |  |  |  |
| O | -2.51023 | -1.86076 | -0.36283 |  |  |  |  |
| C | -0.78211 | 2.56462 | -1.48139 |  |  |  |  |
| O | -0.12994 | -0.15394 | 1.80312 |  |  |  |  |
| C | 1.91825 | 0.91724 | 0.85106 |  |  |  |  |
| C | 1.25207 | 0.14192 | 2.00884 |  |  |  |  |
| C | -0.45873 | -0.40087 | -2.0297 |  |  |  |  |
| O | 3.15721 | -0.98674 | -1.43068 |  |  |  |  |
| C | 1.87355 | 2.44489 | 1.02802 |  |  |  |  |
| H | -2.07003 | -1.68678 | 1.66506 |  |  |  |  |
| H | -2.56427 | 0.63221 | 1.81098 |  |  |  |  |
| H | -3.86237 | -0.10411 | 0.8655 |  |  |  |  |
| H | -2.89968 | 0.76081 | -1.2338 |  |  |  |  |
| H | -3.05652 | 2.09006 | -0.1038 |  |  |  |  |
| H | -0.71504 | 1.89755 | 0.55161 |  |  |  |  |
| H | 1.73357 | 1.08865 | -1.31015 |  |  |  |  |
| H | 2.10614 | -3.05361 | -0.23759 |  |  |  |  |
| H | -0.0732 | -2.96697 | 0.93475 |  |  |  |  |
| H | -2.00164 | -2.67752 | -0.45357 |  |  |  |  |
| H | 0.27543 | 2.81163 | -1.61387 |  |  |  |  |
| H | -1.29732 | 3.48652 | -1.19123 |  |  |  |  |
| H | -1.17983 | 2.26509 | -2.4562 |  |  |  |  |
| H | 2.98464 | 0.656 | 0.88053 |  |  |  |  |
| H | 1.27711 | 0.72953 | 2.93164 |  |  |  |  |
| H | 1.80471 | -0.78772 | 2.19736 |  |  |  |  |
| H | -1.52669 | -0.53234 | -2.19802 |  |  |  |  |
| H | -0.06418 | 0.27161 | -2.7974 |  |  |  |  |
| H | 0.00821 | -1.37444 | -2.18857 |  |  |  |  |
| H | 2.33475 | 2.94891 | 0.17251 |  |  |  |  |
| H | 0.86312 | 2.83984 | 1.1464 |  |  |  |  |
| H | 2.44192 | 2.72723 | 1.92115 |  |  |  |  |

**Table S7b**.Optimized Z-Matrixes of paralemnanoid F (**6**) with simplified structures in the Gas Phase (Å) at B3LYP/6-31+G(d,p) level.

 **Figure S14c**. Experimental ECD spectra of paralemnanoid F (**6**)

 **Figure S15**. Experimental ECD spectra of paralemnanoid G (**7**).

**Figure S16a**. Structures of isomers **8a**, **8b, 8c** and **8d** for paralemnanoid H (**8**)

**Figure S16b**. DP4+ results obtained using experimental data of paralemnanoid H (**8**) *versus* isomers **8a**, **8b**, **8c** and **8d**.

|  |  |  |
| --- | --- | --- |
| **8a1** (18.0%) | **8a2** (19.2%) | **8a3** (46.3%) |
|  |  |  |
| **8a4** (16.5%) | **8b1** (99.1%) | **8c1** (100%) |
|  |  |  |
| **8d1** (99.9%) |  |  |

**Table S8a**. Stable conformers of paralemnanoid H (**8**)

| **8a1** | | | | **8a2** | | | |
| --- | --- | --- | --- | --- | --- | --- | --- |
| C | 2.21717 | -1.18988 | -1.02916 | C | 2.22409 | -1.17493 | -1.03551 |
| C | 3.25129 | -0.14841 | -0.90766 | C | 3.25897 | -0.13714 | -0.89461 |
| C | 3.03465 | 0.84835 | 0.21352 | C | 3.03426 | 0.85047 | 0.23365 |
| C | 1.54741 | 1.17995 | 0.42559 | C | 1.54523 | 1.17989 | 0.43484 |
| C | 0.67686 | -0.09839 | 0.67164 | C | 0.67459 | -0.101 | 0.66607 |
| C | 1.08231 | -1.21439 | -0.30172 | C | 1.08391 | -1.20837 | -0.31646 |
| C | -0.85920 | 0.24092 | 0.51682 | C | -0.86005 | 0.23733 | 0.50521 |
| C | -1.77879 | -0.99687 | 0.35617 | C | -1.77885 | -1.01031 | 0.35325 |
| C | -1.27287 | -2.02207 | -0.66103 | C | -1.27643 | -2.02616 | -0.668 |
| C | 0.17549 | -2.42137 | -0.37265 | C | 0.17922 | -2.41543 | -0.40257 |
| C | -1.35450 | 1.14373 | -0.64469 | C | -1.35683 | 1.1316 | -0.66139 |
| C | -2.81813 | 0.70965 | -0.80403 | C | -2.82562 | 0.7041 | -0.79356 |
| O | -3.02926 | -0.47152 | -0.16358 | O | -3.03709 | -0.46859 | -0.13886 |
| O | 4.24140 | -0.13218 | -1.62521 | O | 4.25588 | -0.11535 | -1.60239 |
| O | -3.69572 | 1.29328 | -1.38494 | O | -3.70866 | 1.29455 | -1.35982 |
| C | 1.41168 | 2.25784 | 1.51283 | C | 1.39819 | 2.254 | 1.52411 |
| C | 0.90716 | -0.63675 | 2.11323 | C | 0.89733 | -0.65319 | 2.10354 |
| H | -1.18397 | 0.71251 | 1.45177 | H | -1.177 | 0.73375 | 1.43424 |
| C | -1.31520 | 2.66860 | -0.48110 | C | -1.30484 | 2.65852 | -0.52213 |
| O | -2.02218 | -1.55619 | 1.61064 | O | -2.03482 | -1.697 | 1.53768 |
| H | 2.45146 | -2.00074 | -1.71517 | H | 2.46232 | -1.97808 | -1.72906 |
| H | 3.60874 | 1.75270 | -0.00916 | H | 3.6099 | 1.75653 | 0.02234 |
| H | 3.46827 | 0.41898 | 1.12812 | H | 3.46091 | 0.41421 | 1.14824 |
| H | 1.20223 | 1.60821 | -0.52231 | H | 1.20734 | 1.6108 | -0.51426 |
| H | -1.93983 | -2.89289 | -0.65237 | H | -1.93823 | -2.89588 | -0.63331 |
| H | -1.34157 | -1.58934 | -1.66472 | H | -1.36473 | -1.5874 | -1.66695 |
| H | 0.53809 | -3.11681 | -1.13475 | H | 0.53776 | -3.09339 | -1.18228 |
| H | 0.21368 | -2.95531 | 0.58571 | H | 0.23022 | -2.97032 | 0.54301 |
| H | -0.84237 | 0.87055 | -1.57917 | H | -0.85506 | 0.83889 | -1.59521 |
| H | 0.37150 | 2.48545 | 1.75796 | H | 0.35552 | 2.48707 | 1.75406 |
| H | 1.91505 | 1.95786 | 2.43691 | H | 1.88694 | 1.94953 | 2.45464 |
| H | 1.88077 | 3.18781 | 1.17576 | H | 1.876 | 3.18299 | 1.19704 |
| H | 0.30930 | -1.52653 | 2.30966 | H | 0.30268 | -1.54895 | 2.28432 |
| H | 1.96031 | -0.88549 | 2.27214 | H | 1.9495 | -0.90183 | 2.26734 |
| H | 0.61614 | 0.11129 | 2.85496 | H | 0.60499 | 0.0887 | 2.85225 |
| H | -1.92943 | 3.11960 | -1.26397 | H | -1.92339 | 3.10164 | -1.30598 |
| H | -0.30620 | 3.07449 | -0.55502 | H | -0.29382 | 3.05624 | -0.61155 |
| H | -1.73734 | 2.96805 | 0.48374 | H | -1.71622 | 2.9776 | 0.44129 |
| H | -2.64067 | -2.29284 | 1.49811 | H | -2.37719 | -1.06721 | 2.18839 |
| **8a3** | | | | **8a4** | | | |
| C | 2.15252 | -1.22256 | -1.07698 | C | 2.16196 | -1.19675 | -1.09583 |
| C | 3.29052 | -0.32519 | -0.82415 | C | 3.30186 | -0.31168 | -0.81094 |
| C | 3.07328 | 0.76782 | 0.20840 | C | 3.07528 | 0.77028 | 0.23204 |
| C | 1.60014 | 1.18014 | 0.38091 | C | 1.60061 | 1.18151 | 0.39368 |
| C | 0.67361 | -0.06269 | 0.61693 | C | 0.67352 | -0.06494 | 0.60661 |
| C | 0.95675 | -1.11717 | -0.46352 | C | 0.9593 | -1.09678 | -0.49506 |
| C | -0.83572 | 0.34409 | 0.55704 | C | -0.8366 | 0.33722 | 0.54605 |
| C | -1.81562 | -0.87029 | 0.64400 | C | -1.80902 | -0.89244 | 0.64521 |
| C | -1.22748 | -2.22848 | 0.23798 | C | -1.22863 | -2.22577 | 0.16625 |
| C | -0.16924 | -2.05889 | -0.85334 | C | -0.1711 | -2.01771 | -0.91719 |
| C | -1.29860 | 1.12053 | -0.69909 | C | -1.30694 | 1.10443 | -0.71127 |
| C | -2.67121 | 0.53819 | -1.02350 | C | -2.69914 | 0.54204 | -0.98807 |
| O | -2.87964 | -0.59817 | -0.30724 | O | -2.92637 | -0.56077 | -0.22619 |
| O | 4.35859 | -0.46200 | -1.40516 | O | 4.37827 | -0.44635 | -1.37674 |
| O | -3.48201 | 0.96417 | -1.80435 | O | -3.52124 | 0.97285 | -1.75489 |
| C | 1.49777 | 2.27613 | 1.45351 | C | 1.48634 | 2.2679 | 1.47438 |
| C | 0.97464 | -0.67091 | 2.01695 | C | 0.96913 | -0.70345 | 1.99414 |
| H | -1.06168 | 0.94805 | 1.44041 | H | -1.05195 | 0.95549 | 1.42561 |
| C | -1.39680 | 2.64792 | -0.59635 | C | -1.38001 | 2.63527 | -0.63195 |
| O | -2.36478 | -0.88431 | 1.92967 | O | -2.31232 | -1.10825 | 1.92924 |
| H | 2.33014 | -1.99770 | -1.81952 | H | 2.34354 | -1.95694 | -1.8526 |
| H | 3.68629 | 1.62788 | -0.07889 | H | 3.69127 | 1.63287 | -0.04104 |
| H | 3.48678 | 0.40812 | 1.16072 | H | 3.47978 | 0.40115 | 1.18447 |
| H | 1.28962 | 1.61749 | -0.57623 | H | 1.29999 | 1.62707 | -0.56285 |
| H | -0.79983 | -2.71921 | 1.11503 | H | -0.81036 | -2.75918 | 1.02204 |
| H | -2.04646 | -2.85947 | -0.12313 | H | -2.05858 | -2.82879 | -0.21109 |
| H | -0.66146 | -1.66116 | -1.75259 | H | -0.65704 | -1.58798 | -1.8055 |
| H | 0.24285 | -3.02875 | -1.14762 | H | 0.2365 | -2.97882 | -1.24447 |
| H | -0.65928 | 0.86841 | -1.55718 | H | -0.68884 | 0.82969 | -1.57717 |
| H | 0.47008 | 2.59899 | 1.63727 | H | 0.45835 | 2.60178 | 1.63732 |
| H | 1.92224 | 1.94947 | 2.40771 | H | 1.88559 | 1.92818 | 2.43489 |
| H | 2.06486 | 3.15669 | 1.13425 | H | 2.06968 | 3.14526 | 1.17638 |
| H | 0.37655 | -1.55593 | 2.22876 | H | 0.36105 | -1.5857 | 2.18746 |
| H | 2.02516 | -0.95899 | 2.10052 | H | 2.01694 | -1.00233 | 2.07107 |
| H | 0.75089 | 0.05534 | 2.80295 | H | 0.75486 | 0.00944 | 2.79553 |
| H | -1.92220 | 3.03287 | -1.47369 | H | -1.91318 | 3.0149 | -1.50674 |
| H | -0.41380 | 3.11942 | -0.54503 | H | -0.38956 | 3.09256 | -0.60219 |
| H | -1.96535 | 2.94312 | 0.29169 | H | -1.93036 | 2.95536 | 0.25945 |
| H | -2.99996 | -1.61400 | 1.97846 | H | -2.82385 | -0.32857 | 2.1898 |
| **8b1** | | | | **8c1** | | | |
| C | -2.53282 | -1.32562 | -0.33671 | C | 2.58355 | -1.16357 | 0.20489 |
| C | -3.53662 | -0.26986 | -0.13571 | C | 3.37578 | -0.11923 | -0.45681 |
| C | -2.98681 | 1.13052 | 0.05184 | C | 2.73961 | 1.25482 | -0.5302 |
| C | -1.58612 | 1.13459 | 0.68453 | C | 1.19572 | 1.26643 | -0.54113 |
| C | -0.56270 | 0.26823 | -0.13926 | C | 0.60307 | 0.38943 | 0.6174 |
| C | -1.19935 | -1.12101 | -0.35224 | C | 1.33655 | -0.96955 | 0.67251 |
| C | 0.78729 | 0.17992 | 0.70413 | C | -0.92568 | 0.03498 | 0.47651 |
| C | 1.53449 | -1.18813 | 0.67422 | C | -1.16626 | -1.25286 | -0.33091 |
| C | 0.71220 | -2.44805 | 0.48430 | C | -0.66842 | -2.49651 | 0.4072 |
| C | -0.28072 | -2.27217 | -0.66157 | C | 0.56454 | -2.12306 | 1.27585 |
| C | 2.02380 | 1.07932 | 0.34216 | C | -2.00843 | 0.96527 | -0.10543 |
| C | 2.77153 | 0.21903 | -0.66786 | C | -3.11018 | -0.03991 | -0.4722 |
| O | 2.43300 | -1.09432 | -0.46153 | O | -2.59558 | -1.31158 | -0.50593 |
| O | -4.73663 | -0.50761 | -0.14982 | O | 4.49333 | -0.342 | -0.9035 |
| C | -1.16101 | 2.57495 | 0.99090 | C | 0.75326 | 2.7406 | -0.55949 |
| C | -0.35029 | 0.82737 | -1.57613 | C | 0.80738 | 1.08086 | 1.99285 |
| C | 2.01588 | 2.56112 | -0.03510 | C | -2.5692 | 2.06451 | 0.79833 |
| O | 3.55457 | 0.55391 | -1.51684 | O | -4.27076 | 0.18526 | -0.68707 |
| O | 2.29241 | -1.25220 | 1.86180 | O | -0.56343 | -1.09446 | -1.59193 |
| H | 0.51469 | 0.34483 | 1.74964 | H | -1.27112 | -0.2073 | 1.49073 |
| H | -2.93722 | -2.31695 | -0.52765 | H | 3.05947 | -2.13872 | 0.27808 |
| H | -3.69569 | 1.69486 | 0.66556 | H | 3.13236 | 1.75895 | -1.41852 |
| H | -2.96972 | 1.62442 | -0.92999 | H | 3.11402 | 1.82787 | 0.33076 |
| H | -1.67992 | 0.61707 | 1.65060 | H | 0.87029 | 0.79497 | -1.47408 |
| H | 1.39519 | -3.28439 | 0.29311 | H | -1.47123 | -2.89641 | 1.03231 |
| H | 0.18252 | -2.65265 | 1.42034 | H | -0.40152 | -3.2696 | -0.3213 |
| H | 0.27838 | -2.07567 | -1.58539 | H | 1.21762 | -2.98978 | 1.40232 |
| H | -0.85980 | -3.18554 | -0.82081 | H | 0.21601 | -1.84445 | 2.27839 |
| H | 2.65044 | 1.00096 | 1.24100 | H | -1.67386 | 1.38951 | -1.05878 |
| H | -1.94958 | 3.06923 | 1.56746 | H | -0.3244 | 2.87708 | -0.64422 |
| H | -1.00519 | 3.16652 | 0.08331 | H | 1.20834 | 3.24028 | -1.42113 |
| H | -0.24897 | 2.62078 | 1.58882 | H | 1.09082 | 3.27548 | 0.33453 |
| H | -0.14369 | 1.89679 | -1.57511 | H | 1.84957 | 1.37376 | 2.14261 |
| H | 0.47732 | 0.32823 | -2.08646 | H | 0.18508 | 1.97531 | 2.08152 |
| H | -1.24442 | 0.66572 | -2.18251 | H | 0.53539 | 0.41055 | 2.81413 |
| H | 1.66667 | 3.18395 | 0.78975 | H | -3.45784 | 2.50292 | 0.33752 |
| H | 3.04717 | 2.84637 | -0.26084 | H | -1.83998 | 2.85893 | 0.97272 |
| H | 1.42331 | 2.79103 | -0.91927 | H | -2.86566 | 1.65588 | 1.77012 |
| H | 2.85922 | -2.03509 | 1.80468 | H | -0.74956 | -1.88627 | -2.1164 |
| **8d4** | | | |  |  |  |  |
| C | 2.63898 | -1.27502 | -0.27403 |  |  |  |  |
| C | 3.56845 | -0.14841 | -0.44638 |  |  |  |  |
| C | 3.01217 | 1.21702 | -0.10201 |  |  |  |  |
| C | 1.50253 | 1.33993 | -0.37314 |  |  |  |  |
| C | 0.67374 | 0.22173 | 0.35930 |  |  |  |  |
| C | 1.35375 | -1.15071 | 0.11420 |  |  |  |  |
| C | -0.73677 | 0.10239 | -0.31797 |  |  |  |  |
| C | -1.54434 | -1.16006 | 0.03643 |  |  |  |  |
| C | -0.81274 | -2.42884 | -0.35648 |  |  |  |  |
| C | 0.54800 | -2.41356 | 0.36357 |  |  |  |  |
| C | -1.85819 | 1.17752 | -0.34798 |  |  |  |  |
| C | -3.04393 | 0.33488 | -0.84430 |  |  |  |  |
| O | -2.76212 | -1.00713 | -0.71276 |  |  |  |  |
| O | 4.72460 | -0.31553 | -0.80969 |  |  |  |  |
| C | 1.07289 | 2.78793 | -0.09020 |  |  |  |  |
| C | 0.62947 | 0.46607 | 1.88881 |  |  |  |  |
| C | -2.27454 | 1.91808 | 0.93759 |  |  |  |  |
| O | -4.08847 | 0.72289 | -1.29170 |  |  |  |  |
| O | -1.87174 | -1.16090 | 1.41221 |  |  |  |  |
| H | 3.07046 | -2.25837 | -0.44650 |  |  |  |  |
| H | 3.22763 | 1.40705 | 0.95908 |  |  |  |  |
| H | 3.57239 | 1.96732 | -0.66802 |  |  |  |  |
| H | 1.36251 | 1.15965 | -1.44921 |  |  |  |  |
| H | -0.49835 | -0.05574 | -1.37909 |  |  |  |  |
| H | -1.39402 | -3.31410 | -0.07291 |  |  |  |  |
| H | -0.68255 | -2.45453 | -1.44290 |  |  |  |  |
| H | 1.14087 | -3.28507 | 0.07370 |  |  |  |  |
| H | 0.35837 | -2.50184 | 1.44087 |  |  |  |  |
| H | -1.66782 | 1.92020 | -1.12813 |  |  |  |  |
| H | 0.03122 | 2.98506 | -0.34455 |  |  |  |  |
| H | 1.68539 | 3.46928 | -0.68966 |  |  |  |  |
| H | 1.22524 | 3.05872 | 0.95947 |  |  |  |  |
| H | 0.13861 | 1.41009 | 2.12885 |  |  |  |  |
| H | 0.08249 | -0.32385 | 2.40240 |  |  |  |  |
| H | 1.64398 | 0.50117 | 2.29625 |  |  |  |  |
| H | -3.21000 | 2.45179 | 0.74538 |  |  |  |  |
| H | -1.52460 | 2.65412 | 1.23337 |  |  |  |  |
| H | -2.43202 | 1.22354 | 1.76364 |  |  |  |  |
| H | -2.52495 | -1.86293 | 1.54917 |  |  |  |  |

**Table S8b**.Optimized Z-Matrixes of paralemnanoid H (**8**) with simplified structures in the Gas Phase (Å) at B3LYP/6-31+G(d,p) level.

**Figure S17a**. Structures of isomers **9a**, **9b, 9c** and **9d** for paralemnanoid I (**9**)

**Figure S17b**. DP4+ results obtained using experimental data of paralemnanoid I (**9**) *versus* isomers **9a**, **9b**, **9c** and **9d**.

|  |  |  |
| --- | --- | --- |
| **9a1** (84.3%) | **9a2** (10.2%) | **9a3** (2.4%) |
|  |  |  |
| **9a4** (3.1%) | **9b1** (14.4%) | **9b2** (42.8%) |
|  |  |  |
| **9b3** (38.2%) | **9b4** (4.5%) | **9c1** (100%) |
|  |  |  |
| **9d1** (99.9%) |  |  |

**Table S9a**. Stable conformers of paralemnanoid I (**9**)

| **9a1** | | | | **9a2** | | | |
| --- | --- | --- | --- | --- | --- | --- | --- |
| C | -2.84818 | 1.28887 | 0.40913 | C | -2.94094 | -1.38776 | -0.5536 |
| H | -3.25537 | 2.24201 | 0.73988 | H | -3.26119 | -2.37763 | -0.87058 |
| C | -3.65252 | 0.56432 | -0.58984 | C | -4.0269 | -0.45853 | -0.21507 |
| C | -2.94846 | -0.60872 | -1.25036 | C | -3.59596 | 0.94106 | 0.17877 |
| H | -3.70049 | -1.25254 | -1.71515 | H | -3.64948 | 1.57798 | -0.71557 |
| H | -2.33786 | -0.19846 | -2.06597 | H | -4.3377 | 1.33834 | 0.87885 |
| C | -2.07897 | -1.39532 | -0.25092 | C | -2.18666 | 0.98023 | 0.79046 |
| C | -1.01365 | -0.47116 | 0.44684 | C | -1.10929 | 0.33092 | -0.15699 |
| C | -1.64158 | 0.86963 | 0.84062 | C | -1.62907 | -1.06939 | -0.54371 |
| C | 0.15741 | -0.12191 | -0.55389 | C | 0.26658 | 0.25651 | 0.64483 |
| C | 1.05826 | 1.06262 | -0.09134 | C | 1.12859 | -1.03034 | 0.44821 |
| C | 0.40522 | 2.21673 | 0.68149 | C | 0.40019 | -2.32539 | 0.12011 |
| H | 1.17959 | 2.75071 | 1.24091 | H | 1.13206 | -3.07921 | -0.18506 |
| H | -0.00101 | 2.90289 | -0.06824 | H | -0.09589 | -2.68435 | 1.02798 |
| C | -0.73983 | 1.78843 | 1.62113 | C | -0.62123 | -2.09154 | -0.99035 |
| H | -1.28702 | 2.67519 | 1.95481 | H | -1.12575 | -3.02294 | -1.26253 |
| H | -0.33363 | 1.30188 | 2.51226 | H | -0.08848 | -1.73648 | -1.88172 |
| C | 1.28258 | -1.16576 | -0.83247 | C | 1.42686 | 1.29745 | 0.42113 |
| C | 2.33543 | -0.86203 | 0.24792 | C | 2.28923 | 0.65436 | -0.67377 |
| O | 2.03664 | 0.44799 | 0.75085 | O | 1.97619 | -0.73862 | -0.66496 |
| C | -3.00423 | -2.1422 | 0.73178 | C | -1.88307 | 2.39596 | 1.29338 |
| H | -2.46103 | -2.84466 | 1.36798 | H | -2.70509 | 2.73793 | 1.9309 |
| H | -3.74444 | -2.71945 | 0.16877 | H | -1.78654 | 3.11667 | 0.47526 |
| H | -3.55308 | -1.45351 | 1.38222 | H | -0.97255 | 2.43549 | 1.89405 |
| C | -0.49061 | -1.15718 | 1.73925 | C | -0.99414 | 1.09165 | -1.50768 |
| H | -1.25960 | -1.15567 | 2.51608 | H | -0.82438 | 2.15829 | -1.36523 |
| H | 0.38893 | -0.6532 | 2.13735 | H | -1.91025 | 0.97722 | -2.09207 |
| H | -0.22059 | -2.19833 | 1.55074 | H | -0.17559 | 0.69604 | -2.11405 |
| O | -4.77309 | 0.93164 | -0.91511 | O | -5.2046 | -0.78775 | -0.27367 |
| C | 0.99142 | -2.657 | -1.00994 | C | 1.26596 | 2.80737 | 0.21984 |
| H | 1.93482 | -3.17671 | -1.20745 | H | 2.27076 | 3.23418 | 0.13544 |
| H | 0.33717 | -2.83545 | -1.86846 | H | 0.78166 | 3.2872 | 1.07151 |
| H | 0.53453 | -3.12427 | -0.13431 | H | 0.7189 | 3.08383 | -0.68312 |
| H | -0.30255 | 0.17243 | -1.50053 | H | -0.00139 | 0.27161 | 1.70454 |
| H | -1.53226 | -2.15683 | -0.81466 | H | -2.21292 | 0.3265 | 1.67449 |
| H | 1.74872 | -0.82229 | -1.76036 | H | 2.03425 | 1.18164 | 1.32356 |
| H | 2.29101 | -1.54172 | 1.11237 | H | 2.06676 | 1.02327 | -1.68779 |
| O | 3.60942 | -0.93324 | -0.32855 | O | 3.63865 | 0.8967 | -0.3851 |
| C | 4.67179 | -0.86456 | 0.61281 | C | 4.52767 | 0.51901 | -1.42749 |
| H | 4.59798 | -1.66912 | 1.3596 | H | 4.49362 | -0.56095 | -1.61458 |
| H | 4.68612 | 0.09954 | 1.13507 | H | 5.5325 | 0.79758 | -1.10363 |
| H | 5.60071 | -0.98519 | 0.05182 | H | 4.29464 | 1.04731 | -2.36394 |
| O | 1.64591 | 1.55059 | -1.29396 | O | 1.86746 | -1.15265 | 1.65921 |
| C | 2.75062 | 2.43395 | -1.15006 | C | 2.97798 | -2.0408 | 1.64352 |
| H | 3.54092 | 1.98721 | -0.53942 | H | 3.67544 | -1.79054 | 0.83853 |
| H | 2.45825 | 3.39915 | -0.71535 | H | 2.67203 | -3.09083 | 1.54556 |
| H | 3.13080 | 2.60849 | -2.15866 | H | 3.47809 | -1.91409 | 2.60591 |
| **9a3** | | | | **9a4** | | | |
| C | -2.79212 | 1.29879 | 0.06067 | C | -2.86306 | 0.94869 | 0.72289 |
| H | -3.13972 | 2.31842 | 0.21477 | H | -3.26074 | 1.79004 | 1.28601 |
| C | -3.74000 | 0.41969 | -0.63602 | C | -3.7387 | 0.42491 | -0.33441 |
| C | -3.32013 | -1.03271 | -0.76728 | C | -3.20276 | -0.75058 | -1.13432 |
| H | -3.73615 | -1.58391 | 0.08826 | H | -3.63903 | -1.66241 | -0.70142 |
| H | -3.79962 | -1.44678 | -1.65978 | H | -3.61156 | -0.67926 | -2.14733 |
| C | -1.79512 | -1.1975 | -0.83517 | C | -1.66741 | -0.85129 | -1.17184 |
| C | -1.05881 | -0.52482 | 0.37875 | C | -1.02341 | -0.7341 | 0.25711 |
| C | -1.59978 | 0.90496 | 0.55457 | C | -1.63621 | 0.46002 | 0.98998 |
| C | 0.48448 | -0.4675 | 0.02391 | C | 0.52905 | -0.51057 | 0.07144 |
| C | 1.18680 | 0.88189 | 0.3207 | C | 1.03623 | 0.95487 | 0.17356 |
| C | 0.66507 | 1.54856 | 1.58841 | C | 0.42621 | 1.80696 | 1.31695 |
| H | 0.81452 | 0.85727 | 2.42175 | H | 1.21144 | 2.05429 | 2.03624 |
| H | 1.24901 | 2.44255 | 1.82695 | H | 0.05348 | 2.7472 | 0.90329 |
| C | -0.81964 | 1.89006 | 1.41286 | C | -0.75158 | 1.10062 | 2.02147 |
| H | -0.92970 | 2.88222 | 0.96401 | H | -1.31223 | 1.82274 | 2.62229 |
| H | -1.29829 | 1.95372 | 2.39887 | H | -0.37064 | 0.33811 | 2.70967 |
| C | 1.54452 | -1.52356 | 0.48791 | C | 1.6346 | -1.32157 | 0.81481 |
| C | 2.82037 | -0.76296 | 0.09271 | C | 2.85599 | -0.52566 | 0.33089 |
| O | 2.57606 | 0.58451 | 0.48631 | O | 2.45196 | 0.83913 | 0.40583 |
| C | -1.43203 | -2.6699 | -1.07302 | C | -1.26223 | -2.11809 | -1.94184 |
| H | -1.93389 | -3.03043 | -1.97687 | H | -1.66235 | -2.07668 | -2.96014 |
| H | -1.75592 | -3.3127 | -0.2475 | H | -1.66514 | -3.0214 | -1.47135 |
| H | -0.35909 | -2.81764 | -1.21871 | H | -0.17813 | -2.23808 | -2.026 |
| C | -1.39343 | -1.26183 | 1.7023 | C | -1.34501 | -2.00339 | 1.09077 |
| H | -0.87143 | -0.8246 | 2.5565 | H | -0.7534 | -2.8619 | 0.76145 |
| H | -1.13065 | -2.32001 | 1.65586 | H | -2.40176 | -2.26622 | 0.99661 |
| H | -2.46471 | -1.19348 | 1.90917 | H | -1.15402 | -1.85066 | 2.15449 |
| O | -4.81992 | 0.82614 | -1.04618 | O | -4.84652 | 0.90015 | -0.54935 |
| C | 1.65575 | -1.94951 | 1.96168 | C | 1.66146 | -1.3972 | 2.34898 |
| H | 0.81761 | -2.56159 | 2.29118 | H | 2.54159 | -1.9677 | 2.66604 |
| H | 1.74395 | -1.09451 | 2.63756 | H | 0.78801 | -1.90042 | 2.76358 |
| H | 2.56193 | -2.55248 | 2.08717 | H | 1.7403 | -0.40648 | 2.80577 |
| H | 0.53023 | -0.52598 | -1.06621 | H | 0.73436 | -0.74782 | -0.9728 |
| H | -1.46275 | -0.6324 | -1.71654 | H | -1.29484 | 0.01821 | -1.72775 |
| H | 1.46876 | -2.42388 | -0.12723 | H | 1.68428 | -2.33565 | 0.40724 |
| H | 3.72058 | -1.07387 | 0.64298 | H | 3.7375 | -0.60943 | 0.98359 |
| O | 3.02575 | -0.9132 | -1.28687 | O | 3.18396 | -0.94255 | -0.96871 |
| C | 4.16772 | -0.22889 | -1.78009 | C | 4.2958 | -0.26146 | -1.53065 |
| H | 4.06577 | 0.85608 | -1.66966 | H | 5.19027 | -0.36731 | -0.89831 |
| H | 4.25226 | -0.47894 | -2.83938 | H | 4.08739 | 0.80504 | -1.66918 |
| H | 5.08275 | -0.55263 | -1.26121 | H | 4.49043 | -0.72154 | -2.50138 |
| O | 0.97730 | 1.69149 | -0.82467 | O | 0.78793 | 1.52744 | -1.10051 |
| C | 1.61865 | 2.96037 | -0.82517 | C | 1.36601 | 2.80097 | -1.3557 |
| H | 2.67966 | 2.8721 | -0.56743 | H | 0.89411 | 3.60463 | -0.77441 |
| H | 1.14153 | 3.67258 | -0.13788 | H | 1.20093 | 3.00171 | -2.41622 |
| H | 1.52592 | 3.34872 | -1.84165 | H | 2.44119 | 2.79893 | -1.14861 |
| **9b1** | | | | **9b2** | | | |
| C | 2.32260 | 0.56004 | 1.69615 | C | -2.26115 | -0.89948 | -1.55653 |
| H | 2.36752 | 0.90806 | 2.72575 | H | -2.27399 | -1.51572 | -2.45309 |
| C | 3.50076 | -0.18857 | 1.23296 | C | -3.53224 | -0.83162 | -0.82418 |
| C | 3.54203 | -0.47264 | -0.25586 | C | -3.54526 | 0.02469 | 0.43139 |
| H | 4.00281 | 0.39656 | -0.74679 | H | -3.95269 | 1.00692 | 0.15417 |
| H | 4.21272 | -1.31973 | -0.42935 | H | -4.26672 | -0.41299 | 1.12877 |
| C | 2.14652 | -0.74724 | -0.84194 | C | -2.1667 | 0.18358 | 1.09822 |
| H | 1.77451 | -1.63167 | -0.31277 | H | -1.87531 | -0.81566 | 1.44599 |
| C | 1.12947 | 0.40539 | -0.54163 | C | -1.07122 | 0.63021 | 0.06987 |
| C | 1.27348 | 0.87975 | 0.91075 | C | -1.13072 | -0.27439 | -1.16753 |
| C | -0.35191 | -0.07305 | -0.81399 | C | 0.36038 | 0.54073 | 0.69251 |
| C | -1.45542 | 0.84913 | -0.22864 | C | 1.50515 | 0.90079 | -0.30125 |
| C | -1.20386 | 1.22486 | 1.23802 | C | 1.17119 | 0.65119 | -1.78766 |
| H | -1.97143 | 1.92709 | 1.57735 | H | 0.7918 | 1.57114 | -2.24067 |
| H | -1.32477 | 0.32196 | 1.84272 | H | 2.09589 | 0.39106 | -2.3103 |
| C | 0.19931 | 1.80379 | 1.43619 | C | 0.15127 | -0.48028 | -1.9511 |
| H | 0.37997 | 2.01667 | 2.49404 | H | 0.61769 | -1.41192 | -1.60276 |
| H | 0.26796 | 2.7633 | 0.90659 | H | -0.08372 | -0.63433 | -3.00921 |
| C | -0.80964 | -1.47109 | -0.30512 | C | 0.764 | -0.83915 | 1.28372 |
| H | -0.36654 | -1.67746 | 0.67571 | H | 0.1532 | -1.63482 | 0.84465 |
| C | -2.33161 | -1.29649 | -0.07915 | C | 2.20776 | -1.07378 | 0.79792 |
| H | -2.94087 | -1.87912 | -0.78666 | H | 2.93057 | -1.1951 | 1.61916 |
| O | -2.65208 | 0.06288 | -0.31801 | O | 2.61439 | 0.06721 | 0.07365 |
| C | 2.27847 | -1.10259 | -2.33179 | C | -2.30026 | 1.09136 | 2.3317 |
| H | 2.82562 | -0.32867 | -2.8788 | H | -2.97703 | 0.62956 | 3.05846 |
| H | 1.31213 | -1.23757 | -2.82389 | H | -2.7246 | 2.06592 | 2.07082 |
| H | 2.83976 | -2.03618 | -2.44394 | H | -1.3473 | 1.26852 | 2.83685 |
| C | 1.42512 | 1.62743 | -1.45816 | C | -1.34466 | 2.0991 | -0.36505 |
| H | 1.30778 | 1.35695 | -2.51072 | H | -2.35869 | 2.20688 | -0.75778 |
| H | 2.44715 | 1.98805 | -1.30833 | H | -0.657 | 2.4421 | -1.13618 |
| H | 0.73575 | 2.44836 | -1.26504 | H | -1.22758 | 2.77636 | 0.48521 |
| O | 4.41029 | -0.50316 | 1.98901 | O | -4.53407 | -1.42079 | -1.21039 |
| C | -0.56694 | -2.67361 | -1.22774 | C | 0.67757 | -0.93759 | 2.8125 |
| H | 0.48789 | -2.93022 | -1.3296 | H | 1.03951 | -1.91315 | 3.15399 |
| H | -0.96623 | -2.48536 | -2.2311 | H | -0.34665 | -0.82211 | 3.17338 |
| H | -1.07877 | -3.55399 | -0.82471 | H | 1.29058 | -0.16488 | 3.29043 |
| H | -0.51261 | -0.04107 | -1.89781 | H | 0.43689 | 1.29516 | 1.4807 |
| O | -2.63390 | -1.70834 | 1.23301 | O | 2.22251 | -2.24331 | 0.00762 |
| C | -4.02593 | -1.71188 | 1.52181 | C | 3.52488 | -2.63755 | -0.40545 |
| H | -4.57373 | -2.37354 | 0.8343 | H | 3.99233 | -1.87902 | -1.04358 |
| H | -4.45171 | -0.70428 | 1.45448 | H | 3.41174 | -3.56679 | -0.96721 |
| H | -4.13761 | -2.08711 | 2.54096 | H | 4.17743 | -2.82011 | 0.46113 |
| O | -1.59067 | 1.96406 | -1.07432 | O | 1.85464 | 2.24217 | -0.04876 |
| C | -2.71943 | 2.80642 | -0.85721 | C | 2.9939 | 2.73695 | -0.745 |
| H | -2.72585 | 3.51698 | -1.68621 | H | 2.8319 | 2.78262 | -1.82994 |
| H | -2.64848 | 3.36885 | 0.08273 | H | 3.88056 | 2.12848 | -0.53945 |
| H | -3.65014 | 2.23166 | -0.86412 | H | 3.15798 | 3.75103 | -0.37498 |
| **9b3** | | | | **9b4** | | | |
| C | -2.34189 | -1.51789 | -0.87237 | C | 2.36601 | 1.20929 | -0.96176 |
| H | -2.41035 | -2.48319 | -1.36957 | H | 2.52944 | 2.16352 | -1.45864 |
| C | -3.55986 | -1.08145 | -0.17921 | C | 3.54429 | 0.6446 | -0.29488 |
| C | -3.49249 | 0.26595 | 0.52054 | C | 3.35311 | -0.70513 | 0.37635 |
| H | -3.92516 | 1.01172 | -0.1609 | H | 3.69719 | -1.47814 | -0.32498 |
| H | -4.16068 | 0.22759 | 1.38688 | H | 4.03137 | -0.75312 | 1.23438 |
| C | -2.07232 | 0.68436 | 0.94254 | C | 1.90237 | -0.96951 | 0.8129 |
| H | -1.74833 | -0.04116 | 1.69949 | H | 1.66949 | -0.20064 | 1.55875 |
| C | -1.05392 | 0.56795 | -0.24425 | C | 0.88852 | -0.7484 | -0.3609 |
| C | -1.19210 | -0.81241 | -0.89929 | C | 1.15102 | 0.6224 | -0.99442 |
| C | 0.41885 | 0.75416 | 0.25296 | C | -0.60742 | -0.93402 | 0.12069 |
| C | 1.48988 | 0.57035 | -0.8762 | C | -1.69143 | -0.22002 | -0.74667 |
| C | 1.05058 | -0.34855 | -2.02852 | C | -1.18924 | 0.44528 | -2.02816 |
| H | 0.64538 | 0.2657 | -2.83555 | H | -0.96216 | -0.32033 | -2.77498 |
| H | 1.95073 | -0.83076 | -2.41891 | H | -1.99069 | 1.06687 | -2.43795 |
| C | 0.03173 | -1.39683 | -1.57654 | C | 0.02396 | 1.32394 | -1.72683 |
| H | 0.52578 | -2.07168 | -0.86456 | H | -0.31125 | 2.15096 | -1.09122 |
| H | -0.27726 | -2.02016 | -2.42188 | H | 0.40905 | 1.77552 | -2.64707 |
| C | 0.88044 | -0.18708 | 1.39987 | C | -1.01272 | -0.5109 | 1.56489 |
| H | 0.24804 | -1.07992 | 1.4378 | H | -0.15749 | -0.37336 | 2.22547 |
| C | 2.28695 | -0.66104 | 0.97918 | C | -1.68863 | 0.84462 | 1.36935 |
| H | 3.06890 | -0.41278 | 1.71336 | H | -2.51545 | 1.03509 | 2.06985 |
| O | 2.63521 | 0.01298 | -0.21095 | O | -2.25067 | 0.82578 | 0.06628 |
| C | -2.13151 | 2.06729 | 1.61206 | C | 1.80955 | -2.3422 | 1.49594 |
| H | -2.58061 | 2.8162 | 0.95236 | H | 0.80034 | -2.57742 | 1.84324 |
| H | -1.14967 | 2.43814 | 1.9179 | H | 2.47008 | -2.36639 | 2.36907 |
| H | -2.75517 | 2.01554 | 2.5108 | H | 2.1296 | -3.1491 | 0.82877 |
| C | -1.37102 | 1.66293 | -1.30309 | C | 1.13138 | -1.81382 | -1.47444 |
| H | -2.40519 | 1.5854 | -1.64741 | H | 1.01402 | -2.82392 | -1.07121 |
| H | -0.72749 | 1.59683 | -2.17845 | H | 2.13307 | -1.72499 | -1.90123 |
| H | -1.22430 | 2.65937 | -0.87652 | H | 0.41877 | -1.71291 | -2.29456 |
| O | -4.58137 | -1.75703 | -0.17471 | O | 4.62857 | 1.21481 | -0.29943 |
| C | 0.91153 | 0.45483 | 2.79293 | C | -1.98154 | -1.52145 | 2.20074 |
| H | 1.54348 | 1.35087 | 2.79976 | H | -1.50368 | -2.49908 | 2.31817 |
| H | 1.31753 | -0.24615 | 3.52983 | H | -2.86673 | -1.65728 | 1.57401 |
| H | -0.08490 | 0.7472 | 3.13116 | H | -2.30177 | -1.18674 | 3.19311 |
| H | 0.51226 | 1.78766 | 0.59915 | H | -0.81566 | -2.00075 | 0.019 |
| O | 2.24980 | -2.06091 | 0.81314 | O | -0.7205 | 1.85731 | 1.50505 |
| C | 3.51856 | -2.63719 | 0.52619 | C | -1.26048 | 3.17221 | 1.49184 |
| H | 3.36816 | -3.71645 | 0.46098 | H | -0.42723 | 3.85959 | 1.64974 |
| H | 4.24084 | -2.42391 | 1.328 | H | -1.9948 | 3.30691 | 2.29983 |
| H | 3.92420 | -2.2657 | -0.42135 | H | -1.74605 | 3.40259 | 0.53591 |
| O | 1.88317 | 1.77258 | -1.49803 | O | -2.68242 | -1.20427 | -0.99884 |
| C | 2.64127 | 2.69547 | -0.72649 | C | -3.88521 | -0.75062 | -1.61111 |
| H | 3.58169 | 2.25478 | -0.38387 | H | -4.31384 | 0.09829 | -1.06856 |
| H | 2.08890 | 3.07277 | 0.14528 | H | -4.58059 | -1.59176 | -1.57264 |
| H | 2.85040 | 3.53595 | -1.39122 | H | -3.736 | -0.47008 | -2.66193 |
| **9c1** | | | | **9d1** | | | |
| C | 2.94987 | -1.15007 | 0.44222 | C | 2.89785 | -1.16745 | 0.31062 |
| H | 3.34038 | -2.10929 | 0.7757 | H | 3.32607 | -2.13585 | 0.55888 |
| C | 3.91318 | -0.30164 | -0.27031 | C | 3.62244 | -0.39406 | -0.70297 |
| C | 3.42834 | 1.09204 | -0.62662 | C | 3.05038 | 0.97026 | -1.03276 |
| H | 3.70606 | 1.76691 | 0.1956 | H | 3.59733 | 1.70326 | -0.42124 |
| H | 3.98542 | 1.43403 | -1.50449 | H | 3.30949 | 1.19797 | -2.0715 |
| C | 1.91551 | 1.13653 | -0.88345 | C | 1.52994 | 1.1239 | -0.81064 |
| H | 1.72994 | 0.45206 | -1.72327 | H | 1.02226 | 0.47074 | -1.52708 |
| C | 1.07794 | 0.55556 | 0.31292 | C | 1.09649 | 0.61614 | 0.60987 |
| C | 1.68770 | -0.78634 | 0.75162 | C | 1.75781 | -0.75055 | 0.89358 |
| C | -0.40125 | 0.31466 | -0.21297 | C | -0.45102 | 0.38153 | 0.80569 |
| C | -1.02557 | -1.0586 | 0.18015 | C | -0.9189 | -1.02824 | 0.39543 |
| C | -0.64091 | -1.48697 | 1.58723 | C | -0.32394 | -2.1215 | 1.29629 |
| H | -0.97780 | -0.71854 | 2.28679 | H | -1.02392 | -2.34351 | 2.10598 |
| H | -1.17349 | -2.4092 | 1.83502 | H | -0.18339 | -3.03918 | 0.71938 |
| C | 0.87897 | -1.69186 | 1.66738 | C | 1.03763 | -1.64959 | 1.87188 |
| H | 1.12825 | -2.72618 | 1.41788 | H | 1.66245 | -2.51009 | 2.1245 |
| H | 1.21672 | -1.53534 | 2.70057 | H | 0.85956 | -1.10583 | 2.80839 |
| C | -1.58803 | 1.31648 | -0.01629 | C | -1.55448 | 1.25938 | 0.17512 |
| H | -1.49348 | 2.14549 | -0.72565 | H | -1.36391 | 1.38725 | -0.89257 |
| C | -2.73569 | 0.39792 | -0.48247 | C | -2.79356 | 0.34008 | 0.2643 |
| H | -2.75550 | 0.31038 | -1.58491 | H | -3.46078 | 0.6053 | 1.10004 |
| O | -2.43210 | -0.89231 | 0.07168 | O | -2.32898 | -0.9863 | 0.54444 |
| C | 1.49649 | 2.54199 | -1.33629 | C | 1.17385 | 2.58319 | -1.14816 |
| H | 1.70444 | 3.29671 | -0.57086 | H | 0.10458 | 2.79027 | -1.13432 |
| H | 0.43478 | 2.60288 | -1.58953 | H | 1.53093 | 2.81281 | -2.15771 |
| H | 2.06125 | 2.82579 | -2.23032 | H | 1.66523 | 3.28845 | -0.46923 |
| C | 1.18185 | 1.48248 | 1.5523 | C | 1.57994 | 1.59365 | 1.71635 |
| H | 2.22034 | 1.53713 | 1.89012 | H | 1.04459 | 2.54535 | 1.66505 |
| H | 0.84968 | 2.49765 | 1.33123 | H | 1.40811 | 1.17499 | 2.71293 |
| H | 0.58845 | 1.11138 | 2.39032 | H | 2.64983 | 1.7998 | 1.6294 |
| O | 5.05181 | -0.67758 | -0.51889 | O | 4.64151 | -0.81566 | -1.23572 |
| C | -1.90357 | 1.91681 | 1.36333 | C | -1.82263 | 2.61317 | 0.83713 |
| H | -1.94857 | 1.16375 | 2.15335 | H | -2.70652 | 3.08324 | 0.39337 |
| H | -2.88611 | 2.39052 | 1.31838 | H | -0.98679 | 3.30642 | 0.7174 |
| H | -1.17868 | 2.67692 | 1.65155 | H | -2.00823 | 2.4959 | 1.91125 |
| H | -0.29132 | 0.25919 | -1.30127 | H | -0.62229 | 0.41926 | 1.89054 |
| O | -3.97272 | 0.82978 | -0.03296 | O | -3.50568 | 0.41535 | -0.93818 |
| C | -5.06633 | 0.05554 | -0.51248 | C | -4.77344 | -0.22725 | -0.89366 |
| H | -5.97592 | 0.55022 | -0.16697 | H | -5.41153 | 0.20236 | -0.1072 |
| H | -5.03314 | -0.96612 | -0.11988 | H | -4.67229 | -1.30424 | -0.71583 |
| H | -5.07389 | 0.01687 | -1.61176 | H | -5.24593 | -0.06307 | -1.8641 |
| O | -0.57544 | -2.12587 | -0.64552 | O | -0.54143 | -1.20111 | -0.96439 |
| C | -1.04677 | -2.1718 | -1.98149 | C | -1.19912 | -2.23714 | -1.68165 |
| H | -2.14127 | -2.18822 | -2.01889 | H | -2.265 | -2.02108 | -1.79871 |
| H | -0.65822 | -3.09887 | -2.40801 | H | -1.08485 | -3.21828 | -1.2016 |
| H | -0.68097 | -1.33466 | -2.5938 | H | -0.72373 | -2.27343 | -2.66387 |

**Table S****9b**.Optimized Z-Matrixes of paralemnanoid I (**9**) with simplified structures in the Gas Phase (Å) at B3LYP/6-31+G(d,p) level.

**Figure S18a**. Structures of isomers **10a**, **10b, 10c** and **10d** for paralemnanoid J (**10**).

**Figure S18b**. DP4+ results obtained using experimental data of paralemnanoid J (**10**) *versus* isomers **10a**, **10b**, **10c** and **10d**.

|  |  |  |
| --- | --- | --- |
| **10a1** (70.8%) | **10a2** (27.2%) | **10b1** (28.5%) |
|  |  |  |
| **10b2** (64.5%) | **10b3** (5.5%) | **10c1**(90.1%) |
|  |  |  |
| **10c2** (8.4%) | **10d1** (98.7%) | **10d2** (1.3%) |

**Table S10a**. Stable conformers of paralemnanoid J (**10**).

| **10a1** | | | | **10a2** | | | |
| --- | --- | --- | --- | --- | --- | --- | --- |
| C | -2.85838 | 1.32884 | 0.6203 | C | 3.01313 | 1.30370 | -0.42111 |
| C | -3.9352 | 0.41796 | 0.20597 | C | 3.96611 | 0.19598 | -0.29039 |
| C | -3.49589 | -0.98428 | -0.1694 | C | 3.387 | -1.14095 | 0.14021 |
| C | -2.09085 | -1.01622 | -0.79133 | C | 2.00685 | -1.04918 | 0.81975 |
| C | -1.01248 | -0.35005 | 0.13674 | C | 1.00461 | -0.19990 | -0.05282 |
| C | -1.54865 | 1.00477 | 0.62172 | C | 1.67857 | 1.17286 | -0.27278 |
| C | 0.31282 | -0.15976 | -0.72448 | C | -0.35114 | 0.03356 | 0.70046 |
| C | 1.19722 | 1.07762 | -0.40825 | C | -1.28036 | 1.11257 | 0.06444 |
| C | 0.50031 | 2.3262 | 0.11673 | C | -0.59019 | 2.12900 | -0.84908 |
| C | -0.53919 | 1.97177 | 1.17988 | C | 0.81034 | 2.41009 | -0.31065 |
| C | 1.36986 | -1.30338 | -0.6215 | C | -1.32946 | -1.17134 | 0.84191 |
| C | 2.46225 | -0.73105 | 0.31478 | C | -2.4677 | -0.89083 | -0.16031 |
| O | 2.11814 | 0.63621 | 0.57707 | O | -2.25642 | 0.42096 | -0.68792 |
| C | -1.76706 | -2.44103 | -1.26174 | C | 1.57165 | -2.46630 | 1.22633 |
| C | -0.77942 | -1.18564 | 1.42919 | C | 0.77186 | -0.86403 | -1.43758 |
| O | -5.1109 | 0.75879 | 0.20851 | O | 5.16457 | 0.34109 | -0.49681 |
| C | 1.95451 | -1.74383 | -1.9713 | C | -1.86465 | -1.33688 | 2.27103 |
| H | -0.00426 | -0.04661 | -1.76449 | H | -0.09308 | 0.40222 | 1.69808 |
| O | 2.49859 | -1.45154 | 1.51107 | O | -2.42489 | -1.83638 | -1.19015 |
| C | 3.54705 | -1.05729 | 2.38791 | C | -3.48497 | -1.71367 | -2.12937 |
| O | 1.87597 | 1.34965 | -1.63812 | O | -1.87598 | 1.75687 | 1.19957 |
| C | 2.90931 | 2.32598 | -1.57895 | C | -3.02246 | 2.55897 | 0.94511 |
| H | -3.18587 | 2.29804 | 0.98981 | H | 3.45494 | 2.27629 | -0.62657 |
| H | -4.2393 | -1.40493 | -0.85362 | H | 4.11484 | -1.61266 | 0.80875 |
| H | -3.52798 | -1.60097 | 0.74034 | H | 3.33531 | -1.78712 | -0.74668 |
| H | -2.13412 | -0.38042 | -1.68759 | H | 2.14376 | -0.47457 | 1.74721 |
| H | 1.2488 | 3.01128 | 0.52616 | H | -0.54367 | 1.73254 | -1.86592 |
| H | 0.0155 | 2.82386 | -0.73 | H | -1.17567 | 3.05079 | -0.89722 |
| H | -0.0242 | 1.51799 | 2.03592 | H | 1.30907 | 3.19802 | -0.88292 |
| H | -1.04045 | 2.87318 | 1.54398 | H | 0.70648 | 2.79170 | 0.71615 |
| H | 0.95922 | -2.1811 | -0.12327 | H | -0.8681 | -2.10419 | 0.52196 |
| H | 3.46177 | -0.74718 | -0.15003 | H | -3.46526 | -0.91201 | 0.30947 |
| H | -2.54003 | -2.77948 | -1.95958 | H | 2.40167 | -2.95723 | 1.74591 |
| H | -1.75023 | -3.15361 | -0.43076 | H | 1.32409 | -3.08922 | 0.36035 |
| H | -0.80984 | -2.50557 | -1.78276 | H | 0.72158 | -2.47280 | 1.90962 |
| H | -1.65882 | -1.13734 | 2.07705 | H | 1.70109 | -0.91963 | -2.00872 |
| H | -0.5892 | -2.23812 | 1.21058 | H | 0.36977 | -1.87425 | -1.33176 |
| H | 0.08131 | -0.82034 | 1.99108 | H | 0.04852 | -0.30713 | -2.03453 |
| H | 1.18049 | -2.16812 | -2.61842 | H | -1.05554 | -1.56292 | 2.97281 |
| H | 2.71849 | -2.51538 | -1.82443 | H | -2.59034 | -2.15552 | 2.32549 |
| H | 2.40482 | -0.89892 | -2.49625 | H | -2.34938 | -0.41643 | 2.60766 |
| H | 3.52279 | -1.74284 | 3.23718 | H | -3.38738 | -2.54802 | -2.8267 |
| H | 4.52706 | -1.1325 | 1.89383 | H | -4.46512 | -1.77404 | -1.63293 |
| H | 3.409 | -0.03034 | 2.7441 | H | -3.42629 | -0.76885 | -2.6811 |
| H | 3.4598 | 2.24823 | -2.51909 | H | -3.42092 | 2.83710 | 1.92311 |
| H | 2.51114 | 3.34537 | -1.4925 | H | -2.78054 | 3.47902 | 0.39608 |
| H | 3.59235 | 2.13737 | -0.74316 | H | -3.78326 | 2.00657 | 0.3839 |
| **10b1** | | | | **10b2** | | | |
| C | -2.89935 | 0.91619 | -0.81514 | C | -2.83041 | 1.08737 | -0.74648 |
| C | -3.80218 | -0.17845 | -0.42317 | C | -3.8151 | 0.04208 | -0.42121 |
| C | -3.29394 | -1.08951 | 0.68071 | C | -3.38426 | -0.96712 | 0.62859 |
| C | -1.7712 | -1.2937 | 0.62621 | C | -1.87951 | -1.27795 | 0.57194 |
| C | -0.99427 | 0.0673 | 0.62244 | C | -1.00629 | 0.02043 | 0.65044 |
| C | -1.64907 | 1.07083 | -0.33225 | C | -1.57637 | 1.11914 | -0.2502 |
| C | 0.50971 | -0.17069 | 0.24198 | C | 0.48028 | -0.30551 | 0.26779 |
| C | 1.31999 | 1.09255 | -0.15817 | C | 1.38323 | 0.89620 | -0.10391 |
| C | 0.5726 | 2.03398 | -1.10939 | C | 0.70324 | 1.92986 | -1.01334 |
| C | -0.85914 | 2.32266 | -0.64198 | C | -0.68747 | 2.31842 | -0.49567 |
| C | 0.91634 | -1.19619 | -0.8554 | C | 0.8324 | -1.34398 | -0.83787 |
| C | 2.40339 | -0.83478 | -1.00077 | C | 2.34353 | -1.08964 | -0.92503 |
| O | 2.48219 | 0.57757 | -0.84131 | O | 2.48756 | 0.32735 | -0.83118 |
| C | -1.32765 | -2.27993 | 1.71847 | C | -1.5204 | -2.35137 | 1.61169 |
| C | -1.03027 | 0.712 | 2.03971 | C | -1.00943 | 0.58458 | 2.10275 |
| O | -4.91444 | -0.30879 | -0.91701 | O | -4.93004 | 0.02484 | -0.92597 |
| C | 0.22379 | -1.15092 | -2.23044 | C | 0.19109 | -1.21175 | -2.23188 |
| H | 0.98921 | -0.55821 | 1.14601 | H | 0.94577 | -0.70990 | 1.17104 |
| O | 3.14434 | -1.52227 | -0.02035 | O | 2.97513 | -1.77656 | 0.12367 |
| C | 4.55141 | -1.38107 | -0.17326 | C | 4.38167 | -1.58845 | 0.17159 |
| O | 1.71917 | 1.9164 | 0.9153 | O | 1.83235 | 1.46332 | 1.11315 |
| C | 2.52051 | 1.3369 | 1.93945 | C | 2.83617 | 2.46829 | 1.02315 |
| H | -3.32524 | 1.65003 | -1.49576 | H | -3.19488 | 1.88759 | -1.38683 |
| H | -3.82205 | -2.04519 | 0.60865 | H | -3.97951 | -1.87590 | 0.49663 |
| H | -3.589 | -0.64262 | 1.64101 | H | -3.65581 | -0.55769 | 1.61225 |
| H | -1.56649 | -1.77346 | -0.33667 | H | -1.70051 | -1.71660 | -0.41533 |
| H | 1.15027 | 2.95974 | -1.18346 | H | 1.33821 | 2.81489 | -1.11719 |
| H | 0.55103 | 1.58288 | -2.1024 | H | 0.61108 | 1.50096 | -2.01245 |
| H | -1.38959 | 2.9173 | -1.392 | H | -1.17426 | 3.00226 | -1.19754 |
| H | -0.813 | 2.93827 | 0.26594 | H | -0.57549 | 2.86778 | 0.44867 |
| H | 0.84276 | -2.21183 | -0.45828 | H | 0.66367 | -2.35571 | -0.46105 |
| H | 2.81545 | -1.06045 | -1.99504 | H | 2.7973 | -1.37408 | -1.88611 |
| H | -0.24534 | -2.44076 | 1.72477 | H | -0.45001 | -2.57660 | 1.63073 |
| H | -1.62366 | -1.94408 | 2.71725 | H | -1.81819 | -2.05536 | 2.6224 |
| H | -1.80178 | -3.25253 | 1.55142 | H | -2.04826 | -3.28204 | 1.3789 |
| H | -0.47677 | 1.65178 | 2.05863 | H | -0.37426 | 1.46682 | 2.18474 |
| H | -2.05756 | 0.91454 | 2.35562 | H | -2.02136 | 0.85346 | 2.4194 |
| H | -0.57248 | 0.04783 | 2.7776 | H | -0.61349 | -0.15538 | 2.80292 |
| H | 0.47414 | -2.05577 | -2.79416 | H | 0.36531 | -2.12823 | -2.80527 |
| H | -0.8634 | -1.09572 | -2.15791 | H | -0.88693 | -1.04691 | -2.1902 |
| H | 0.55432 | -0.29943 | -2.83016 | H | 0.6271 | -0.38865 | -2.80378 |
| H | 5.01825 | -1.96587 | 0.62185 | H | 4.75736 | -2.21755 | 0.98093 |
| H | 4.88467 | -1.77055 | -1.14648 | H | 4.85858 | -1.89565 | -0.77151 |
| H | 4.865 | -0.33404 | -0.08826 | H | 4.6405 | -0.54307 | 0.37244 |
| H | 3.03877 | 2.1657 | 2.42726 | H | 3.22845 | 2.59600 | 2.03444 |
| H | 1.91324 | 0.81546 | 2.69024 | H | 3.64883 | 2.16132 | 0.35715 |
| H | 3.25508 | 0.64077 | 1.52737 | H | 2.43642 | 3.43299 | 0.68323 |
| **10b3** | | | | **10c1** | | | |
| C | -2.8514 | 0.96525 | -0.94866 | C | 2.97111 | -1.26536 | -0.63294 |
| C | -3.8851 | 0.10596 | -0.35313 | C | 3.94942 | -0.17250 | -0.54588 |
| C | -3.4379 | -0.83686 | 0.75461 | C | 3.53754 | 0.98649 | 0.3353 |
| C | -1.9454 | -1.2075 | 0.69713 | C | 2.03717 | 1.32461 | 0.24627 |
| C | -1.0421 | 0.06614 | 0.56037 | C | 1.10768 | 0.07845 | 0.4848 |
| C | -1.5533 | 0.94912 | -0.58066 | C | 1.72168 | -1.20106 | -0.13102 |
| C | 0.4458 | -0.32742 | 0.3263 | C | -0.23221 | 0.24662 | -0.32983 |
| C | 1.4036 | 0.87067 | 0.04624 | C | -1.17081 | -0.97980 | -0.28282 |
| C | 0.7307 | 2.13345 | -0.52341 | C | -0.50430 | -2.19888 | -0.90756 |
| C | -0.5517 | 1.83011 | -1.30632 | C | 0.84131 | -2.43689 | -0.19505 |
| C | 0.8242 | -1.38387 | -0.76106 | C | -1.23940 | 1.41525 | -0.16822 |
| C | 2.3035 | -1.03526 | -0.96202 | C | -2.54339 | 0.81118 | -0.75631 |
| O | 2.3377 | 0.39066 | -0.93306 | O | -2.29608 | -0.57119 | -1.04066 |
| C | -1.5864 | -2.13098 | 1.87116 | C | 1.76834 | 2.50707 | 1.19751 |
| C | -1.1190 | 0.90288 | 1.87334 | C | 0.92717 | -0.14072 | 2.00851 |
| O | -5.0525 | 0.1551 | -0.71989 | O | 5.03991 | -0.22845 | -1.09879 |
| C | 0.1155 | -1.37531 | -2.1268 | C | -0.91160 | 2.71523 | -0.91641 |
| H | 0.8159 | -0.74294 | 1.26613 | H | 0.09517 | 0.28444 | -1.3789 |
| O | 3.0526 | -1.63855 | 0.05738 | O | -3.59214 | 1.00307 | 0.15134 |
| C | 4.4499 | -1.40856 | -0.03433 | C | -4.87658 | 0.72723 | -0.39051 |
| O | 2.0637 | 1.14159 | 1.27237 | O | -1.53786 | -1.20153 | 1.07261 |
| C | 3.0705 | 2.1446 | 1.23232 | C | -2.60989 | -2.10694 | 1.29983 |
| H | -3.1991 | 1.62886 | -1.73779 | H | 3.32844 | -2.17349 | -1.1133 |
| H | -4.0655 | -1.73245 | 0.70347 | H | 3.79672 | 0.71334 | 1.36874 |
| H | -3.6789 | -0.35566 | 1.71266 | H | 4.14906 | 1.85685 | 0.07927 |
| H | -1.8042 | -1.788 | -0.22146 | H | 1.85187 | 1.66178 | -0.78265 |
| H | 0.5031 | 2.81291 | 0.3025 | H | -1.14108 | -3.08589 | -0.83701 |
| H | 1.4437 | 2.6472 | -1.17514 | H | -0.34660 | -1.99848 | -1.97182 |
| H | -0.2808 | 1.33067 | -2.24386 | H | 1.38864 | -3.24593 | -0.68683 |
| H | -1.0367 | 2.76592 | -1.60353 | H | 0.63164 | -2.77434 | 0.82768 |
| H | 0.7463 | -2.38567 | -0.32975 | H | -1.43241 | 1.61855 | 0.88918 |
| H | 2.7057 | -1.31728 | -1.9463 | H | -2.81270 | 1.26063 | -1.72378 |
| H | -0.5385 | -2.44359 | 1.85472 | H | 0.70867 | 2.73535 | 1.32028 |
| H | -1.7819 | -1.65824 | 2.83883 | H | 2.26432 | 3.40761 | 0.82031 |
| H | -2.1991 | -3.03747 | 1.82583 | H | 2.17450 | 2.30769 | 2.19387 |
| H | -0.5203 | 1.8114 | 1.81763 | H | 1.90459 | -0.13479 | 2.50185 |
| H | -2.1482 | 1.19857 | 2.08989 | H | 0.31861 | 0.64987 | 2.45302 |
| H | -0.7352 | 0.32535 | 2.71851 | H | 0.43297 | -1.08226 | 2.23148 |
| H | 0.3819 | -2.28315 | -2.6784 | H | -1.75437 | 3.41096 | -0.84198 |
| H | -0.9722 | -1.3448 | -2.05034 | H | -0.03131 | 3.22601 | -0.52764 |
| H | 0.4322 | -0.52461 | -2.73551 | H | -0.73817 | 2.51609 | -1.98042 |
| H | 4.9176 | -1.97995 | 0.76984 | H | -5.60558 | 0.95627 | 0.38939 |
| H | 4.8494 | -1.75588 | -0.99931 | H | -5.08313 | 1.35602 | -1.26945 |
| H | 4.6959 | -0.34771 | 0.08544 | H | -4.97694 | -0.32523 | -0.68224 |
| H | 2.6538 | 3.1519 | 1.09677 | H | -2.84291 | -2.03863 | 2.36445 |
| H | 3.5778 | 2.10738 | 2.19871 | H | -3.49423 | -1.83233 | 0.71865 |
| H | 3.7975 | 1.95224 | 0.43554 | H | -2.33580 | -3.14507 | 1.06792 |
| **10c2** | | | | **10d1** | | | |
| C | -2.82651 | 1.15220 | -0.77753 | C | -2.65557 | -1.28740 | -0.54790 |
| C | -3.53554 | -0.10201 | -1.08850 | C | -3.62706 | -0.43012 | 0.14005 |
| C | -2.78860 | -1.36460 | -0.69249 | C | -3.25739 | 1.03611 | 0.23668 |
| C | -2.11879 | -1.20539 | 0.68417 | C | -1.74499 | 1.31871 | 0.34575 |
| C | -1.11749 | 0.00836 | 0.71949 | C | -0.90888 | 0.56129 | -0.73661 |
| C | -1.70092 | 1.21751 | -0.03570 | C | -1.43558 | -0.87300 | -0.93957 |
| C | 0.19974 | -0.29106 | -0.08196 | C | 0.63060 | 0.41687 | -0.40872 |
| C | 1.13846 | 0.93169 | -0.27608 | C | 1.02791 | -0.94974 | 0.17578 |
| C | 0.43439 | 2.11422 | -0.93548 | C | 0.81986 | -2.07313 | -0.84411 |
| C | -0.83326 | 2.45472 | -0.10882 | C | -0.49853 | -1.83762 | -1.63540 |
| C | 1.25276 | -1.36187 | 0.28604 | C | 1.52872 | 1.38749 | 0.42280 |
| C | 2.40282 | -0.96099 | -0.67001 | C | 2.82033 | 0.54658 | 0.40501 |
| O | 2.17276 | 0.40034 | -1.08542 | O | 2.40935 | -0.82152 | 0.51437 |
| C | -3.22314 | -1.14776 | 1.76137 | C | -1.55931 | 2.84863 | 0.32168 |
| C | -0.82804 | 0.37134 | 2.19689 | C | -1.03405 | 1.29122 | -2.10419 |
| O | -4.59270 | -0.12022 | -1.70345 | O | -4.68988 | -0.86383 | 0.56683 |
| C | 0.89659 | -2.84088 | 0.12141 | C | 1.17993 | 1.72527 | 1.88300 |
| H | -0.12673 | -0.52477 | -1.10476 | H | 1.12487 | 0.42602 | -1.38723 |
| O | 3.62899 | -1.11792 | -0.01839 | O | 3.50015 | 0.78329 | -0.80143 |
| C | 4.75516 | -1.00952 | -0.88131 | C | 4.72516 | 0.07272 | -0.91929 |
| O | 1.65603 | 1.31645 | 0.99804 | O | 0.25351 | -1.15231 | 1.34956 |
| C | 2.85714 | 2.07992 | 0.99381 | C | 0.61205 | -2.25590 | 2.17288 |
| H | -3.20771 | 2.03848 | -1.28068 | H | -2.97071 | -2.31505 | -0.71323 |
| H | -3.48575 | -2.20700 | -0.70361 | H | -3.79343 | 1.47054 | 1.08621 |
| H | -2.04307 | -1.55789 | -1.47599 | H | -3.66529 | 1.52245 | -0.66197 |
| H | -1.53448 | -2.10830 | 0.88312 | H | -1.41430 | 0.92961 | 1.31403 |
| H | 1.09980 | 2.98062 | -0.99810 | H | 0.79814 | -3.04303 | -0.34156 |
| H | 0.15417 | 1.84097 | -1.95749 | H | 1.67627 | -2.08415 | -1.52257 |
| H | -1.38049 | 3.27112 | -0.58839 | H | -0.24607 | -1.43446 | -2.62398 |
| H | -0.52488 | 2.79617 | 0.88416 | H | -1.01454 | -2.78436 | -1.81362 |
| H | 1.61755 | -1.18188 | 1.30118 | H | 1.71288 | 2.31023 | -0.13553 |
| H | 2.39978 | -1.55835 | -1.59575 | H | 3.49102 | 0.74060 | 1.25418 |
| H | -2.82284 | -1.22675 | 2.77390 | H | -0.51735 | 3.16160 | 0.23363 |
| H | -3.91448 | -1.98438 | 1.61777 | H | -1.96346 | 3.29000 | 1.23896 |
| H | -3.80973 | -0.22540 | 1.70107 | H | -2.10418 | 3.29580 | -0.51591 |
| H | -1.72424 | 0.76294 | 2.68322 | H | -2.08068 | 1.45849 | -2.37380 |
| H | -0.51452 | -0.52369 | 2.74537 | H | -0.52933 | 2.26095 | -2.07654 |
| H | -0.03047 | 1.10305 | 2.28693 | H | -0.57598 | 0.71003 | -2.90918 |
| H | 1.80677 | -3.44726 | 0.17973 | H | 1.99664 | 2.31471 | 2.31528 |
| H | 0.22508 | -3.19337 | 0.90787 | H | 0.27241 | 2.31829 | 1.97854 |
| H | 0.42106 | -3.04023 | -0.84591 | H | 1.05511 | 0.82116 | 2.47961 |
| H | 5.64041 | -1.20112 | -0.27179 | H | 5.41649 | 0.33234 | -0.10355 |
| H | 4.70949 | -1.75234 | -1.69102 | H | 4.56405 | -1.01104 | -0.91133 |
| H | 4.82731 | -0.00907 | -1.32378 | H | 5.17212 | 0.36813 | -1.87061 |
| H | 3.04114 | 2.35142 | 2.03555 | H | 0.04328 | -2.14216 | 3.09790 |
| H | 3.69758 | 1.49179 | 0.61695 | H | 0.34483 | -3.21974 | 1.72038 |
| H | 2.76992 | 3.00093 | 0.40262 | H | 1.68308 | -2.24989 | 2.40018 |
| **10d2** | | | |  |  |  |  |
| C | 2.78923 | 0.99139 | -0.65682 |  |  |  |  |
| C | 3.65370 | 0.09796 | 0.12166 |  |  |  |  |
| C | 3.10793 | -1.29381 | 0.36923 |  |  |  |  |
| C | 1.57284 | -1.37805 | 0.49372 |  |  |  |  |
| C | 0.83256 | -0.64505 | -0.67147 |  |  |  |  |
| C | 1.52670 | 0.68862 | -1.01486 |  |  |  |  |
| C | -0.67728 | -0.28173 | -0.38119 |  |  |  |  |
| C | -0.90445 | 1.17015 | 0.07302 |  |  |  |  |
| C | -0.55930 | 2.16322 | -1.04104 |  |  |  |  |
| C | 0.70652 | 1.68411 | -1.80708 |  |  |  |  |
| C | -1.69762 | -1.05019 | 0.51410 |  |  |  |  |
| C | -2.87366 | -0.04866 | 0.38294 |  |  |  |  |
| O | -2.29574 | 1.24080 | 0.39224 |  |  |  |  |
| C | 1.20334 | -2.86835 | 0.63005 |  |  |  |  |
| C | 0.86933 | -1.52282 | -1.95455 |  |  |  |  |
| O | 4.76471 | 0.43995 | 0.50700 |  |  |  |  |
| C | -1.41412 | -1.28361 | 2.00816 |  |  |  |  |
| H | -1.16246 | -0.31467 | -1.36416 |  |  |  |  |
| O | -3.57837 | -0.17886 | -0.83989 |  |  |  |  |
| C | -4.61597 | -1.14112 | -0.80761 |  |  |  |  |
| O | -0.12248 | 1.38096 | 1.23942 |  |  |  |  |
| C | -0.34212 | 2.59481 | 1.94924 |  |  |  |  |
| H | 3.22451 | 1.95192 | -0.92249 |  |  |  |  |
| H | 3.59113 | -1.69780 | 1.26430 |  |  |  |  |
| H | 3.44968 | -1.91833 | -0.46967 |  |  |  |  |
| H | 1.29343 | -0.85072 | 1.41150 |  |  |  |  |
| H | -0.40213 | 3.16146 | -0.62574 |  |  |  |  |
| H | -1.41599 | 2.22810 | -1.71569 |  |  |  |  |
| H | 0.39008 | 1.21478 | -2.74692 |  |  |  |  |
| H | 1.33253 | 2.53551 | -2.08562 |  |  |  |  |
| H | -1.97819 | -1.99909 | 0.04341 |  |  |  |  |
| H | -3.58606 | -0.11331 | 1.21886 |  |  |  |  |
| H | 1.55693 | -3.25584 | 1.59148 |  |  |  |  |
| H | 1.68382 | -3.46502 | -0.15205 |  |  |  |  |
| H | 0.13045 | -3.05908 | 0.56952 |  |  |  |  |
| H | 1.88810 | -1.84238 | -2.19118 |  |  |  |  |
| H | 0.25125 | -2.41674 | -1.83256 |  |  |  |  |
| H | 0.48565 | -0.97718 | -2.82103 |  |  |  |  |
| H | -2.30572 | -1.71692 | 2.47653 |  |  |  |  |
| H | -0.59170 | -1.97415 | 2.18563 |  |  |  |  |
| H | -1.18169 | -0.34666 | 2.51461 |  |  |  |  |
| H | -4.23924 | -2.16559 | -0.66979 |  |  |  |  |
| H | -5.33949 | -0.92869 | -0.00584 |  |  |  |  |
| H | -5.12962 | -1.08686 | -1.76963 |  |  |  |  |
| H | 0.19123 | 2.49330 | 2.89656 |  |  |  |  |
| H | 0.06038 | 3.46725 | 1.41877 |  |  |  |  |
| H | -1.40758 | 2.75273 | 2.14588 |  |  |  |  |

**Table S10b**.Optimized Z-Matrixes of paralemnanoid J (**10**) with simplified structures in the Gas Phase (Å) at B3LYP/6-31+G(d,p) level.

**Figure S18c**. Experimental and calculated ECD spectra of paralemnanoid J (**10**)

**Figure S19a**. Structures of isomers **11a** and **11b** for paralemnanoid K (**11**).

**Figure S19b**. DP4+ results obtained using experimental data of paralemnanoid K (**11**) *versus* isomers **11a** and **11b**.

|  |  |  |
| --- | --- | --- |
| **11a1** (64.5%) | **11a2** (35.5%) | **11b1** (98.6%) |
|  |  |  |
| **11a2** (1.1%) |  |  |

**Table S20a**. Stable conformers of paralemnanoid K (**11**).

| **11a1** | | | | **11a2** | | | |
| --- | --- | --- | --- | --- | --- | --- | --- |
| C | 1.01552 | 1.04672 | 0.43044 | C | 1.01799 | 1.06328 | 0.42359 |
| C | 2.33174 | 1.12191 | 0.14903 | C | 2.33924 | 1.1165 | 0.16057 |
| C | 3.18294 | -0.04926 | -0.1273 | C | 3.17249 | -0.06815 | -0.11415 |
| C | 2.50947 | -1.41349 | -0.05375 | C | 2.46943 | -1.42015 | -0.08877 |
| C | 1.00643 | -1.35935 | -0.3872 | C | 0.97118 | -1.3221 | -0.43217 |
| C | 0.27718 | -0.29823 | 0.51285 | C | 0.26091 | -0.27138 | 0.4926 |
| C | -1.16028 | -0.10093 | 0.05491 | C | -1.18395 | -0.05736 | 0.06203 |
| C | -1.67541 | 1.08769 | -0.29214 | C | -1.68942 | 1.14071 | -0.26512 |
| C | -1.0093 | 2.41753 | -0.20119 | C | -0.99355 | 2.45765 | -0.19912 |
| C | 0.21382 | 2.29175 | 0.72797 | C | 0.23511 | 2.31965 | 0.72042 |
| O | 4.37569 | 0.07007 | -0.37425 | O | 4.37369 | 0.0298 | -0.32744 |
| C | -2.25549 | -1.15442 | -0.12112 | C | -2.25089 | -1.11562 | -0.21818 |
| C | -3.26844 | -0.345 | -0.9704 | C | -3.47079 | -0.21091 | -0.54569 |
| O | -2.96001 | 1.05643 | -0.76819 | O | -2.98176 | 1.13953 | -0.71692 |
| C | 0.81452 | -1.12725 | -1.89541 | C | 0.80398 | -1.0342 | -1.93488 |
| C | 0.31794 | -0.76389 | 2.00051 | C | 0.30779 | -0.74885 | 1.97734 |
| C | -2.8827 | -1.66592 | 1.19073 | C | -2.58465 | -2.1211 | 0.89456 |
| H | 2.84012 | 2.08287 | 0.11614 | H | 2.8659 | 2.06797 | 0.14267 |
| H | 2.67172 | -1.79653 | 0.96205 | H | 2.61691 | -1.84076 | 0.91416 |
| H | 3.04496 | -2.09156 | -0.72512 | H | 2.99601 | -2.08624 | -0.77911 |
| H | 0.56959 | -2.33695 | -0.14278 | H | 0.50893 | -2.29663 | -0.22479 |
| H | -1.7159 | 3.16249 | 0.18243 | H | -1.67991 | 3.22315 | 0.17985 |
| H | -0.70725 | 2.75924 | -1.20007 | H | -0.69196 | 2.7774 | -1.20544 |
| H | 0.85071 | 3.17788 | 0.65593 | H | 0.88331 | 3.19687 | 0.63964 |
| H | -0.14883 | 2.23856 | 1.76371 | H | -0.12045 | 2.27682 | 1.75922 |
| H | -1.90552 | -2.02013 | -0.69715 | H | -1.9705 | -1.69801 | -1.10903 |
| H | -3.16146 | -0.56003 | -2.04116 | H | -4.0001 | -0.49844 | -1.45768 |
| H | -4.30966 | -0.50452 | -0.67862 | H | -4.18593 | -0.20486 | 0.28748 |
| H | -0.23855 | -1.15055 | -2.18433 | H | -0.24469 | -0.99769 | -2.23706 |
| H | 1.33642 | -1.90539 | -2.46198 | H | 1.29455 | -1.81849 | -2.52066 |
| H | 1.22104 | -0.16075 | -2.20843 | H | 1.26045 | -0.07983 | -2.21388 |
| H | -0.0914 | -1.7729 | 2.10218 | H | -0.14029 | -1.73964 | 2.08483 |
| H | 1.3381 | -0.77371 | 2.39279 | H | 1.33187 | -0.79584 | 2.35627 |
| H | -0.27695 | -0.09693 | 2.63019 | H | -0.25305 | -0.06037 | 2.61541 |
| H | -3.7376 | -2.31709 | 0.97506 | H | -3.46692 | -2.71103 | 0.61987 |
| H | -2.17383 | -2.24313 | 1.78913 | H | -1.76755 | -2.82691 | 1.06674 |
| H | -3.23874 | -0.83001 | 1.80199 | H | -2.80144 | -1.6099 | 1.83822 |
| **11b1** | | | | **11b2** | | | |
| C | -0.94948 | 1.23328 | 0.20594 | C | -0.87585 | 1.24376 | 0.35101 |
| C | -2.17515 | 1.34792 | -0.34244 | C | -1.95829 | 1.3182 | -0.44943 |
| C | -3.08609 | 0.21747 | -0.56564 | C | -2.56611 | 0.1442 | -1.10253 |
| C | -2.62191 | -1.10734 | 0.0008 | C | -1.76481 | -1.13906 | -0.95922 |
| C | -1.09539 | -1.30345 | -0.05922 | C | -1.20798 | -1.2832 | 0.46621 |
| C | -0.30846 | -0.11222 | 0.60037 | C | -0.25976 | -0.08487 | 0.81615 |
| C | 1.15599 | -0.05233 | 0.14336 | C | 1.10986 | -0.13914 | 0.12246 |
| C | 1.78753 | 1.09009 | -0.1784 | C | 1.74317 | 0.99336 | -0.23515 |
| C | 1.20751 | 2.45689 | -0.28856 | C | 1.23457 | 2.40015 | -0.18999 |
| C | -0.1342 | 2.48148 | 0.46417 | C | -0.0637 | 2.47782 | 0.65438 |
| O | -4.16625 | 0.35247 | -1.12503 | O | -3.58043 | 0.22501 | -1.78126 |
| C | 2.18066 | -1.18376 | -0.02683 | C | 2.12795 | -1.28214 | 0.0486 |
| C | 3.4852 | -0.36026 | 0.00338 | C | 3.27133 | -0.5797 | -0.72929 |
| O | 3.12673 | 0.96608 | -0.44939 | O | 3.03764 | 0.84294 | -0.65421 |
| C | -0.77831 | -2.69263 | 0.52218 | C | -2.3742 | -1.47701 | 1.4568 |
| C | -0.34982 | -0.23698 | 2.15231 | C | -0.01061 | -0.0682 | 2.35308 |
| C | 2.05608 | -1.94918 | -1.3608 | C | 1.7469 | -2.59827 | -0.64797 |
| H | -2.56222 | 2.32313 | -0.62914 | H | -2.36559 | 2.28379 | -0.74173 |
| H | -2.96616 | -1.15353 | 1.04446 | H | -2.39533 | -1.9868 | -1.24094 |
| H | -3.139 | -1.91188 | -0.53086 | H | -0.93718 | -1.08317 | -1.68009 |
| H | -0.8151 | -1.29895 | -1.12037 | H | -0.60475 | -2.19415 | 0.50915 |
| H | 1.90416 | 3.1965 | 0.12118 | H | 2.01339 | 3.05474 | 0.218 |
| H | 1.06108 | 2.7143 | -1.34612 | H | 1.03707 | 2.74903 | -1.21207 |
| H | 0.07533 | 2.55737 | 1.53882 | H | 0.1973 | 2.53771 | 1.71561 |
| H | -0.71409 | 3.36737 | 0.19164 | H | -0.62313 | 3.38267 | 0.40188 |
| H | 2.17668 | -1.8946 | 0.8072 | H | 2.4646 | -1.53077 | 1.06804 |
| H | 3.88343 | -0.27271 | 1.02314 | H | 4.26917 | -0.78264 | -0.33274 |
| H | 4.26658 | -0.74372 | -0.65716 | H | 3.24589 | -0.87076 | -1.78825 |
| H | 0.28829 | -2.91306 | 0.57655 | H | -2.02954 | -1.7723 | 2.45036 |
| H | -1.24266 | -3.46501 | -0.1 | H | -3.035 | -2.27021 | 1.09243 |
| H | -1.19057 | -2.80217 | 1.53043 | H | -2.97916 | -0.57131 | 1.5647 |
| H | -1.37617 | -0.33292 | 2.52185 | H | -0.91539 | 0.1837 | 2.91307 |
| H | 0.21504 | -1.11123 | 2.48653 | H | 0.32615 | -1.05552 | 2.68674 |
| H | 0.09927 | 0.639 | 2.62555 | H | 0.76902 | 0.64782 | 2.6195 |
| H | 2.86681 | -2.6807 | -1.45676 | H | 2.64374 | -3.20828 | -0.80867 |
| H | 1.11188 | -2.48831 | -1.45408 | H | 1.055 | -3.20155 | -0.05604 |
| H | 2.12864 | -1.25115 | -2.2011 | H | 1.2914 | -2.41101 | -1.62575 |

**Table S11b**.Optimized Z-Matrixes of paralemnanoid K (**11**) with simplified structures in the Gas Phase (Å) at B3LYP/6-31+G(d,p) level
